# Fullerene-Doped Poly(ionic liquids) as Small Molecular Gas Sensors—Control of Intermolecular Interactions

**DOI:** 10.1021/acsomega.4c08941

**Published:** 2024-12-23

**Authors:** Jaroslav Otta, Jakub Mikuláštík, Richard Šípka, Matthias Stein, Irina A. Kühne, Martin Vrňata, Jan Vlček

**Affiliations:** † Department of Functional Materials, FZU - Institute of Physics - Czech Academy of Sciences, Na Slovance 1999/2, Prague 8 182 00, Czech Republic; ‡ Department of Physics and Measurements, University of Chemistry and Technology, Technicka 5, Prague 6 16 628, Czech Republic; § Molecular Simulations and Design Group, Max Planck Institute for Dynamics of Complex Technical Systems, Sandtorstrasse 1, Magdeburg 39106, Germany

## Abstract

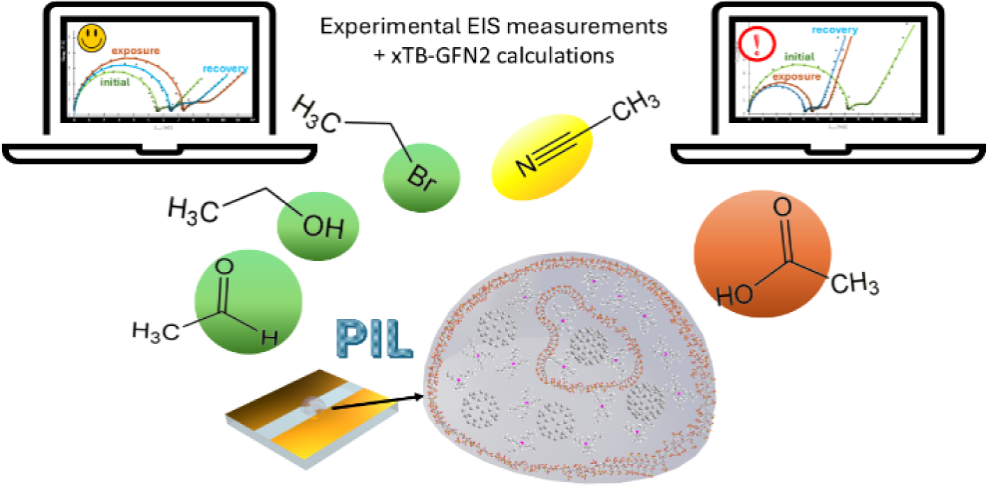

Here, we investigate the interactions between five representative gaseous analytes and two poly(ionic liquids) (PILs) based on the sulfopropyl acrylate polyanion in combination with the alkylphosphonium cations, P_4,4,4,4_ and P_4,4,4,8_, and their nanocomposites with fullerenes (C_60_, C_70_) to reveal the potential of PILs as sensitive layers for gas sensors. The gaseous analytes were chosen based on their molecular size (all of them containing two carbon atoms) and variation of functional groups: alcohol (ethanol), nitrile (acetonitrile), aldehyde (acetaldehyde), halogenated alkane (bromoethane), and carboxylic acid (acetic acid). The six variations of PILs—P_4,4,4,4_SPA (**1**), P_4,4,4,4_SPA + C_60_ (**1** + C_60_), P_4,4,4,4_SPA + C_70_ (**1** + C_70_), and P_4,4,4,8_SPA (**2**), P_4,4,4,8_SPA + C_60_ (**2** + C_60_), P_4,4,4,8_SPA + C_70_ (**2** + C_70_)—were characterized by UV–vis and Raman spectroscopy, and their interactions with each gaseous analyte were studied using electrochemical impedance spectroscopy. Exposure of all PIL samples to acetaldehyde, bromoethane, and ethanol leads to a decrease in the diffusion coefficient, while exposure to acetic acid reveals an increase. Fullerene-doping significantly enhances the response to the analyte. Semiempirical quantum mechanical xTB-GFN2 calculations revealed that hydrogen bonding and proton transfer events play an important role during the detection process.

## Introduction

Poly(ionic liquids) combine the properties of both polymers and ionic liquids. This unique combination imparts characteristics such as high thermal stability, low volatility, and good ionic conductivity (which can be mediated by ions of one sign), making them valuable in various applications, including energy storage devices^1^ and as electrolytes in batteries.^2^ The tunable nature of polymers allows for the customization of PILs with specific properties, enhancing their versatility in different technological fields. Currently, PILs are objects of intensive basic research, but they already have many industrial applications, such as water purification,^3,4^ energy storage,^5−7^ catalytic cycloadditions,^8^ antibacterial applications,^7,9,10^ supercapacitors,^11−13^ biosensors,^9,14,15^ and catalysis.^8,16^ Recent studies have also explored their gas absorption mechanisms^17^ and structural stability, highlighting their potential in gas separation and storage applications.^18,19^ Lately, PILs have also emerged as promising materials for chemical gas sensors^7,20^ for the detection of CO_2_,^21−23^ humidity,^20,24,25^ NH_3_,^26^ NO_2_,^27^ SO_2_,^28^ volatile organic compounds,^20,29^ and chemical warfare agents.^30^

“Conventional” materials for sensitive layers of chemiresistors are oxides, where the concentration of free electrons or holes is modulated by the molecules of the detected gas.^31^ In contrast, the charge transfer in PILs is mediated by molecular ions, whose potentialities to interact with the detected gas is much more diverse: there is, for example, electrostatic interaction or the formation of a hydrogen bond. Moreover, the molecular ion in PIL can contain a receptor specifically bonding to a given target molecule, or at least a functional group that has an affinity for the target molecule.^17,32^ Studies have shown that charge transfer and polarizability in ionic liquids significantly influence their interactions with various molecules.^33^

We have recently demonstrated the chemiresistive properties of P_4,4,4,6_SPA, a combination of the tributylhexyl phosphonium cation and the sulfopropyl acrylate polyanion, towards 10 ppm of toxic gases (nitrogen dioxide, methanol, 4-bromoacetophenone, and diethylmalonate). Our findings indicate that the sensor dynamics (i.e., response and recovery times) are influenced by the molecular weight of the analyte as well as the number of reactive centers in its molecule.^30^ However, little is known, in general, about the gas-sensing mechanisms of PILs and their interactions with analytes containing different functional groups.

In order to gain further information, we extend here our investigation of the interactions between P_4,4,4,4_SPA or P_4,4,4,8_SPA (i.e., tetrabutylphosphonium sulfopropyl acrylate and tributyloctyl phosphonium sulfopropyl acrylate) and five different gaseous analytes, each containing two-carbon atoms with different functional groups, using a combination of EIS (electrochemical impedance spectroscopy) and structural models of PILs in solution. Two variations of PILs (P_4,4,4,4_SPA (**1**), and P_4,4,4,8_SPA (**2**)) with different chain lengths of the alkylphosphonium cation were synthesized from the respective ILs and subsequently characterized by UV–vis and Raman spectroscopy. The gaseous analytes—acetaldehyde, acetic acid, acetonitrile, bromoethane, and ethanol—were chosen as representatives due to their similar molecular size, but variable dipole moment, polarity, electric permittivity, ability to form hydrogen bonds and Bronsted acidity. Relatively high concentrations of 10,000 ppm for the analytes were chosen to reveal their sensing mechanisms.

Furthermore, we studied and compared the properties of pure PILs **1** and **2** with those of their fullerene-doped composites: P_4,4,4,4_SPA + C_60_ (**1** + C_60_), P_4,4,4,4_SPA + C_70_ (**1** + C_70_) and P_4,4,4,8_SPA + C_60_ (**2** + C_60_), P_4,4,4,8_SPA + C_70_ (**2** + C_70_). It has been reported that the addition of carbon materials to composites, in general, leads to higher sensitivity toward low gas concentrations and enhances greater gas response selectivity.^34^ To the best of our knowledge, no work has investigated the use of C_60_ or C_70_ composites with PILs in sensitive layers of chemiresistors.^35^

## Results and Discussion

**Scheme 1:**
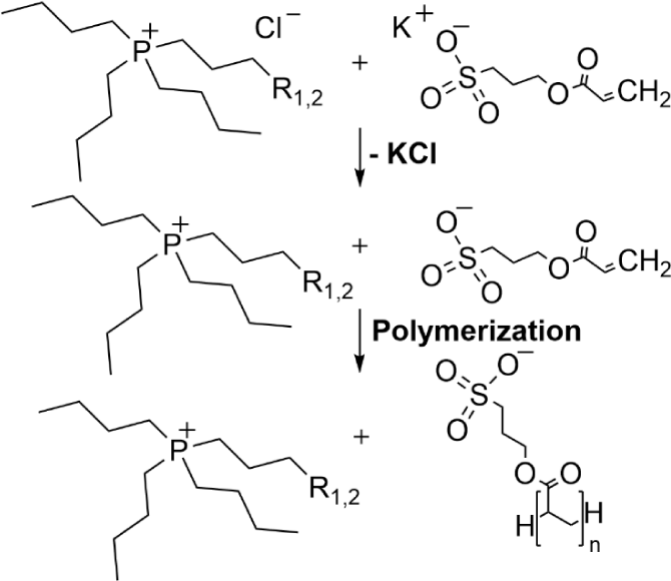
Synthesis of PIL Samples Using Tetrabutylphosphonium Chloride (P_4,4,4,4_Cl) and Tributyloctyl-Phosphonium Chloride (P_4,4,4,8_Cl) in Combination with Potassium 3-(Acryloyloxy)Propane-1-Sulfonate (KSPA) with R_1_=CH_3_ Corresponding to P_4,4,4,4_SPA (**1**) and R_2_=C_5_H_11_ Corresponding to P_4,4,4,8_SPA (**2**)

**Scheme 2:**
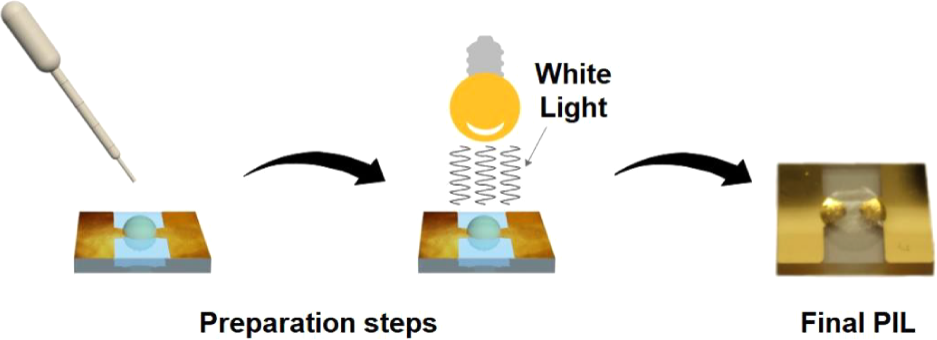
Preparation of PILs of Gold Electrode Substrate

Six PIL containing samples are based on the sulfopropyl acrylate polyanion (SPA) in combination with the alkylphosphonium cations, P_4,4,4,4_ (**1**) and P_4,4,4,8_ (**2**), as well as their fullerene-nanocomposites, P_4,4,4,4_SPA + C_60_ (**1** + C_60_), P_4,4,4,4_SPA + C_70_ (**1** + C_70_) and P_4,4,4,8_SPA + C_60_ (**2** + C_60_), P_4,4,4,8_SPA + C_70_ (**2** + C_70_). The preparation is outlined here (for full synthetic details, see S1 - S3):

(i) P_4,4,4,4_SPA (**1**) and P_4,4,4,8_SPA (**2**), the ILs “monomers”, were synthesized according to Scheme 1: The phosphonium salts were prepared from tetra-*n*-butyl-phosphonium chloride (P_4,4,4,4_Cl) and tributyl-octyl-phosphonium chloride (P_4,4,4,8_Cl), respectively, using an ion exchange method.
(ii) Both ILs were modified by the addition of C_60_ and C_70_, respectively, leading to **1** + C_60_, **1** + C_70_ and **2** + C_60_, **2** + C_70_. These composites were (after polymerization) compared with the pure PILs.
(iii) The synthesized ILs were applied in the form of a single drop (1.5 μL) to the area between the gold electrodes on the prepared substrates (Scheme 2). For substrate preparation (see Figure S1), the ILs were immediately polymerized by exposure to white light for 45 min.

The resulting PILs formed a colorless bead with the typical polymer gel consistency, constrained at the defined position. All samples were subsequently characterized by using UV–vis and Raman spectroscopy to determine the fullerene content. Electrochemical impedance spectroscopy measurements were utilized to analyze the interactions between the prepared PIL samples and the five different gaseous molecules: acetaldehyde, acetic acid, acetonitrile, bromoethane, and ethanol.

## Optical Spectroscopy

### Raman Spectra of Prepared PILs With/Without C_60_

The observed Raman bands for both PIL samples, **1** and **2**, are summarized and assigned in Table S2. The Raman spectra of both pure PILs exhibit similar features appearing at similar positions. There are two clear differences between the two recorded spectra, appearing at 2733 and 1414 cm^–1^ in the spectra of P_4,4,4,8_ (**2**). This is probably due to the extended length of one alkyl chain in **2**. Most of the Raman bands of phosphonium PILs correspond to deformations and stretching vibrations of chemical bonds of carbon atoms with hydrogen, oxygen, or phosphorus. The most intense Raman peaks arise from nonpolar functional groups, because of a stronger change of dipole moments and their polarizabilities.^36^ The Raman spectra of **1** and **2** are included in Figures S2–S5.

The Raman spectrum of P_4,4,4,4_SPA + C_60_ (**1** + C_60_) is shown in Figures S6 and S7 as an example of the composite materials investigated in this study. The addition of fullerene to **1** did not result in spectral changes that could be assigned to C_60_ and thus this spectrum rather resembles that of the pristine PILs. This is most likely due to the low intensity of the Raman bands for C_60_ in contrast to the intense Raman bands of nonpolar functional groups of **1**.

### UV–vis Spectra of Prepared PILs With/Without C_60_

The UV–vis spectrum of P_4,4,4,4_SPA (**1**) shows the main absorbance between 200 and 300 nm and displays four main peaks, at 224 nm, two close peaks at 237 and 247 nm, and a broad shoulder peak at 260 nm. The UV–vis spectra of **1** and **2** together with their nanocomposites, **1** + C_60_ and **2** + C_60_, are included in Figures S9–S12.

With P_4,4,4,8_SPA (**2**) having only a longer alkyl chain, its UV–vis spectrum is expectedly very similar to that of **1**. The three central intense absorption bands (224, 237, and 247 nm) are present in both samples (Figures S9 and S10) and only vary in their intensities. The assigned band at 210 nm present in **2** is only visible in the form of a shoulder in the spectrum of **1**, but at the same position. A similar trend can be observed for the assigned band at 260 nm in the spectrum of **1**, which is then only visible as a shoulder in the spectrum of **2**.

The UV–vis spectrum of the drop-casted 35 μM solution of C_60_, (dissolved in methylcyclohexane) exhibits three broad absorption bands with maxima at 217, 265, and 333 nm (see Figure S13) which is in good agreement with the spectra found in the literature.^37,38^ For pure C_70_, four intense broad absorption bands with maxima at 330, 359, 377, and 468 nm can be observed.^39^

However, the absorbance bands in both spectra are low, which is most likely due to the low amount of deposited fullerenes. The low contributions of fullerenes usually observed in the spectra will therefore only have a small impact on the UV–vis spectra of the composite PIL materials.

As expected, the UV–vis spectrum of the composite material **1** + C_60_ (Figure S11) does not show any additional bands originating from C_60_ and rather resembles the spectrum of **1**. This phenomenon can be caused by the low concentration of C_60_ present in the nanocomposite and/or by some intermolecular interactions between the fullerene C_60_ molecules and P_4,4,4,4_SPA. A blueshift is observed for the two peaks present in **1** (247 and 260 nm) to lower wavelengths (241 and 252 nm), which is due to the presence of the fullerene.

The UV–vis spectrum of the nanocomposite **2** + C_60_ (Figure S12) has four main peaks near 210 nm, 224 nm,

249 and 262 nm with slightly different intensities than **2** itself. A similar blueshift of the lower energy peaks is observed upon the addition of C_60_ to P_4,4,4,8_SPA and, conversely, the two bands at 237 and 247 nm observed in the spectrum of **2**, shift to higher wavelengths (249 and 262 nm).

### Electrochemical Impedance Spectroscopy (EIS)

The gas responses towards acetaldehyde, acetonitrile, bromoethane, ethanol, and acetic acid of the prepared sensing layers consisting of

(i) “bare” P_4,4,4,4_SPA (**1**) and P_4,4,4,8_SPA (**2**), respectively and
(ii) their nanocomposites with fullerenes (**1** + C_60_, **1** + C_70_ and **2** + C_60_, **2** + C_70_)

were investigated using electrochemical impedance spectroscopy (EIS) to avoid the various drawbacks of DC measurements (irreversible or slowly reversible changes in the polymer structure, heating of the polymer).^40^ Performing a full impedance analysis allowed us to monitor “resistive” and “capacitive” changes simultaneously, thus increasing the selectivity of the sensors.^41^

The five chosen gas analytes for the PIL sensor measurements have sufficient vapor tension to prepare a sample containing a concentration of 10,000 ppm of the given vapor in synthetic air. These relatively high concentrations were chosen purposely to conduct a case study to reveal possible gas sensing mechanisms.

The measured impedance spectra of the PIL samples (**1** and **2**) and their nanocomposites (**1** + C_60_, **1** + C_70_ and **2** + C_60_, **2** + C_70_) were described using a Randles circuit with a CPE model (Figure S14). Three impedance spectra were recorded for each sample and each analyte. The details concerning the sequence of measurements can be found in the Experimental Section. Figure 1 exemplarily shows the results of **1** + C_70_ upon acetaldehyde exposure. All other EIS results are shown in Figures S14–S19 for acetaldehyde, Figures S20–S25 for acetic acid, Figures S26–S31 for acetonitrile, Figures S32–S37 for bromoethane, and Figures S38–S43 for ethanol.

**Figure 1:**
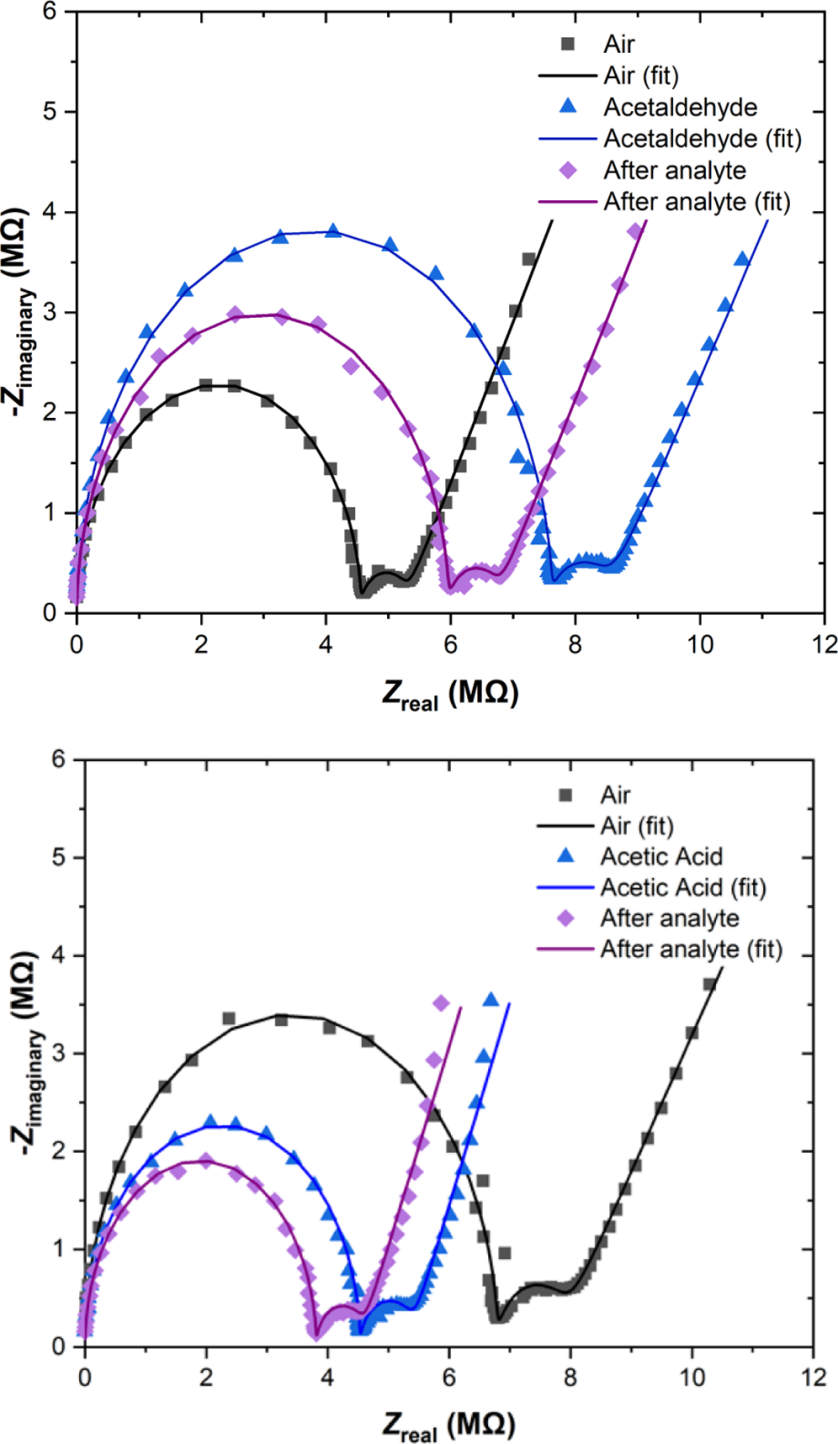
Measured sensor response of P_4,4,4,4_SPA + C_70_ (**1** + C_70_) to synthetic air (black square), on exposure to acetaldehyde (top) and acetic acid (bottom) (blue triangle), and to synthetic air after exposure (purple diamond) and their fits as solid lines.

The exponents α were found to be in the range of 0.49–0.79 upon exposure to the analytes, and in the majority of samples, they deviated from the expected Warburg value of 0.5. This implies that the Warburg impedance could not be used directly for all of the samples, while the CPE model better describes the obtained data.

The relative changes, Δ*Y*_0_, of all PIL materials when exposed to the analyte, are summarized in Table 1 and show the relative change of *Y*_0_, as Δ*Y*_0_ = *Y*_0_ (reference gas/synthetic air) – *Y*_0_ (analyte). Using the relative values of Δ*Y*_0_ will highlight the different behavior of the PIL materials exposed to each analyte and will allow us to investigate trends and correlations which can arise by taking the relative values rather than the absolute ones into account. The absolute values of *Y*_0_ before and after exposure to the analyte together with their difference, which was used to calculate the relative changes Δ*Y*_0_ are summarized in Table S3. A decrease in the *Y*_0_ coefficient, a negative value of Δ*Y*_0_, indicates a lower mobility of the phosphonium cations.

**Table 1:** Relative Changes, Δ*Y*_0_, of all PIL Materials when Exposed to the Analyte (with Δ*Y*_0_ (%) = [(*Y*_0_ (Synthetic Air) – *Y*_0_ (Analyte)) × 100]/*Y*_0_ (Synthetic Air); the Δ*Y*_0_ Values are given in Relation to the Reference *Y*_0_ Value in Synthetic Air)

Δ*Y*_0_ (%) = [(*Y*_0_ (synthetic air) – *Y*_0_ (analyte)) × 100]/*Y*_0_ (synthetic air)	1	1 + C_60_	1 + C_70_	2	2 + C_60_	2 + C_70_
Acetaldehyde	–10.79	–9.90	–15.71	–6.18	–10.20	–9.38
Acetic Acid	30.43	31.37	40.94	39.39	38.27	52.52
Acetonitrile	1.44	4.27	1.92	8.88	–2.79	–0.39
Bromoethane	–25.39	–26.96	–29.35	–30.80	–21.98	–30.79
Ethanol	–8.04	–9.66	–10.82	–9.23	–12.05	–15.69

The EIS results (Table S3) show a clear decrease for all six PIL samples (**1**, **1** + C_60_, **1** + C_70_ and **2**, **2** + C_60_, **2** + C_70_) in the *Y*_0_ value parameter upon exposure to acetaldehyde, bromoethane, and ethanol, indicating a decrease in the diffusion coefficient of the phosphonium cations and therefore a lower mobility. There is a clear inverse relationship between the resistance *R*_ct_ and *Y*_0_ in the CPE circuit parameter values. This behavior was expected since phosphonium cations are the main charge carriers in the PILs and lowering their mobility will result in an increase of resistance.

Comparing the responses of all PIL materials (see Table 1), it is clear that the largest increase in *Y*_0_ values (positive Δ*Y*_0_) is observed upon exposure to acetic acid, while the largest decrease in *Y*_0_ values (negative Δ*Y*_0_) is found for bromoethane. All investigated PIL materials show the weakest response upon exposure to acetonitrile where both small positive and small negative values are observed.

Overall, the addition of C_70_, especially **2** + C_70_, shows the highest response for all measured analytes, with only one exception in the response for acetaldehyde, where **1** + C_70_ exhibits the highest response.

A comparison of the Δ*Y*_0_ absolute values of “bare” PILs (samples **1** and **2**) with that of PIL-fullerene nanocomposites (samples **1** + C_60_, **1** + C_70,_**2** + C_60_, **2** + C_70_) (Table S3) reveals that the response of “bare” PILs is enhanced (the sign of percentual change is equivalent with that of Δ*Y*_0_) when C_60_ or C_70_ is added.

Here, the following trends can be observed: (i) the addition of C_70_ enhanced the response for both PILs and all analytes; (ii) of the measured analytes, the response to ethanol and acetonitrile was always stimulated by the addition of fullerenes; however, in the case of acetonitrile, the Δ*Y*_0_ magnitude is low, so there is a low basis for comparison. It is important to note that acetonitrile is hardly representative of an aprotic solvent, which could potentially influence the observed response. For ethanol, a percentage change of 20.0–30.6% is observed when adding C_60_ and 34.6–69.9% when adding C_70_, respectively.

Interestingly, the sensor responses to acetic acid exposure exhibit a unique behavior that was not observed for any other investigated gaseous analyte, showing a significant and nonreversible increase in the *Y*_0_ value (Figures 1 and S21–S26). This phenomenon is most likely caused by the adsorption and dissociation of the acetic acid onto the sensing layer, creating two mobile ions, the acetate anion and the proton or oxonium cation. Both, the oxonium cations and acetate anions could have contributed significantly to the increase in the *Y*_0_ value because of their high diffusion coefficients.^36,37^ Furthermore, the dissociated acetic acid can undergo an anion exchange with the respective PILs, leading to the formation of [P_4,4,4,4_][OAc] or [P_4,4,4,8_][OAc], respectively, which are the preferred products according to the HSAB principle.

**Figure 2:**
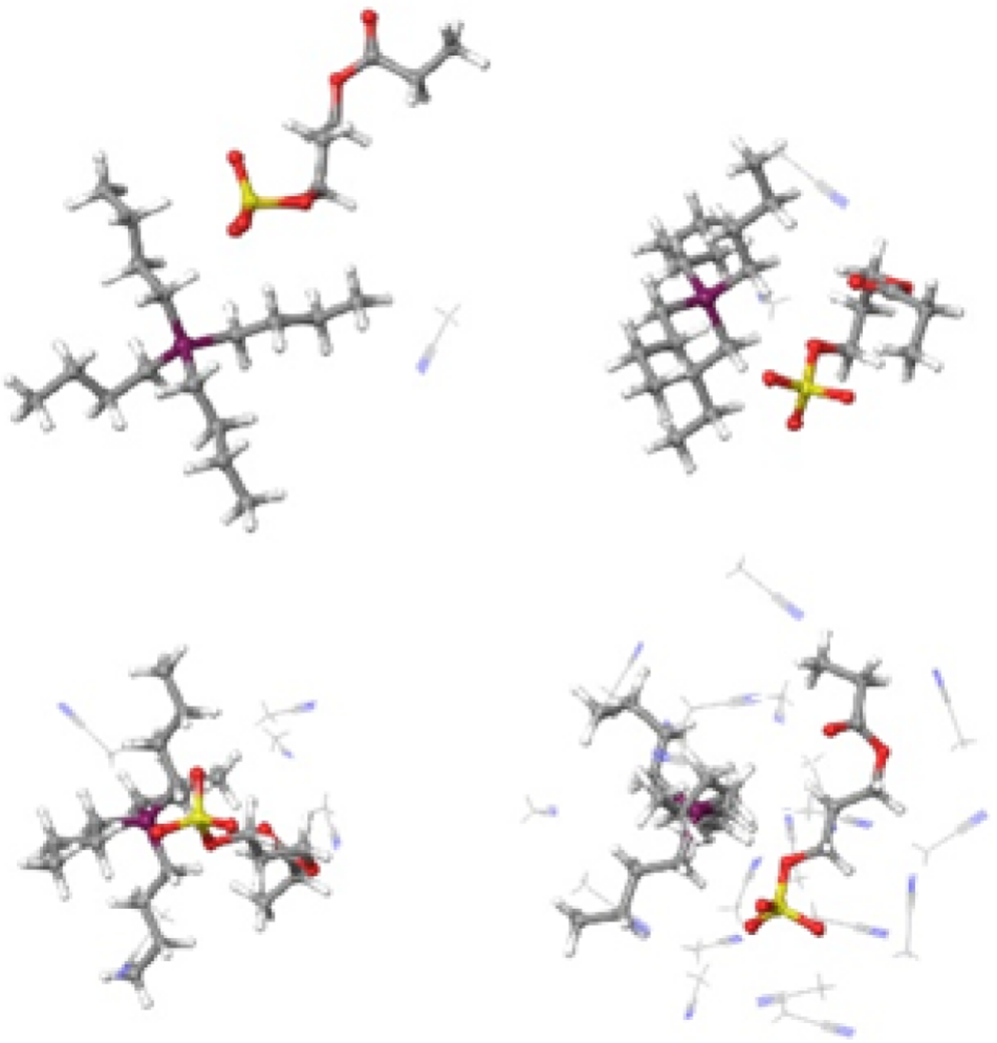
Interactions of P_4,4,4,4_-SPA upon sequential addition of explicit acetonitrile solvent molecules (1 (top left), 2 (top right), 5 (bottom left), 20 (bottom right)). The phosphonium-polymer (P–S) distances of 4.75, 4.52, 5.02, and 4.18 Å, respectively, are almost unchanged from that of the isolated complex (4.75 Å).

### Summary of Responses

Δ*Y*_0_ of the six PIL materials expectedly shows a similar value with the same order of magnitude when exposed to the same analyte. The only exception to this trend is found using acetonitrile, where relatively small Δ*Y*_0_ values, both positive and negative, were observed. However, when exposing different analytes to the same PIL composite, both an increase in *Y*_0_ (positive Δ*Y*_0_ for acetic acid and acetonitrile) and a decrease in *Y*_0_ (negative Δ*Y*_0_ for acetaldehyde, bromoethane, and ethanol) can be observed.

We tried to find a correlation between the Δ*Y*_0_ values, and the physical properties of the measured analytes (relative permittivity *ε*_*r*_, dipole moment μ and relative polarity , see Table S14 and Figures S45–47) to accelerate and improve the search for new analytes with high response signals. Acetic acid was excluded from the linear regression of all physical properties due to its p*K*_a_ value, possible deprotonation into carboxylate anions, and subsequent rearrangement of the PILs. A reasonable correlation can be found for all of the properties of analytes. In order to increase the number of data points, and to obtain a better correlation, we have tried to incorporate the possibility to compare the data shown herein with our previously published results based on P_4,4,4,6_SPA toward 10 ppm of toxic gases: nitrogen dioxide (NO_2_), methanol (MeOH), 4-bromoacetophenone (4-BAP), diethylmalonate (DEM)) using DC resistivity.^30^ Here, the highest direct current response and phase angle sensitivity were found for 4-BAP, followed by MeOH and DEM, while NO_2_ exhibited the lowest response.^30^ This sequence quantitatively also correlates with the dipole moments (μ) of the analyte molecules: 4-BAP μ = 3.1D; MeOH μ = 1.7D; DEM μ = 2.54D; and NO_2_ μ = 0.6D.

Incorporating the findings from both studies reveals, that there is no clear trend when the resistance change (Δ*R*) is compared to the dipole moments of all eight analytes (Figure S48).

Therefore, we suggest that the sensing mechanism of the studied PILs and their composites with C_60_ and C_70_ toward gaseous analytes is not only related to one of these unique properties, but there are additional influences on a molecular scale, such as coordinating effects due to hydrogen bonding, free electron pairs, or other intermolecular interactions. In order to shed light on the sensing mechanism and to model intermolecular interactions, we performed highly efficient semiempirical quantum mechanical xTB-GFN2 calculations.

### Computational Modeling of Microsolvation

In order to investigate the coordinative properties of different analytes with the used PIL samples, computational conformational searches were performed for microsolvated clusters of PILs. Ethanol, and undissociated acetic acid were chosen as hydrogen bond-donating solvents, acetonitrile was chosen as a potential hydrogen bond acceptor, whereas dissociated acetate anion, AcO^–^, and protonated SPA were models to describe the observed increase in mobility (see above). The binding free energy of SPA to the phosphonium cation in the 1:1 complex P_4,4,4,4_SPA is −97.0 kcal/mol at an intermolecular distance of 4.75 Å (P–S distance, due to the steric demand of cation and anion) compared to 4.9 Å (between P–C) and −105.5 kcal/mol for acetate. This shows that Coulombic interactions are critical in the PIL systems plus small molecule solvents, but they cannot solely explain the experimentally observed differences in changes in *Y*_0_

The largest differences in the *Y*_0_ coefficients were observed upon the response of PILs to acetonitrile (ε_*r*_ = 2.18) and acetic acid (ε_*r*_ = 38.94). Therefore, the effects of those solvents on the solute–solute interactions for these two representative sets were investigated in more detail. Acetonitrile has a medium polarity, medium hydrogen bonding ability, and is nonacidic (p*Ka* ∼ 25 in water). Upon adding an increasing number of acetonitrile solvent molecules, the P_4,4,4,4_SPA interaction is persistent, and the P–S distance (with 4.75, 5.02, 4.55, and 4.18 Å, respectively) is almost unaffected by the presence of 1, 2, 5, or 20 solvent molecules (Figure 2).

Ethanol, on the other hand, is a polar (ε_*r*_ = 24.3) and nonacidic solvent (p*Ka* ∼ 16) with exquisite propensities to donate and accept hydrogen bonds. Upon explicitly considering and increasing the number of ethanol molecules (1, 2, 5, 10, 20), the solvent molecules form hydrogen bonds with the sulfonate and the ester groups of SPA (see Figure S49). In larger cluster models with 10 and 20 ethanol molecules, additional hydrogen bonding among solvent molecules can be seen to make up a first microsolvation shell (typical hydrogen bond distances 1.94–1.97 Å and angles between 173 and 178°; Figure S49). However, the P_4,4,4,4_SPA solute–solute interaction and P–S distance are unaffected (with distances between 4.58 and 4.75 Å). Three ethanol solvent molecules form hydrogen bonds with the sulfonate group of SPA (at distances of 1.90–1.93 Å).

Undissociated, protonated acetic acid behaves similarly to other investigated analytes (data not shown). The formation of hydrogen bonds with the sulfonate (at 1.83, 1.94, and 1.94 Å and 173–178°) and ester group oxygen atoms does not perturb the P–S interaction.

**Figure 3:**
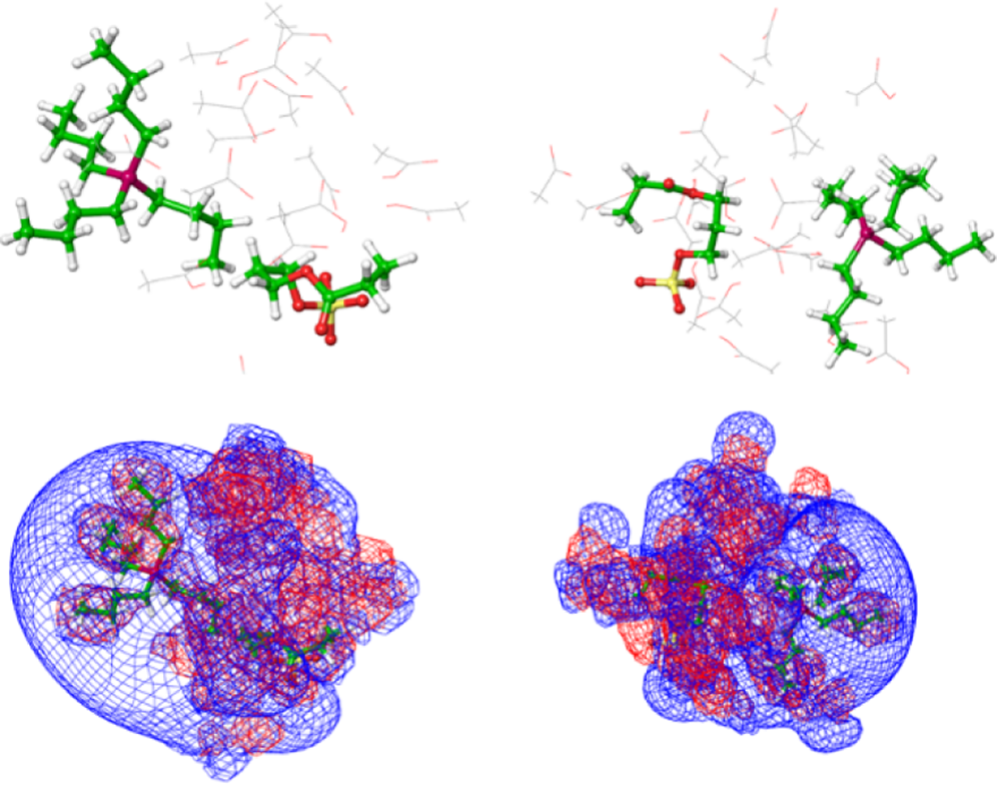
Top: representative structures of P_4,4,4,4_SPA in the presence of 8/8 (left) and 20/20 (right) (acetic acid/acetate) solvent molecules. The strong electrostatic interaction is perturbed, and the P^+^-S^–^ distances significantly increase to 9.3 and 9.2 Å, respectively. Bottom: mesh plot of the electrostatic potential at a contour level of ±1 kT (blue is positive, red is negative). Some acetic acid/acetate solvent molecules are located between the solute molecules.

Only when taking into account the partial dissociation of acetic acid and a full first solvation shell of 8/8 and 20/20 (acetic acid/acetate) molecules is considered, an effect on the solute–solute interaction can be seen (Figure 3). The undissociated acetic acid molecules form hydrogen bonds with the ester oxygen atoms (at 1.96 Å) and the sulfonate group (as above at 1.7–1.8 Å) only have a minor effect on the solute–solute interaction. Hydrogen bonds between acetic acid molecules (1.8–1.9 Å) and acetate and acetic acid (1.5 Å) are formed. The angle between hydrogen bond donor and acceptor is close to 180°. However, negatively charged acetate ions are able to, at least partially, approach the P_4,4,4,4_ phosphonium cation, displace SPA, and thus induce larger P–S distances (for example, to 9.3 Å for 8/8 and 9.2 Å for 20/20 solvent molecules). Some of the acetate anions even occupy the space between the P^+^ and S^–^ (typically at 5 and 4 Å), and therefore significantly perturb the PIL interaction, thus enabling more facile P_4,4,4,4_ diffusion (Figure 3). Therefore, we suggest that the sensing mechanism of the studied PILs and their composites with C_60_ and C_70_ toward gaseous analytes is not only related to one of these unique properties, but there are additional influences on a molecular scale, such as coordinating effects due to hydrogen bonding, free electron pairs, or other intermolecular interactions. In order to shed light on the sensing mechanism and model intermolecular interactions, we performed highly efficient semiempirical-quantum mechanical xTB-GFN2 calculations.

## Conclusions

In this work, we have synthesized and characterized six PIL samples (**1**, **1** + C_60_, **1** + C_70_ and **2**, **2** + C_60_, **2** + C_70_) and investigated them as the active material of chemical gas sensors (towards acetaldehyde, acetic acid, acetonitrile, bromoethane, and ethanol) and have found the following:

(i) The largest response, with a significant positive Δ*Y*_0_, was recorded for the exposure to acetic acid, indicating not a “true” sensing mechanism, but an absorption and transformation mechanism, leading to the formation of [P_4,4,4,4_][OAc], i.e., a change in sensitive layer composition, which was supported by theoretical calculations.
(ii) Bromoethane showed the second largest response, with a negative change in its *Y*_0_ value. Acetaldehyde and ethanol had very similar magnitudes of response, while acetonitrile showed the lowest response with both small positive and small negative values observed.
(iii) In general, enhanced responses to all analytes were observed for both composites containing C_60_ and C_70._ Of the measured analytes, the response to ethanol and acetonitrile was always stimulated by the addition of fullerene (either C_60_ or C_70_).

Computational conformational searches were performed for microsolvated clusters of PILs using ethanol as a hydrogen bond-donating solvent and acetonitrile as a potential hydrogen bond acceptor. Ethanol microsolvation leads to the formation of hydrogen bonds with the SPA moieties. However, the P_4,4,4,4_SPA solute–solute interaction and the P–S distance are unaffected. When adding molecules of acetonitrile to the PIL, the P_4,4,4,4_SPA interaction persists, and the P–S distance is almost unaffected.

While undissociated acetic acid behaves similarly to ethanol, the negatively charged deprotonated acetate ions are, on the other hand, able to at least partially approach the P_4,4,4,4_ phosphonium cation and thus induce larger P–S distances, which supports the experimental findings of a significant increase in the value of *Y*_0_.

The relatively high analyte concentrations of 10,000 ppm were intentionally chosen to ensure clear mechanistic insights and facilitate interpretation across all characterization methods. While these high concentrations allowed us to study the gas–PIL interactions effectively, they are not directly representative of practical, real-world conditions. Future work will focus on determining calibration curves at lower concentrations to establish practical limits of detection (LOD) for the suitable analytes, which were determined in this study. Recent studies on other PIL-based sensors using EIS indicate the potential for low-concentration detection with LODs reported as low as 0.02 ppm for ammonia (NH_3_),^42^ 0.76 ppm for ethylene,^43^ 9 ppm for oxygen (O_2_),^44^ and 0.1 ppm for nitrogen dioxide (NO_2_).^45^

Our study shows that ethanol, acetaldehyde, and bromoethane produced pronounced responses; on the other hand, acetonitrile displayed only a weak response, and acetic acid induced irreversible changes in the sensitive layer. Therefore, the determination of the LOD would be relevant primarily for ethanol, acetaldehyde, and bromoethane.

To optimize the analyte detection at lower concentrations, we will attempt to use thinner layers of PIL composites while recording the chemiresistive response in DC mode:

(i) A thinner sensitive layer can help reduce the parallel resistance, thereby enhancing the sensor’s sensitivity and lowering the LOD. Using a smaller volume or different deposition method of the PIL material may achieve this improvement.
(ii) Operating chemiresistors in DC mode, which is connected in general with lower noise of sensor output. It is important to note that not all analytes in this study are suitable for LOD determination. Ethanol, acetaldehyde, and bromoethane demonstrated sufficient responses for potential LOD measurement, while acetonitrile displayed weak signals, and acetic acid induced irreversible changes in the sensitive layer.

In conclusion, this investigation of the six PIL samples (**1**, **1** + C_60_, **1** + C_70_ and **2**, **2** + C_60_, **2** + C_70_) revealed that PIL materials are not suitable candidates to be used as sensing media towards carboxylate functional groups (−COOH) due to the observed absorption and dissociation of carboxylic acids, which induces the transformation of the sensitive layer. On the other hand, they exhibit applicable potential in the chemical gas sensor field, in particular for sensing VOCs (volatile organic compounds) containing functional groups of alcohol (−OH), nitrile (−CN), aldehyde (−CHO), or halogenated hydrocarbons.

## Methods

### Materials and Synthetic Procedure

All chemicals and solvents (unless otherwise mentioned) were purchased from chemical companies, PENTA and Sigma-Aldrich, and were reagent grade. They were used without further purification or drying. All reactions were carried out under ambient conditions. Full synthetic details for all PIL samples (**1**, **1** + C_60_, **1** + C_70_, and **2**, **2** + C_60_, **2** + C_70_) can be found S1 - S3.

### Optical Spectroscopy

All samples, deposited on B270 optical glass substrates, were characterized using a Renishaw InVia Raman spectrometer with a laser excitation wavelength of 488 nm. Baseline correction, smoothing, and peak identification were applied to the spectra using the OMNIC 9.2.86 software.

The UV–vis spectra of the same samples were recorded in a transmission dark chamber using an Ocean Optics HR 2000+ spectrometer, equipped with a halogen-deuterium lamp Ocean Optics DT-MINI-2GS (wavelength range of 215–2500 nm) and Ocean Optics 400 μm optical fiber. Absorbance spectra of clean optical glass were used as a reference.

### Electrochemical Impedance Spectroscopy (EIS)

**Figure 4:**
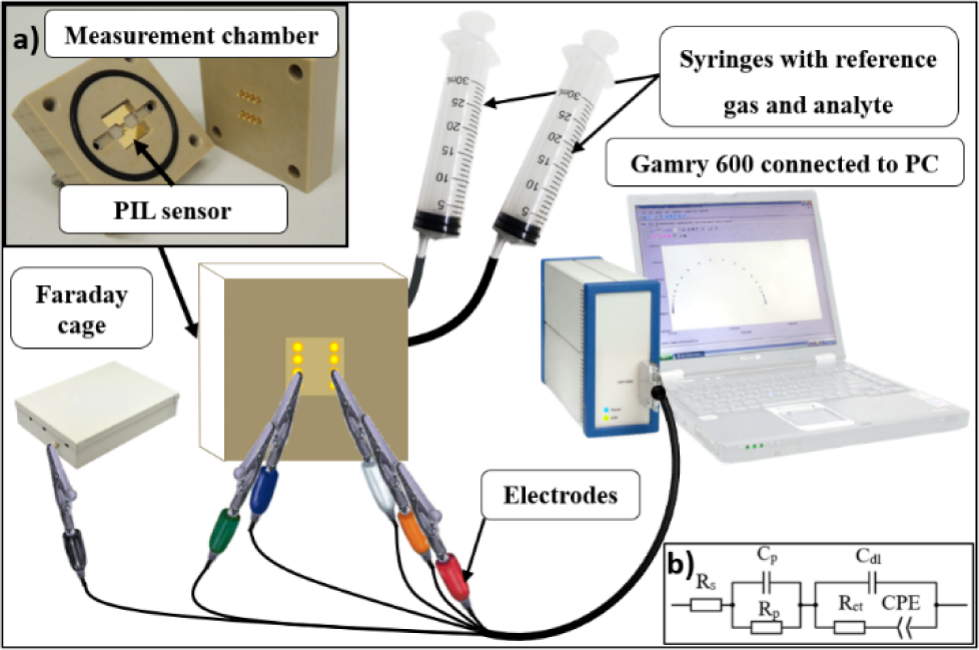
Scheme of gas measurement chamber and wiring; inset: (a) measurement chamber and (b) scheme of the Randles circuit with CPE.

The EIS measurements were performed in a Faraday cage shielding to avoid external noise (Figure 4) and the PIL sensor substrates were placed in an in-house-built measurement PEEK chamber with gold contacts (Figure 4a). This chamber was connected to the GAMRY Reference 600 potentiostat/galvanostat with the measuring software (GAMRY FRAMEWORK Version 6.21). Two syringes were attached to the chamber as gas inlet and outlet.

### Measurement Sequence

The response upon gas exposure of the six samples was measured according to the following sequence: (i) 3 × 60 mL of synthetic air was forced through the measurement chamber, and the impedance spectrum was measured after 15 min of stabilization; (ii) 3 × 60 mL of analyte with a concentration of 10,000 ppm was forced through the measurement chamber and the impedance spectrum was measured after 30 min; (iii) 3 × 60 mL of synthetic air was again forced through the measurement chamber, and after 15 min the impedance spectrum was measured to determine the recovery.

Impedance measurements were recorded at room temperature over a frequency range from 1 MHz to 0.1 Hz at a potential amplitude of 100 mV and a density of 10 measurement points per decade of frequency. Frequencies were applied gradually (single-sine technique). The Nyquist representation highlights, that in the high-frequency region of the measurements, there is no significant change that could be correlated with the presence of a gaseous analyte. This is mainly due to the relatively large dimensions and high mass of the alkylphosphonium cations, resulting in their low mobility at high frequencies. On the other hand, in the case of low-frequency (subhertz) measurements, the measurement can take a significant amount of time in order to characterize the dynamic sensor properties such as response time.

The Randles circuit with a constant phase element (CPE) was used as a model for fitting the measured EIS data (Figure 4b). It is possible to compare the diffusion coefficients of mobile ions in individual measurements by evaluating the value *Y*_0_ (eq 1), which is inversely proportional to the Warburg coefficient when α = 0.5.
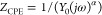
1

Therefore, *Y*_0_ is proportional to the diffusion coefficient () and the exponent α is related to the deviation from the Warburg impedance.

### Preparation of Gas Analytes

The following five analytes were investigated within this study: acetaldehyde (PENTA s.r.o., p.a.), acetic acid (PENTA s.r.o., p.a.), acetonitrile (PENTA s.r.o., p.a.), bromoethane (Sigma-Aldrich, 98.0%), and ethanol (PENTA s.r.o., p.a.).

Supel-Inert Multi-Layer Foil chromatographic bags were used to prepare the gaseous mixtures of the selected analytes with a concentration of 10,000 ppm in dry and “pure” synthetic air. The exact volume of each analyte was taken using a Hamilton syringe, which was also weighed and subsequently injected into the chromatographic bag to evaporate. All the laboratory experiments were conducted at a constant laboratory temperature of 22 °C.

### Computational Details

A model was set up to describe the intermolecular interactions in monomeric and poly(ionic liquids). The P_4,4,4,4_ phosphonium cation (R_1_ in Scheme 1) was chosen as a representative. Monomeric 3-sulfopropyl acrylate (SPA) with *n* = 1 (Scheme 1) is used to describe the polymer–phosphonium interactions.

Global optimum conformational searches using the semiempirical xTB-GFN2 Hamiltonian^46^ and the Conformer-Rotameter Sampling Tool^47^ (CREST) that consists of iterative meta-dynamics, genetic Z-matrix crossing, and equilibrium molecular dynamic simulations were performed in an ALPB representation of water as a solvent medium. The GFN2-xTB Hamiltonian is an extended tight-binding model, primarily designed for the fast calculation of structures and noncovalent interactions for large molecular systems. It does not require a reparametrization for different binding situations and only relies on global, element-specific parameters. CREST makes use of the GFN2-xTB method for efficient conformational sampling at the extended tight-binding level. It returns different low-energy conformations of noncovalently bound complexes, as in this example. Different molecular models for ionic liquid compositions of P_4,4,4,4_SPA in the presence of explicit solvent molecules—acetonitrile, ethanol, and acetic acid/acetate—were investigated in order to probe the effects of gas molecules on the PIL electrostatic stabilization. The solvent molecules were randomly positioned close to the solute, and the overall minimum arrangements were obtained after exhaustive CREST searches using the “noncovalent” NCI mode with a repulsive wall potential.

## Supporting Information Available

Synthesis of monomeric ionic liquids P_4,4,4,4_SPA and P_4,4,4,8_SPA; preparation of the solution of C_60_ and C_70_ fullerenes; polymerization of the ILs monomers; preparation of sensor substrates; Raman spectra of (**1**), (**2**), (**1**+C_60_) and C_60_ ; UV–vis spectra of pure (**1**) and (**2**), and (**1**+C_60_) and (**2**+C_60_) and C_60_; EIS measurement setup; measured data of all six sensor materials and their response to synthetic air, analyte exposure and after exposure for all five analytes including their evaluated parameters; dependence of Y_0_ to relative permittivity, dipole moment, relative polarity and sensor resistance; interactions of P_4,4,4,4_SPA (**1**) upon sequential addition of explicit ethanol solvent molecules; references within the Supporting Information.^30^^,^^48^^–^^52^(PDF)

## Abbreviations

P_4,4,4,4_SPA: tetrabutylphosphonium sulfopropyl acrylate
P_4,4,4,8_SPA: tributyloctyl phosphonium sulfopropyl acrylate
EIS: electrochemical impedance spectroscopy
UV–vis spectroscopy: ultraviolet–visible spectroscopy
DC: direct current
LOD: limit of detection
VOC: volatile organic compound
HSAB: hard and soft acids and bases
CPE: constant phase element
ALPB: analytical linearized Poisson–Boltzmann
xTB-GFN2: extended tight-binding method with geometry, frequency, and noncovalent corrections
