# Supplementary material for: Fullerene-Doped Poly(ionic liquids) as Small Molecular Gas Sensors—Control of Intermolecular Interactions
Source: ACS Omega. 2024 Dec 23;10(1):1364–72. doi: 10.1021/acsomega.4c08941 (PMC11740104; doi:10.1021/acsomega.4c08941)
Supplement: Supplementary file 1 — ao4c08941_si_001.pdf [file ao4c08941_si_001.pdf]

Supporting Information

Fullerene-Doped Poly(ionic Liquids) as Small Molecular Gas Sensors – Control of Intermolecular Interactions.

Jaroslav Otta,[a,b] Jakub Mikuláštík,[b] Richard Šípka,[b] Matthias Stein,[c] Irina A. Kühne,[a] Martin Vrňata,[b] and Jan Vlček[a,b,]*

[a] Department of Functional Materials, FZU - Institute of Physics - Czech Academy of Sciences, Na Slovance 1999/2, Prague 8, 182 21, Czech Republic.

[b] Department of Physics and Measurements, University of Chemistry and Technology, Technicka 5, 16 628 Prague 6, Czech Republic.

[c] Molecular Simulations and Design Group, Max Planck Institute for Dynamics of Complex Technical Systems, Sandtorstrasse 1, 39106 Magdeburg, Germany.

[a)] *Corresponding author: vlcekj@fzu.cz*

Table of Content

S1 Synthesis of monomeric ionic liquids $P_{4,4,4,4}$SPA and $P_{4,4,4,8}$SPA 5

S2 Preparation of the solution of C_{60} and C_{70} fullerenes 5

S3 Polymerization of the ILs monomers 5

Table S1. Polymerization mixture compositions. 5

S4 Preparation of sensor substrates by magnetron sputtering of gold electrodes 6

Figure S1. Preparation of gold electrodes on optical glass substrates via magnetron sputtering. 6

S5 Raman spectra of prepared PILs with/without C_{60} fullerene 7

Figure S2. Raman spectrum of $P_{4,4,4,4}$SPA **(1)** in range 500 – 3000 cm^{-1}. 7

Figure S3. Raman spectrum of $P_{4,4,4,4}$SPA **(1)** in range 500 – 1700 cm^{-1}. 7

Figure S4. Raman spectrum of $P_{4,4,4,8}$SPA (**2**) in range 500 – 3000 cm^{-1}. 8

Figure S5. Raman spectrum of $P_{4,4,4,8}$SPA (**2**) in range 500 – 1700 cm^{-1}. 8

Figure S6. Raman spectrum of $P_{4,4,4,4}$SPA + C_{60} (**1+C_{60}**) in range 500 – 3000 cm^{-1}. 9

Figure S7. Raman spectrum of $P_{4,4,4,4}$SPA + C_{60} (**1+C_{60}**) in range 500 – 1700 cm^{-1}. 9

Figure S8. Raman spectrum of drop casted C_{60} dissolved in methylcyclohexane in the range between 200 – 1700 cm^{-1}. ..10

Table S2. Representative Raman bands for $P_{4,4,4,4}$SPA (**1**) and $P_{4,4,4,8}$SPA (**2**) in the range between 3100 – 500 cm^{-1}. 10

S6 UV-Vis spectra of prepared PILs with/without C_{60} fullerene 11

Figure S9. UV-vis absorbance spectrum of pure $P_{4,4,4,4}$SPA (**1**) in the range between 200 - 700 nm. 11

Figure S10. UV-Vis spectrum of pure $P_{4,4,4,8}$SPA (**2**) in the range between 200 - 700 nm. 11

Figure S11. UV-VIS spectrum of nanocomposite $P_{4,4,4,4}$SPA + C_{60} (**1+C_{60}**) in the range between 200 - 700 nm. 12

Figure S12. UV-Vis spectrum of nanocomposite $P_{4,4,4,8}$SPA + C_{60} (**2+C_{60}**) in the range between 200 - 700 nm. 12

Figure S13. UV-Vis spectrum of drop casted C_{60} dissolved in methylcyclohexane in the range between 200 - 700 nm. 13

S7 EIS measurement and CPE model 13

Figure S14. **a)** Equivalent circuit model used for the analysis of the poly(ionic liquid) sensor response. **b)** The equation represents the impedance of the CPE, where $\boldsymbol{Y_0}$ is the admittance, $\boldsymbol{\alpha}$ is the phase angle exponent. 13

Table S3. Top: Absolute values of Y_0 before and after analyte exposure, together with their difference (*with* $\boldsymbol{Y_0} \cdot 10^{-9}$ in S s^{α}); Bottom: Relative changes, ΔY_0, of all PIL materials when exposed to the analyte (with $\Delta Y_0 = Y_0$ (reference gas/synthetic air) – Y_0 (analyte); the ΔY_0 values are given in relation to the reference Y_0 value in synthetic air), including the percentual change upon addition of fullerenes. 14

Table S4. Parameters evaluated using CPE model for acetaldehyde responses of sensors based on $P_{4,4,4,4}$SPA (**1**) and $P_{4,4,4,4}$SPA C_{60}/C_{70} (**1+C_{60}**, **1+C_{70}**) nanocomposites 15

Figure S15. Measured data of $P_{4,4,4,4}$SPA (**1**) sensor response to synthetic air (green), acetaldehyde (orange) and after exposure (blue). 15

Figure S16. Measured data of $P_{4,4,4,4}$SPA + C_{60} (**1+C_{60}**) sensor response to synthetic air (green), acetaldehyde (orange) and after exposure (blue). 16

Figure S17. Measured data of $P_{4,4,4,4}$SPA + C_{70} (**1+C_{70}**) sensor response to synthetic air (green), acetaldehyde (orange) and after exposure (blue). 17

Table S5. Parameters evaluated using CPE model for acetaldehyde responses of sensors based on $P_{4,4,4,8}$SPA (**2**) and $P_{4,4,4,8}$SPA C_{60}/C_{70} (**2+C_{60}**, **2+C_{70}**) nanocomposites 17

Figure S18. Measured data of $P_{4,4,4,8}$SPA (**2**) sensor response to synthetic air (green), acetaldehyde (orange) and after exposure (blue). 18

Figure S19. Measured data of $P_{4,4,4,8}$SPA + C_{60} (**2+C_{60}**) sensor response to synthetic air (green), acetaldehyde (orange) and after exposure (blue). 19

Figure S20. Measured data of $P_{4,4,4,8}$SPA + C_{70} (**2+C_{70}**) sensor response to synthetic air (green), acetaldehyde (orange) and after exposure (blue). 19

Table S6. Parameters evaluated using CPE model for acetic acid responses of sensors based on $P_{4,4,4,4}$SPA (**1**) and $P_{4,4,4,4}$SPA C_{60}/C_{70} (**1+C_{60}**, **1+C_{70}**) nanocomposites 19

Figure S21. Measured data of $P_{4,4,4,4}$SPA (**1**) sensor response to synthetic air (green), acetic acid (orange) and after exposure (blue). 20

Figure S22. Measured data of $P_{4,4,4,4}$SPA + C_{60} (**1+C_{60}**) sensor response to synthetic air (green), acetic acid (orange) and after exposure (blue). 21

Figure S23. Measured data of $P_{4,4,4,4}$SPA + C_{70} (**1+C_{70}**) sensor response to synthetic air (green), acetic acid (orange) and after exposure (blue). 21

Table S7. Parameters evaluated using CPE model for acetic acid responses of sensors based on $P_{4,4,4,8}$SPA (**2**) and $P_{4,4,4,8}$SPA C_{60}/C_{70} (**2+C_{60}**, **2+C_{70}**) nanocomposites 21

Figure S24. Measured data of $P_{4,4,4,8}$SPA (**2**) sensor response to synthetic air (green), acetic acid (orange) and after exposure (blue). 22

Figure S25. Measured data of $P_{4,4,4,8}$SPA + C_{60} (**2+C_{60}**) sensor response to synthetic air (green), acetic acid (orange) and after exposure (blue). 23

Figure S26. Measured data of $P_{4,4,4,8}$SPA + C_{70} (**2+C_{70}**) sensor response to synthetic air (green), acetic acid (orange) and after exposure (blue). 23

Table S8. Parameters evaluated using CPE model for acetonitrile responses of sensors based on $P_{4,4,4,4}$SPA (**1**) and $P_{4,4,4,4}$SPA C_{60}/C_{70} (**1+C_{60}**, **1+C_{70}**) nanocomposites 23

Figure S27. Measured data of $P_{4,4,4,4}$SPA (**1**) sensor response to synthetic air (green), acetonitrile (orange) and after exposure (blue)........24

Figure S28. Measured data of $P_{4,4,4,4}$SPA + C_{60} (**1+C_{60}**) sensor response to synthetic air (green), acetonitrile (orange) and after exposure (blue).........25

Figure S29. Measured data of $P_{4,4,4,4}$SPA + C_{70} (**1+C_{70}**) sensor response to synthetic air (green), acetonitrile (orange) and after exposure (blue).........25

Table S9. Parameters evaluated using CPE model for acetonitrile responses of sensors based on $P_{4,4,4,8}$SPA (**2**) and $P_{4,4,4,8}$SPA C_{60}/C_{70} (**2+C_{60}, 2+C_{70}**) nanocomposites.........25

Figure S30. Measured data of $P_{4,4,4,8}$SPA (**2**) sensor response to synthetic air (green), acetonitrile (orange) and after exposure (blue)........26

Figure S31. Measured data of $P_{4,4,4,8}$SPA + C_{60} (**2+C_{60}**) sensor response to synthetic air (green), acetonitrile (orange) and after exposure (blue).........27

Figure S32. Measured data of $P_{4,4,4,8}$SPA + C_{70} (**2+C_{70}**) sensor response to synthetic air (green), acetonitrile (orange) and after exposure (blue).........27

Table S10. Parameters evaluated using CPE model for bromoethane responses of sensors based on $P_{4,4,4,4}$SPA (**1**) and $P_{4,4,4,4}$SPA C_{60}/C_{70} (**1+C_{60}, 1+C_{70}**) nanocomposites.........27

Figure S33. Measured data of $P_{4,4,4,4}$SPA (**1**) sensor response to synthetic air (green), acetonitrile (orange) and after exposure (blue)........28

Figure S34. Measured data of $P_{4,4,4,4}$SPA + C_{60} (**1+C_{60}**) sensor response to synthetic air (green), bromoethane (orange) and after exposure (blue).........29

Figure S35. Measured data of $P_{4,4,4,4}$SPA + C_{70} (**1+C_{70}**) sensor response to synthetic air (green), bromoethane (orange) and after exposure (blue).........30

Table S11. Parameters evaluated using CPE model for bromoethane responses of sensors based on $P_{4,4,4,8}$SPA (**2**) and $P_{4,4,4,8}$SPA C_{60}/C_{70} (**2+C_{60}, 2+C_{70}**) nanocomposites........30

Figure S36. Measured data of $P_{4,4,4,8}$SPA (**2**) sensor response to synthetic air (green), bromoethane (orange) and after exposure (blue)........31

Figure S37. Measured data of $P_{4,4,4,8}$SPA + C_{60} (**2+C_{60}**) sensor response to synthetic air (green), bromoethane (orange) and after exposure (blue).........32

Figure S38. Measured data of $P_{4,4,4,8}$SPA + C_{70} (**2+C_{70}**) sensor response to synthetic air (green), bromoethane (orange) and after exposure (blue).........32

Table S12. Parameters evaluated using CPE model for ethanol responses of sensors based on $P_{4,4,4,4}$SPA (**1**) and $P_{4,4,4,4}$SPA C_{60}/C_{70} (**1+C_{60}, 1+C_{70}**) nanocomposites.........32

Figure S39. Measured data of $P_{4,4,4,4}$SPA (**1**) sensor response to synthetic air (green), ethanol (orange) and after exposure (blue). 33

Figure S40. Measured data of $P_{4,4,4,4}$SPA + C_{60} (**1+C_{60}**) sensor response to synthetic air (green), ethanol (orange) and after exposure (blue)........34

Figure S41. Measured data of $P_{4,4,4,4}$SPA + C_{70} (**1+C_{70}**) sensor response to synthetic air (green), ethanol (orange) and after exposure (blue)........34

Table S13. Parameters evaluated using CPE model for ethanol responses of sensors based on $P_{4,4,4,8}$SPA (**2**) and $P_{4,4,4,8}$SPA C_{60}/C_{70} (**2+C_{60}, 2+C_{70}**) nanocomposites.........34

Figure S42. Measured data of $P_{4,4,4,8}$SPA (**2**) sensor response to synthetic air (green), ethanol (orange) and after exposure (blue). 35

Figure S43. Measured data of $P_{4,4,4,8}$SPA + C_{60} (**2+C_{60}**) sensor response to synthetic air (green), ethanol (orange) and after exposure (blue)......36

Figure S44. Measured data of $P_{4,4,4,8}$SPA + C_{70} (**2+C_{70}**) sensor response to synthetic air (green), ethanol (orange) and after exposure (blue)......36

Table S14. Physical properties of the used analytes (dipole moment μ, relative polarity ETN, permittivity εr)......36

Figure S45. Linear dependence of the Y_0 value change on the relative permittivity of the analyte (acetic acid data excluded from linear regression)......37

Figure S46. Linear dependence of the Y_0 value change on the dipole moment of the analyte (acetic acid data excluded from linear regression)......38

Figure S47. Linear dependence of the Y_0 value change on the relative polarity of the analyte (acetic acid data excluded from linear regression)......38

Figure S48. Dependence of the change in $P_{4,4,4,X}$SPA sensor resistance (ΔR) on the dipole moment (μ) of various gas analytes......39

Figure S49. Interaction of $P_{4,4,4,4}$SPA upon sequential addition of explicit ethanol solvent molecules (1, 2, 5, 10, 20)......40

References......40

S1 Synthesis of monomeric ionic liquids $P_{4,4,4,4}$SPA and $P_{4,4,4,8}$SPA

All chemicals and solvents if not otherwise mentioned were purchased from chemical companies and were reagent grade. They were used without further purification or drying. All reactions were carried out under ambient conditions.

(i) **$P_{4,4,4,4}$SPA** was prepared by dissolving 4.0299 g of tetra-n-butyl-phosphonium chloride (Cytec, puriss. P.p.) in 40 ml distilled water and mixed with 4.7650 g of 3-sulfopropyl acrylate potassium salt (Sigma-Aldrich, 98.0%).

(ii) **$P_{4,4,4,8}$SPA** was prepared analogically using 5.0319 g of tributyl-octyl-phosphonium chloride (Cytec, puriss. P.p.) in 60 ml of distilled water which is then mixed with 5.0228 g of 3-sulfopropyl potassium acrylate potassium salt.

Both solutions were stirred at 30°C for 18 hours, followed by the extraction of tetra-n-butyl-phosphonium 3-sulfopropyl acrylate ($P_{4,4,4,4}$SPA) and tributyl-octyl-phosphonium 3-sulfopropyl acrylate ($P_{4,4,4,8}$SPA), respectively, from the aqueous phase using dichloromethane, three times in total (50 ml, 40 ml, and 40 ml). Dichloromethane was evaporated using a vacuum pump at a pressure of 50 mbar. The pure products (Fig. 1) were stored in dark vials at 4°C to prevent spontaneous polymerization.

S2 Preparation of the solution of C_{60} and C_{70} fullerenes

2.522 mg C_{60} (Sigma-Aldrich, 99.9%) and 2.943 mg C_{70} (Sigma-Aldrich, 98.0%) fullerenes were each dissolved in methylcyclohexane (Merck KGaA, 99.0%) leading to concentrations of 35 µM.[1] The prepared fullerene solutions were left to stand in dark for one day at room temperature.

S3 Polymerization of the ILs monomers

The polymerization mixtures were prepared from the monomeric IL solutions, $P_{4,4,4,4}$SPA or $P_{4,4,4,8}$SPA, respectively, which were then diluted with distilled water and additional acetonitrile, including an initiator and a cross-linker (volume ratio of monomer:water:acetonitrile 1:0.5:0.5). The starting solution for the nanocomposites of PILs with fullerenes were prepared by mixing the monomeric ILs solution with the prepared solution of fullerene C_{60} and C_{70}, respectively, in methylcyclohexane (concentration 35 µM) in a 1:1 volume ratio (Table 1). The mixture was covered with aluminium foil (to avoid irradiation by light and hence polymerization) and stirred until it was fully homogenized. Afterwards, the methylcyclohexane was evaporated under vacuum (50 mbar).

Phenyl-bis(2,4,6-trimethylbenzoyl)phosphine oxide (2% molar equivalent, Sigma-Aldrich, 97.0%) in acetonitrile was used as an initiator for the polymerization together with trimethylol-propane ethoxylate triacrylate oxide (1% molar equivalent, Sigma-Aldrich, 99.9%) as cross-linker. All amounts are given in Table S1.

Table S1. Polymerization mixture compositions.

Mixture	Monomer [mg]	Fullerene solution [µl]	Initiator [mg]	Crosslinker [µl]	Water [µl]	Acetonitrile [µl]
$P_{4,4,4,4}$SPA (**1**)	100	–	1.85	1.39	50	50
$P_{4,4,4,8}$SPA (**2**)	100	–	1.65	1.23	50	50
(**1**)+C_{60}	100	100	1.85	1.39	50	50
(**1**)+C_{70}	100	100	1.85	1.39	50	50
(**2**)+C_{60}	100	100	1.65	1.23	50	50
(**2**)+C_{70}	100	100	1.65	1.23	50	50

The prepared mixture was applied in form of one drop (1.5 µl) to the area between the gold electrodes on the substrate using an automatic pipette. After application, the droplet was immediately polymerized by exposure to white light using a Leica LMI-6000 Fiber-Lite with irradiation of 200 kLux for 45 minutes.

S4 Preparation of sensor substrates by magnetron sputtering of gold electrodes

The sensor substrates, which were used for electro-impedance spectroscopy, consisted of optical glass B270 (10 × 10 × 1 mm^3 UQG Optics). These substrates were cleaned in a first step with isopropyl alcohol, dried with a compressed air and etched in argon plasma (process pressure of 40 Pa, etching time 2.5 minutes, etching electric current 4 mA). Then, one pair of gold electrodes (1 mm gap, using a phosphor bronze mask) were applied on the substrate surface using magnetron sputtering deposition. (Fig. S1). A Denton DESK V magnetron sputtering system was used with the following process conditions: argon with purity 5.0 as the working gas, process pressure of 0.73 Pa, sputtering time 7.42 minutes, sputtering electric current 38 mA, Quartz Crystal Microbalance (Inficon SQM-160) measured thickness of 200 nm.

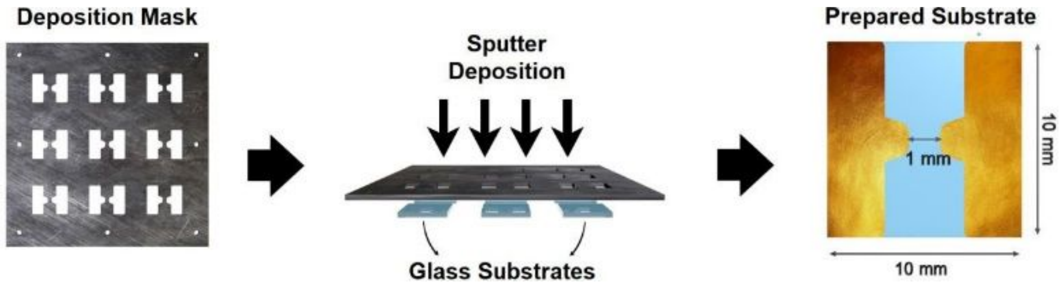

Figure S1. Preparation of gold electrodes on optical glass substrates via magnetron sputtering.

S5 Raman spectra of prepared PILs with/without C_{60} fullerene

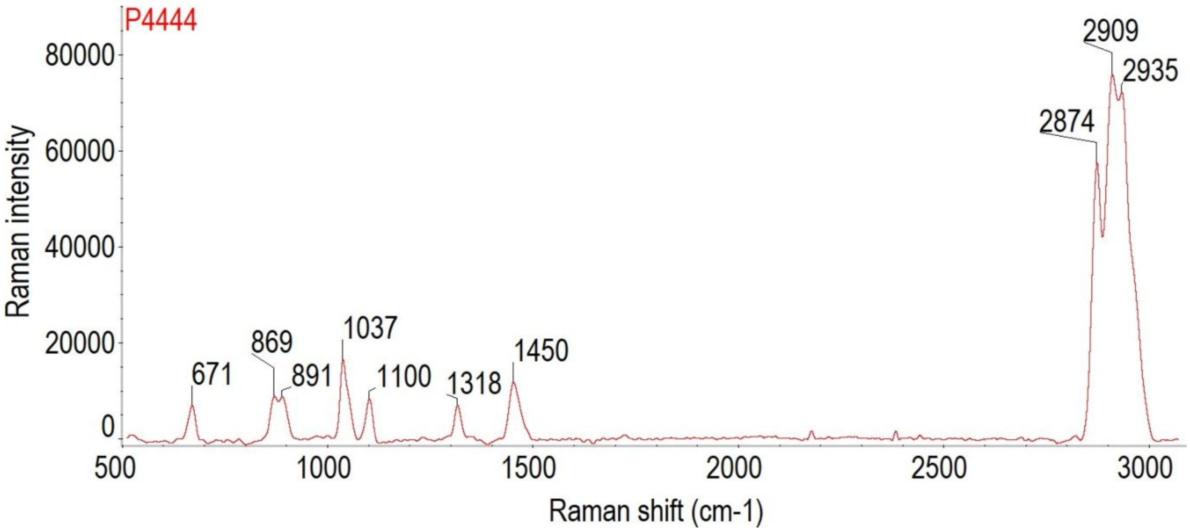

Figure S2. Raman spectrum of $P_{4,4,4,4}$SPA **(1)** in range 500 – 3000 cm^{-1}.

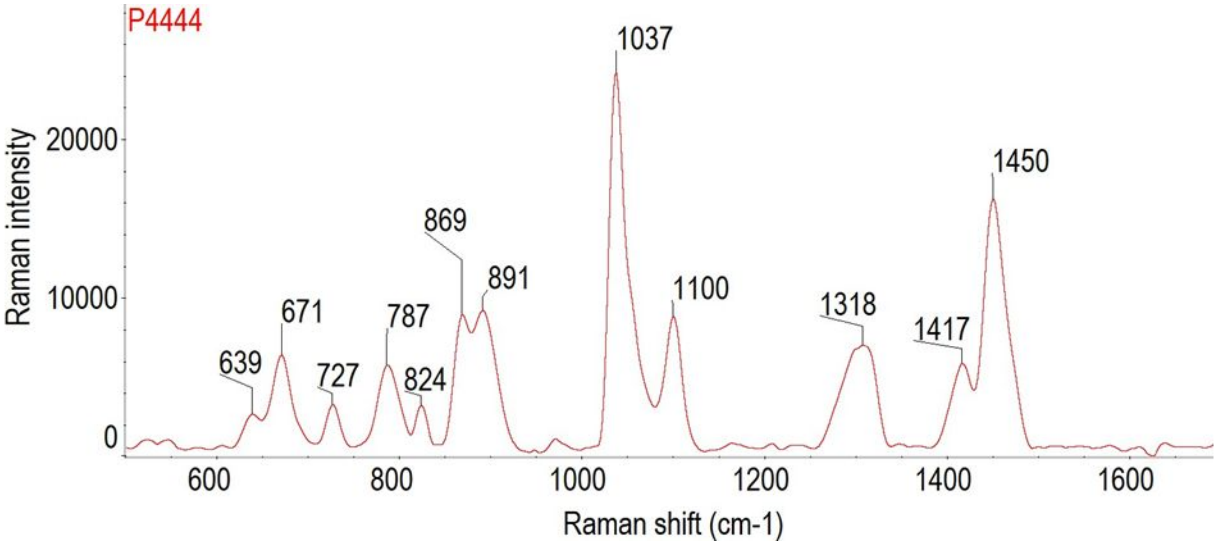

Figure S3. Raman spectrum of $P_{4,4,4,4}$SPA **(1)** in range 500 – 1700 cm^{-1}.

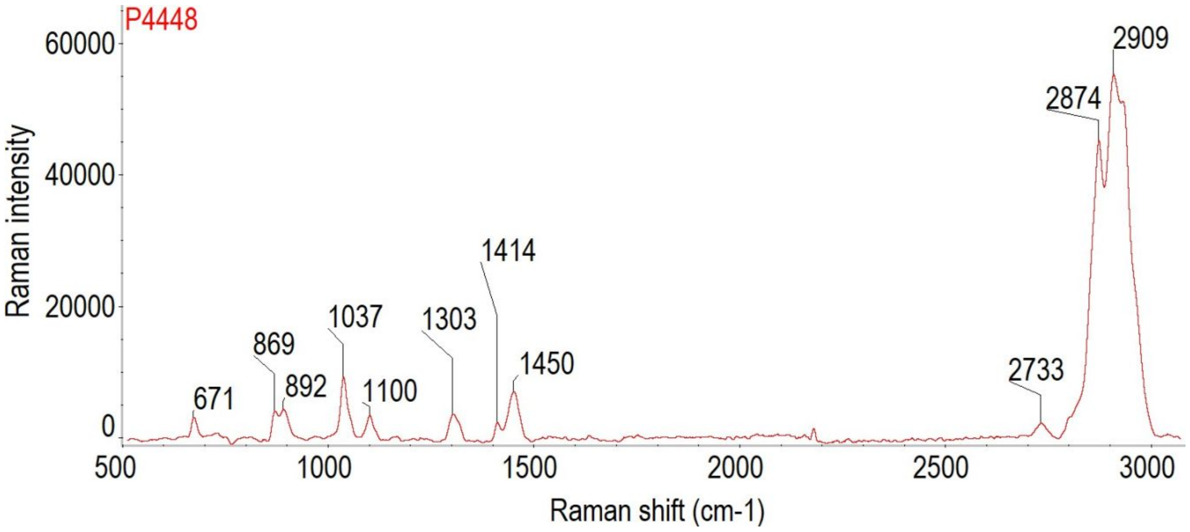

Figure S4. Raman spectrum of $P_{4,4,4,8}$SPA (**2**) in range 500 – 3000 cm^{-1}.

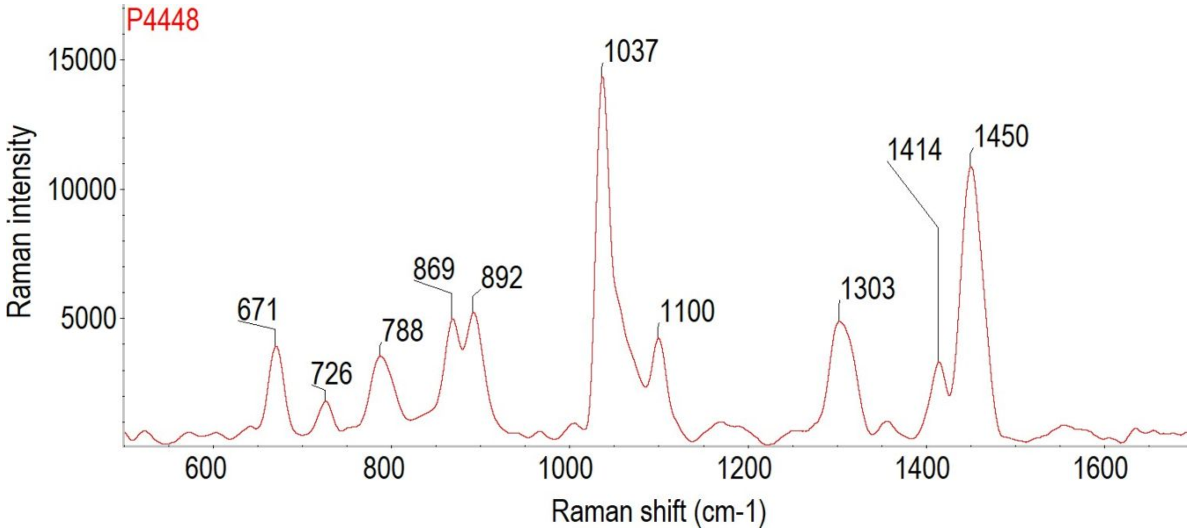

Figure S5. Raman spectrum of $P_{4,4,4,8}$SPA (**2**) in range 500 – 1700 cm^{-1}.

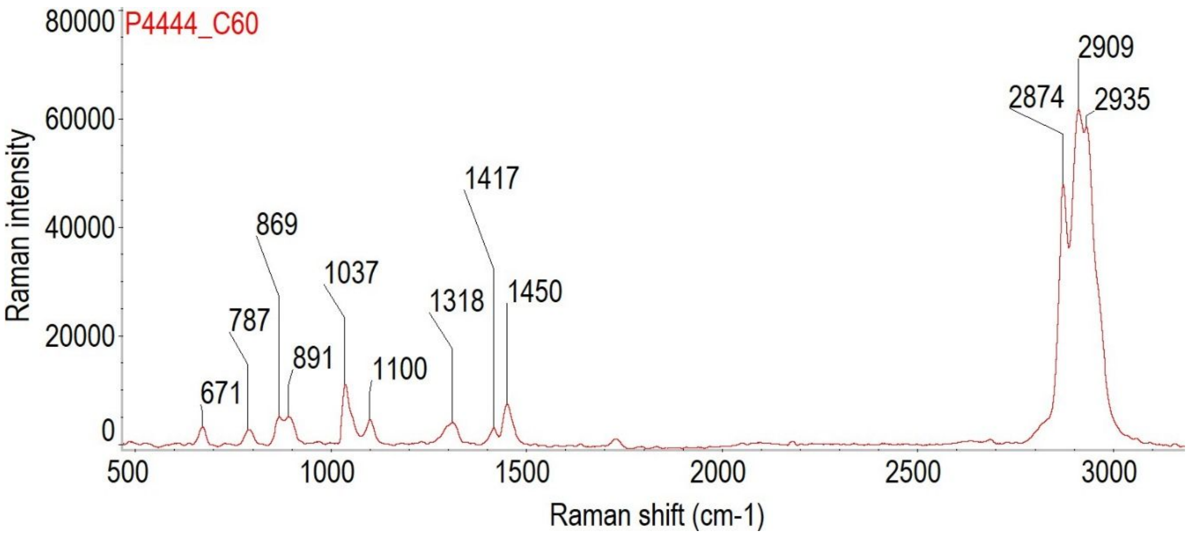

Figure S6. Raman spectrum of $P_{4,4,4,4}$SPA + C_{60} (**1+C_{60}**) in range 500 – 3000 cm^{-1}.

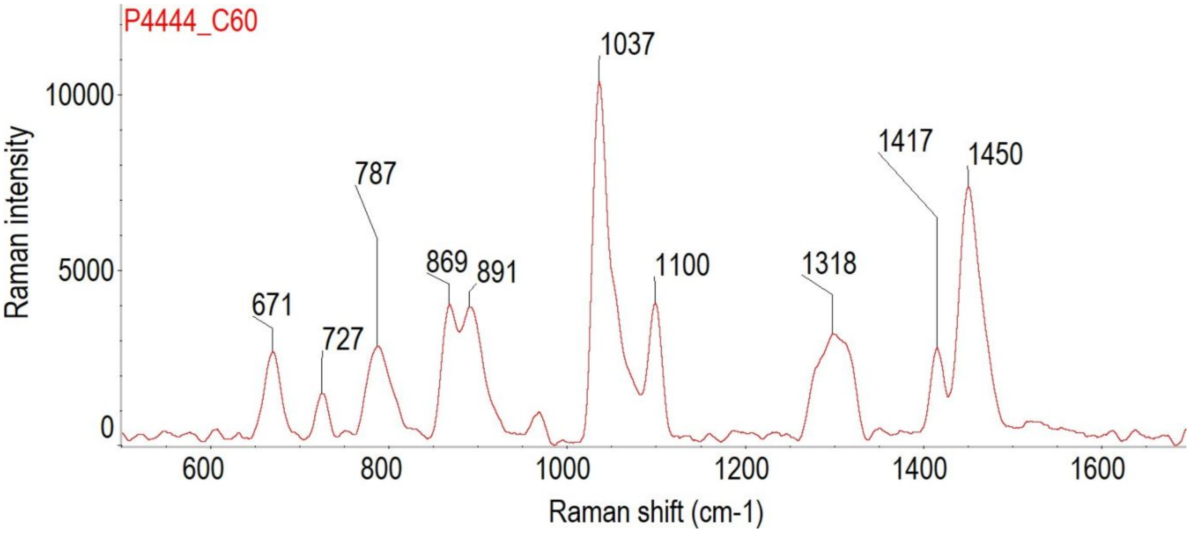

Figure S7. Raman spectrum of $P_{4,4,4,4}$SPA + C_{60} (**1+C_{60}**) in range 500 – 1700 cm^{-1}.

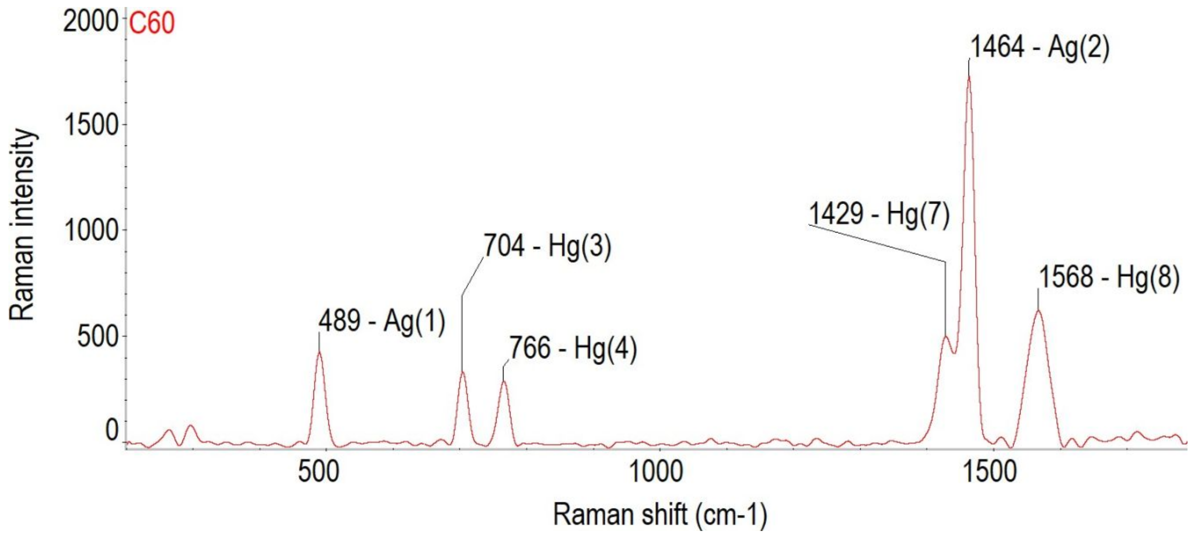

Figure S8. Raman spectrum of drop casted C_{60} dissolved in methylcyclohexane in the range between 200 – 1700 cm^{-1}.

Table S2. Representative Raman bands for $P_{4,4,4,4}$SPA (**1**) and $P_{4,4,4,8}$SPA (**2**) in the range between 3100 – 500 cm^{-1}.

**Assignment**	**Intensity**	**Functional group**	**$P_{4,4,4,4}$SPA (1)**	**$P_{4,4,4,8}$SPA (2)**	**$P_{4,4,4,4}$SPA + C_{60} (1+C_{60})**
ν_{as} (CH_3)	m	-CH_3	2935	2935	2935
ν_{as} (CH_2)	m	-CH_2-	2909	2909	2909
ν_s(CH_2)	m-s	-CH_2-	2874	2874	2874
δ_s(CH_3)	m-s	-CH_3	-	2733	-
δ(CH2)	w-m	-$(CH_2)_n$- n>2	1450, 1417	1450, 1414	1450, 1417
δ(CH2)	m	-$(CH_2)_n$-	1318	1303	1318
ν(CCC)	m-s	-$(CH_2)_n$-	1100, 1037	1100, 1037	1100, 1037
skeletal modes	m-s	-$(CH_2)_n$-	891, 869	892, 869	891, 869
ρ(CH2)	vw	-CH_2-CH_x, x≠2	787	788	787
ρ(CH2)	vw	-$(CH_2)_n$-(C)-, n>3	727	726	727
ν(P-C)	m	-(CH_2)-(P)-	671	671	671

S6 UV-Vis spectra of prepared PILs with/without C_{60} fullerene

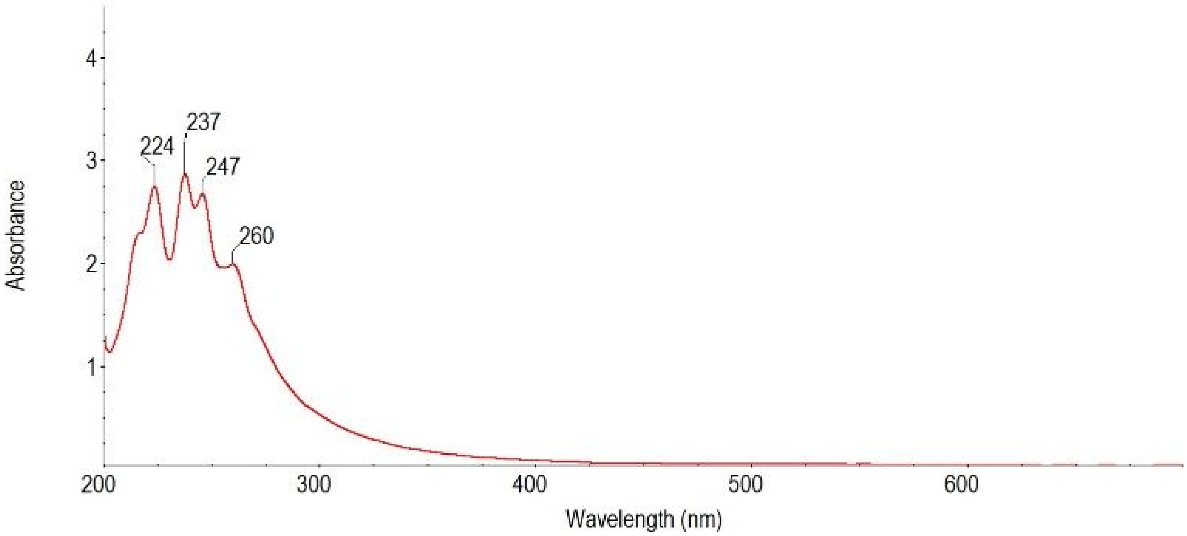

Figure S9. UV-vis absorbance spectrum of pure $P_{4,4,4,4}$SPA (**1**) in the range between 200 - 700 nm.

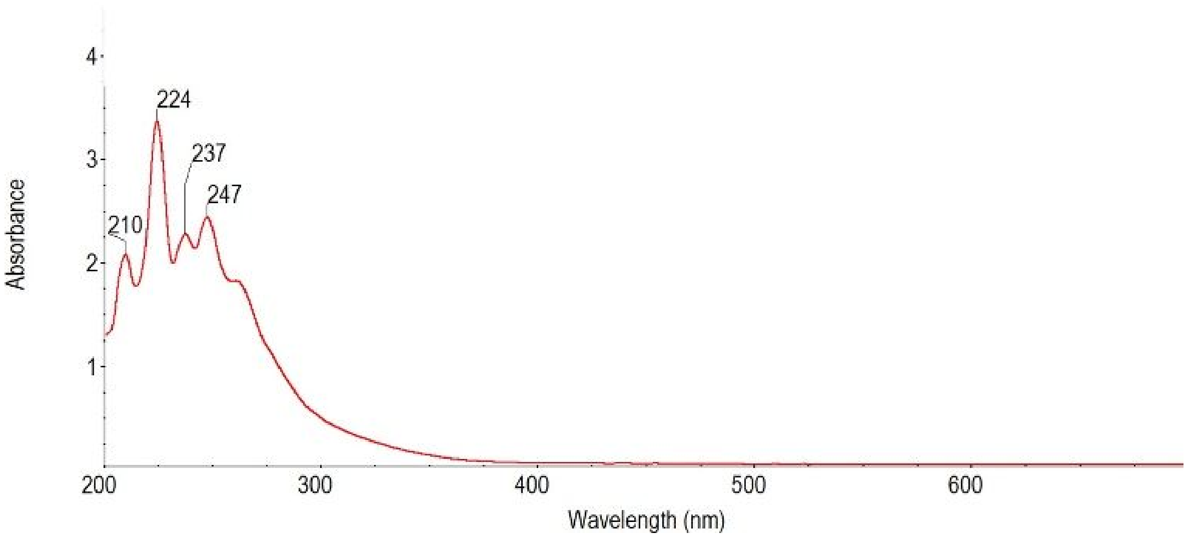

Figure S10. UV-Vis spectrum of pure $P_{4,4,4,8}$SPA (**2**) in the range between 200 - 700 nm.

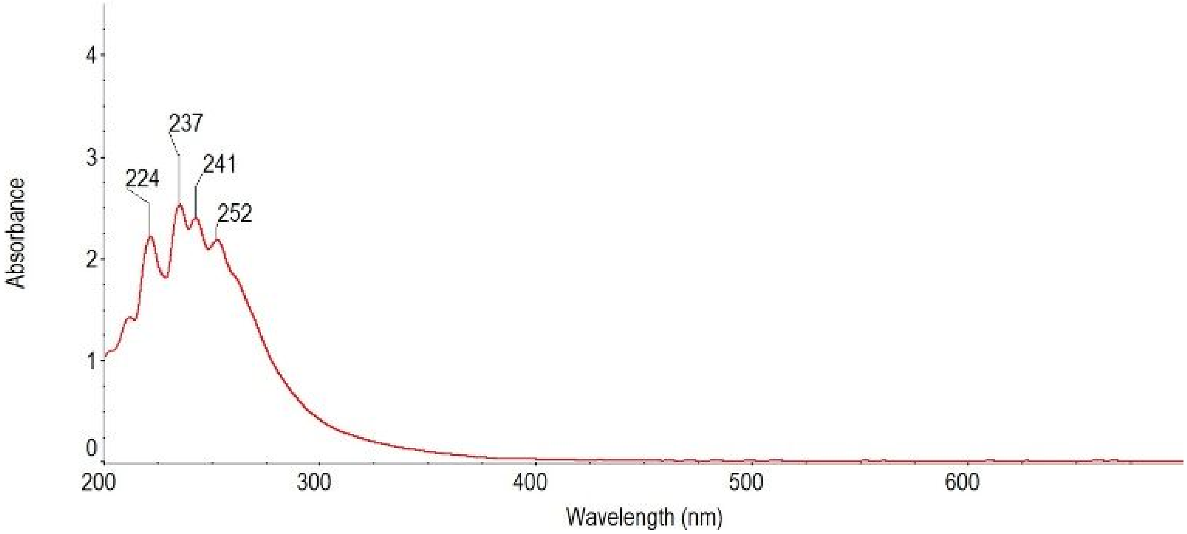

Figure S11. UV-VIS spectrum of nanocomposite $P_{4,4,4,4}$SPA + C_{60} (**1+C_{60}**) in the range between 200 - 700 nm.

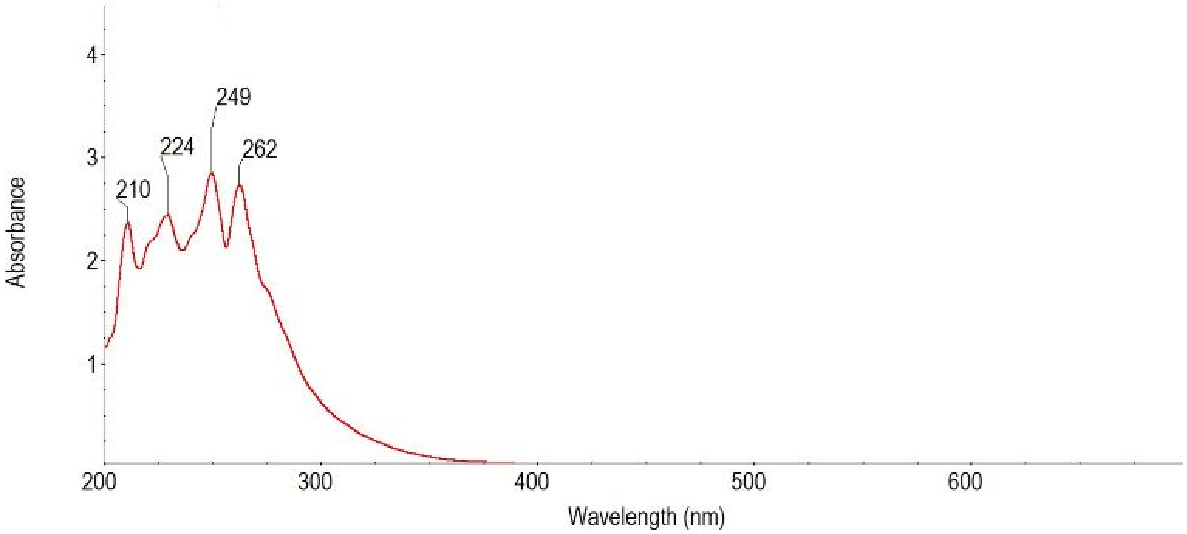

Figure S12. UV-Vis spectrum of nanocomposite $P_{4,4,4,8}$SPA + C_{60} (**2+C_{60}**) in the range between 200 - 700 nm.

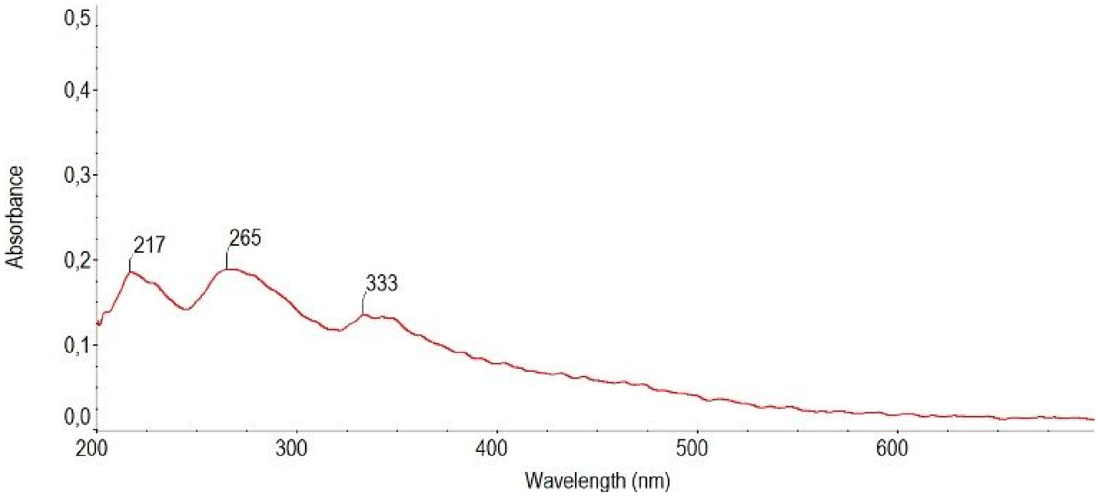

Figure S13. UV-Vis spectrum of drop casted C_{60} dissolved in methylcyclohexane in the range between 200 - 700 nm.

S7 EIS measurement and CPE model

The Randles circuit with Constant Phase Element (CPE) was used as a model for fitting of measured EIS data. The CPE matches different elements depending on the exponent $\boldsymbol{\alpha}$: at $\boldsymbol{\alpha}$ = 1, it acts as a capacitor ($\boldsymbol{Y_0}$ equals capacitance); at $\boldsymbol{\alpha}$ = 0, as a resistor ($\boldsymbol{Y_0}$ is the inverse of resistance); at $\boldsymbol{\alpha}$ = −1, as an inductor ($\boldsymbol{Y_0}$ is the inverse of inductance); and at $\boldsymbol{\alpha}$ = 0.5, it matches the Warburg impedance. It is possible to compare the diffusion coefficients of mobile ions in individual measurements by evaluating the value $\boldsymbol{Y_0}$, which is exactly inversely proportional to the Warburg coefficient when $\boldsymbol{\alpha}$ = 0.5. Therefore, $\boldsymbol{Y_0}$ is proportional to the diffusion coefficient ($\boldsymbol{Y_0} \sim \boldsymbol{D}_+$) and the exponent $\boldsymbol{\alpha}$ is related to the deviation from the Warburg impedance.

a)

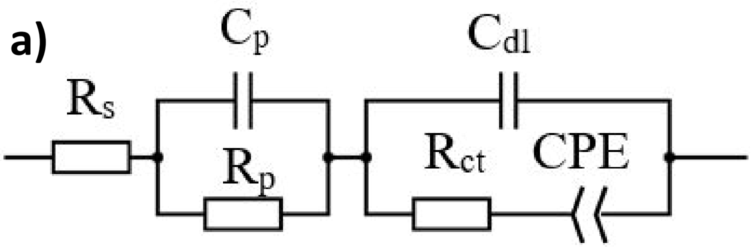

b)

$$\boldsymbol{Z_{CPE}} = \frac{1}{\boldsymbol{Y}_0(\boldsymbol{j}\omega)^{\boldsymbol{\alpha}}}$$

Figure S14. **a)** Equivalent circuit model used for the analysis of the poly(ionic liquid) sensor response. **b)** The equation represents the impedance of the CPE, where Y_0 is the admittance, α is the phase angle exponent.

The circuit consists of a solution resistance (R_s), a parallel combination of a polarization capacitance (C_p) and polarization resistance (R_p), and a parallel combination of a double-layer capacitance (C_{dl}) and a charge transfer resistance (R_{ct}), with a constant phase element (CPE) to account for non-ideal capacitive behavior.

Table S3. Top: Absolute values of Y_0 before and after analyte exposure, together with their difference (*with* $Y_0 \cdot 10^{-9}$ in S s$^{\alpha}$); Bottom: Relative changes, ΔY_0, of all PIL materials when exposed to the analyte (with ΔY_0 (%) = [(Y_0 (synthetic air) – Y_0 (analyte)) • 100] / Y_0 (synthetic air); the ΔY_0 values are given in relation to the reference Y_0 value in synthetic air), including the percentual change upon addition of fullerenes.

Y_0 (synthetic air), Y_0 (analyte) and the difference [Y_0 (synthetic air) – Y_0 (analyte)] *with* $Y_0 \cdot 10^{-9}$ (S s$^{\alpha}$)						
	**1**	**1** + C_{60}	**1** +C_{70}	**2**	**2** +C_{60}	**2** + C_{70}
	Ref, Gas, (Diff)	Ref, Gas, (Diff)	Ref, Gas, (Diff)	Ref, Gas, (Diff)	Ref, Gas, (Diff)	Ref, Gas, (Diff)
Acet-aldehyde	278, 248 (-30)	293, 264 (-29)	280, 236 (-44)	518, 486 (-32)	304, 273 (-31)	416, 377 (-39)
Acetic acid	184, 240 (+56)	306, 402 (+96)	254, 358 (+104)	396, 552 (+156)	277, 383 (+106)	238, 363 (+125)
Aceto-nitrile	278, 282 (+4)	211, 220 (+9)	208, 212 (+4)	518, 564 (+46)	251, 244 (-7)	256, 255 (-1)
Bromo-ethane	319, 238 (-81)	345, 252 (-93)	276, 195 (-81)	565, 391 (-174)	414, 323 (-91)	341, 236 (-105)
Ethanol	224, 206 (-18)	383, 346 (-37)	388, 346 (-42)	520, 472 (-48)	332, 292 (-40)	427, 360 (-67)

	ΔY_0 (%) = [(Y_0 (synthetic air) – Y_0 (analyte)) · 100] / Y_0 (synthetic air)					
	**1**	1 + C_{60}	1 +C_{70}	**2**	2 +C_{60}	2 + C_{70}
Acetaldehyde	**-10.79**	-9.90 (+8.2%)	-15.71 (-45.6%)	**-6.18**	-10.20 (-65.0%)	-9.38 (-51.8%)
Acetic acid	**30.43**	31.37 (+3.1%)	40.94 (+34.5%)	**39.39**	38.27 (-2.8%)	52.52 (+33.3%)
Acetonitrile	**1.44**	4.27 (+196.5%)	1.92 (+33.3%)	**8.88**	-2.79 (-131.4%)	-0.39 (-105.3%)
Bromoethane	**-25.39**	-26.96 (-6.2%)	-29.35 (-15.6%)	**-30.80**	-21.98 (+28.6%)	-30.79 (± 0%)
Ethanol	**-8.04**	-9.66 (-20.1%)	-10.82 (-34.6%)	**-9.23**	-12.05 (-30.6%)	-15.69 (-69.9%)

Table S4. Parameters evaluated using CPE model for acetaldehyde responses of sensors based on $P_{4,4,4,4}$SPA (**1**) and $P_{4,4,4,4}$SPA C_{60}/C_{70} (**1+C_{60}**, **1+C_{70}**) nanocomposites

**Sensor**	**[$P_{4,4,4,4}$][SPA] (1)**			**1 + C_{60}**			**1 + C_{70}**		
	**Ref.**	**AD***	**Ref.**	**Ref.**	**AD**	**Ref.**	**Ref.**	**AD**	**Ref.**
**$R_s \cdot 10^{-3}$ (Ω)**	< 1	26	326	229	< 1	449	6	26	18
**$R_p \cdot 10^4$ (Ω)**	102	107	102	39	49	39	64	87	68
**$R_{ct} \cdot 10^4$ (Ω)**	508	690	631	373	514	461	456	763	596
**$C_{dl} \cdot 10^{-14}$ (F)**	207	198	198	197	198	192	196	191	195
**$C_p \cdot 10^{-11}$ (F)**	483	629	571	490	385	608	488	407	534
**$Y_0 \cdot 10^{-9}$ (S s^{α})**	**278**	**248**	**251**	**293**	**264**	**273**	**280**	**236**	**256**
**α**	**0.65**	**0.63**	**0.63**	**0.70**	**0.67**	**0.69**	**0.65**	**0.62**	**0.64**

*AD = acetaldehyde

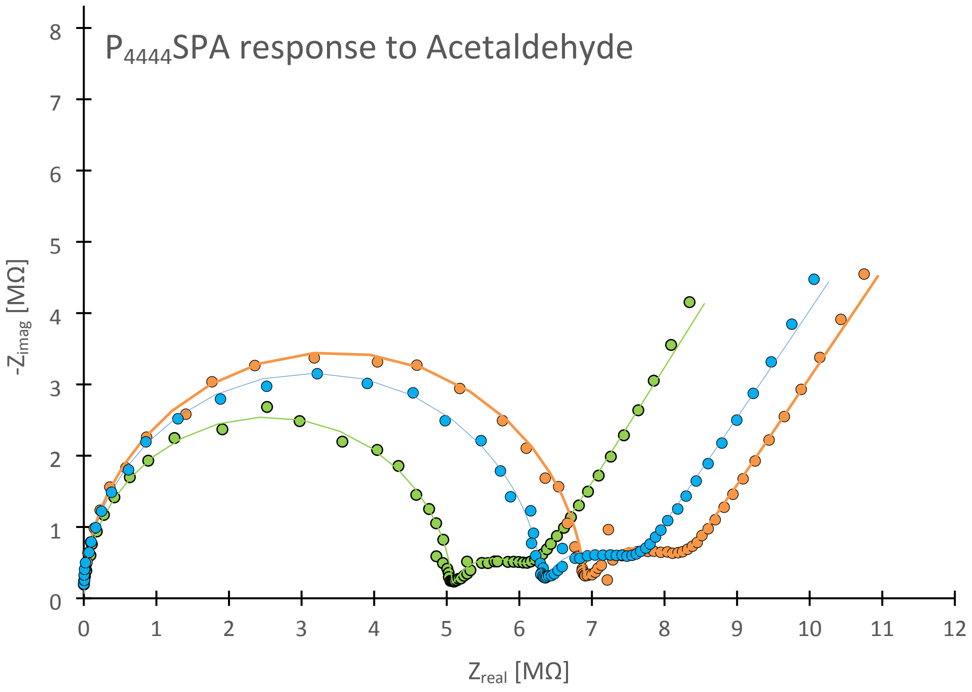

Figure S15. Measured data of $P_{4,4,4,4}$SPA (**1**) sensor response to synthetic air (green), acetaldehyde (orange) and after exposure (blue).

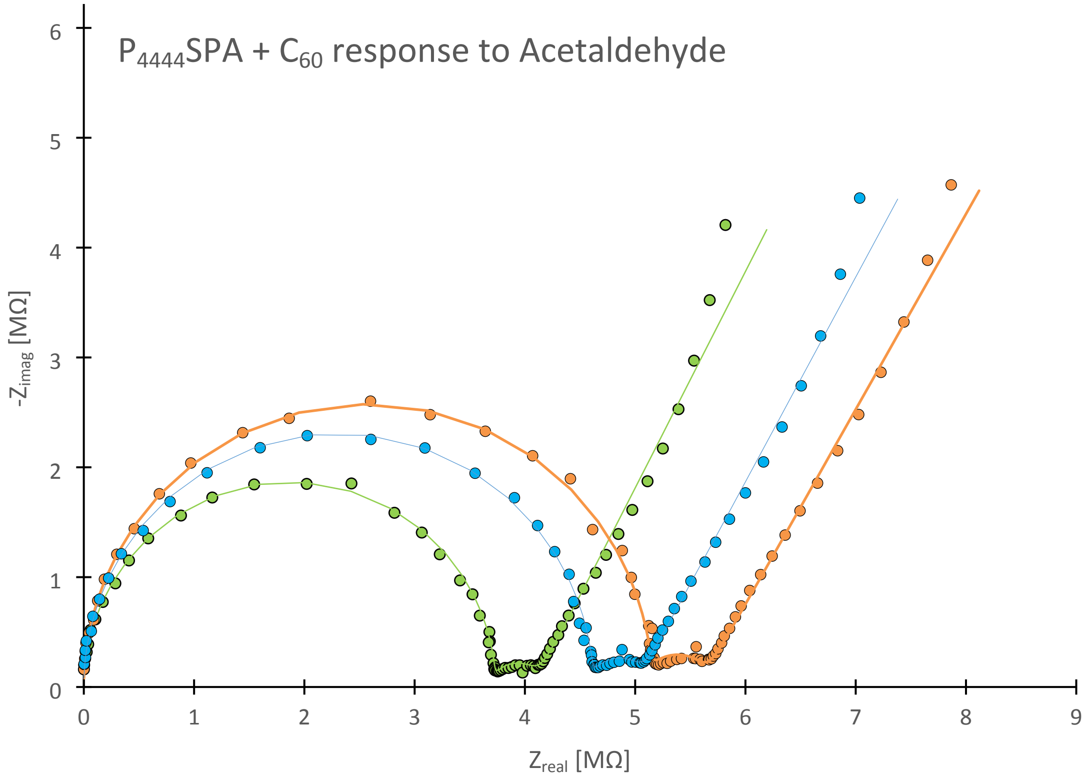

Figure S16. Measured data of $P_{4,4,4,4}$SPA + C_{60} (**1+C_{60}**) sensor response to synthetic air (green), acetaldehyde (orange) and after exposure (blue).

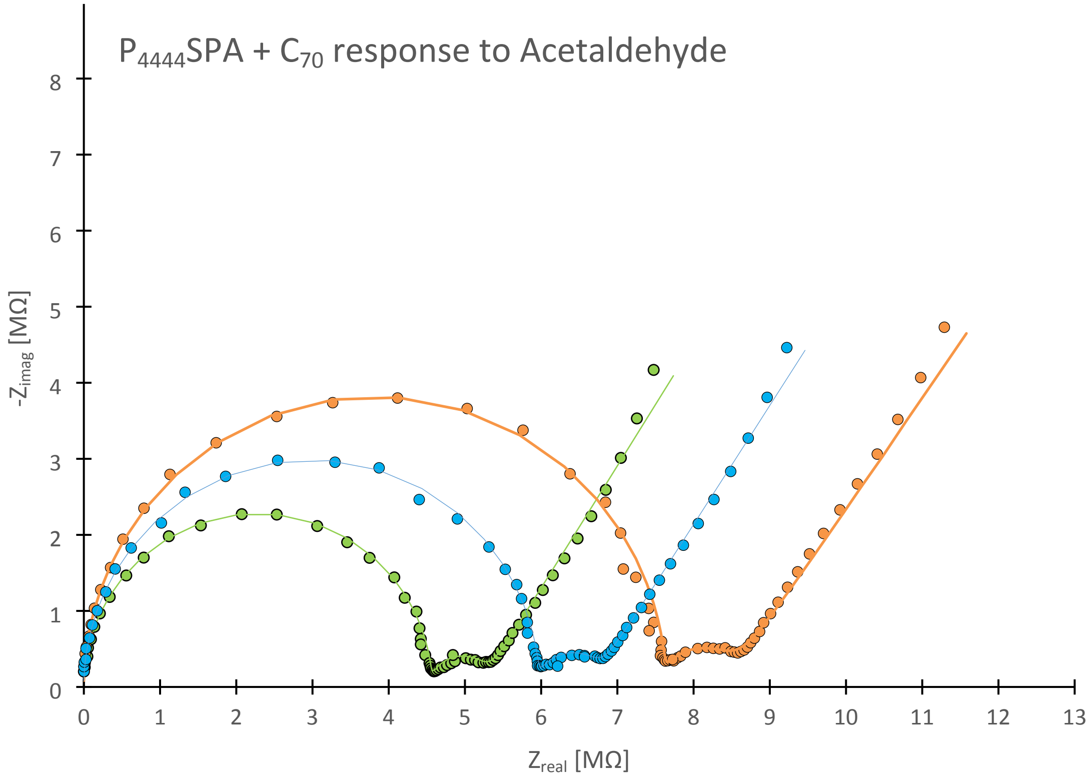

Figure S17. Measured data of $P_{4,4,4,4}$SPA + C_{70} (**1+C_{70}**) sensor response to synthetic air (green), acetaldehyde (orange) and after exposure (blue).

Table S5. Parameters evaluated using CPE model for acetaldehyde responses of sensors based on $P_{4,4,4,8}$SPA (**2**) and $P_{4,4,4,8}$SPA C_{60}/C_{70} (**2+C_{60}**, **2+C_{70}**) nanocomposites

Sensor	[$P_{4,4,4,8}$][SPA] (2)			2 + C_{60}			2 + C_{70}		
	Ref.	AD*	Ref.	Ref.	AD	Ref.	Ref.	AD	Ref.
$R_s \cdot 10^{-3}$ (Ω)	786	88	< 1	324	1	81	14	135	31
$R_p \cdot 10^4$ (Ω)	79	88	75	108	172	151	82	103	84
$R_{ct} \cdot 10^4$ (Ω)	386	466	437	651	1009	839	649	891	773
$C_{dl} \cdot 10^{-14}$ (F)	190	190	190	190	193	196	228	232	236
$C_p \cdot 10^{-11}$ (F)	784	739	878	660	482	449	481	388	475
$Y_0 \cdot 10^{-9}$ (S s^{α})	**518**	**486**	**478**	**304**	**273**	**286**	**416**	**377**	**376**
α	**0.63**	**0.62**	**0.60**	**0.52**	**0.57**	**0.56**	**0.55**	**0.57**	**0.54**

*AD = acetaldehyde

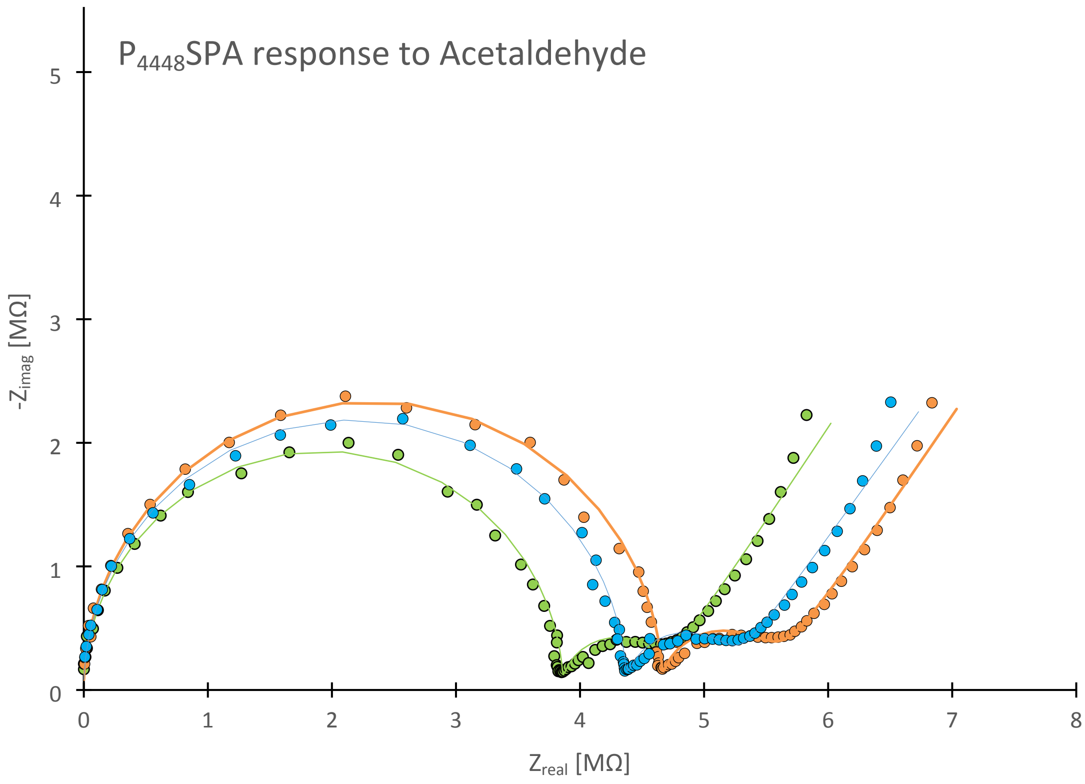

Figure S18. Measured data of $P_{4,4,4,8}SPA$ (**2**) sensor response to synthetic air (green), acetaldehyde (orange) and after exposure (blue).

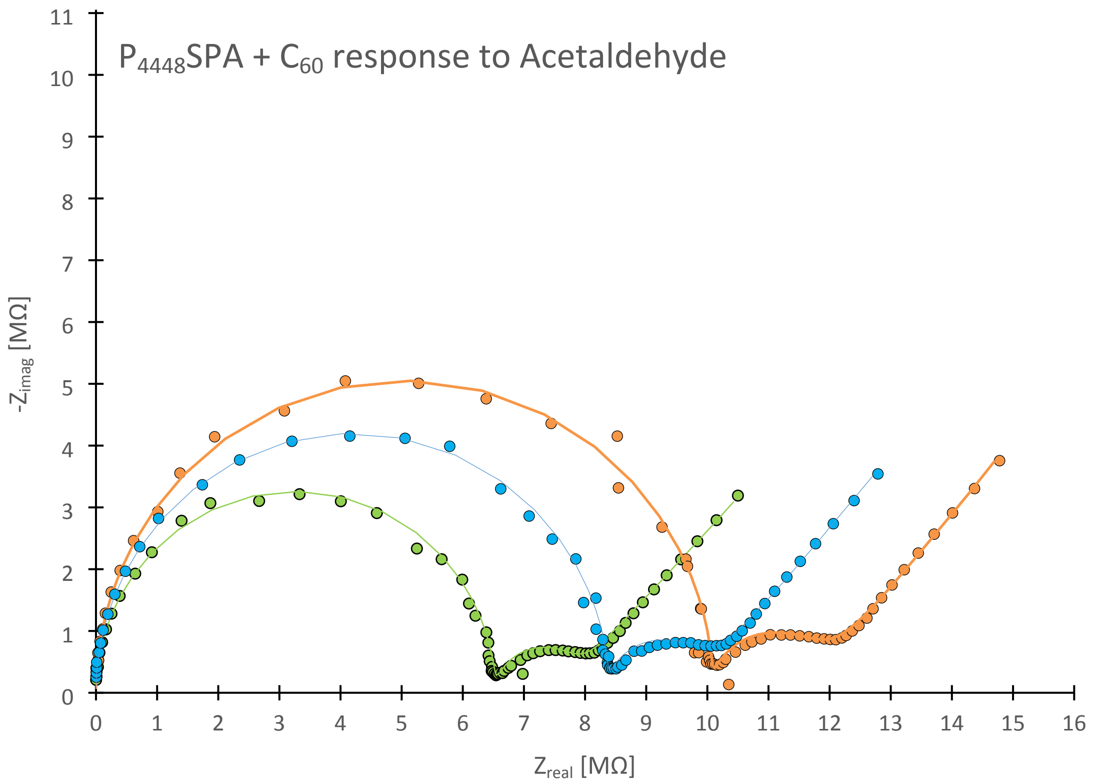

Figure S19. Measured data of $P_{4,4,4,8}$SPA + C_{60} (**2+C_{60}**) sensor response to synthetic air (green), acetaldehyde (orange) and after exposure (blue).

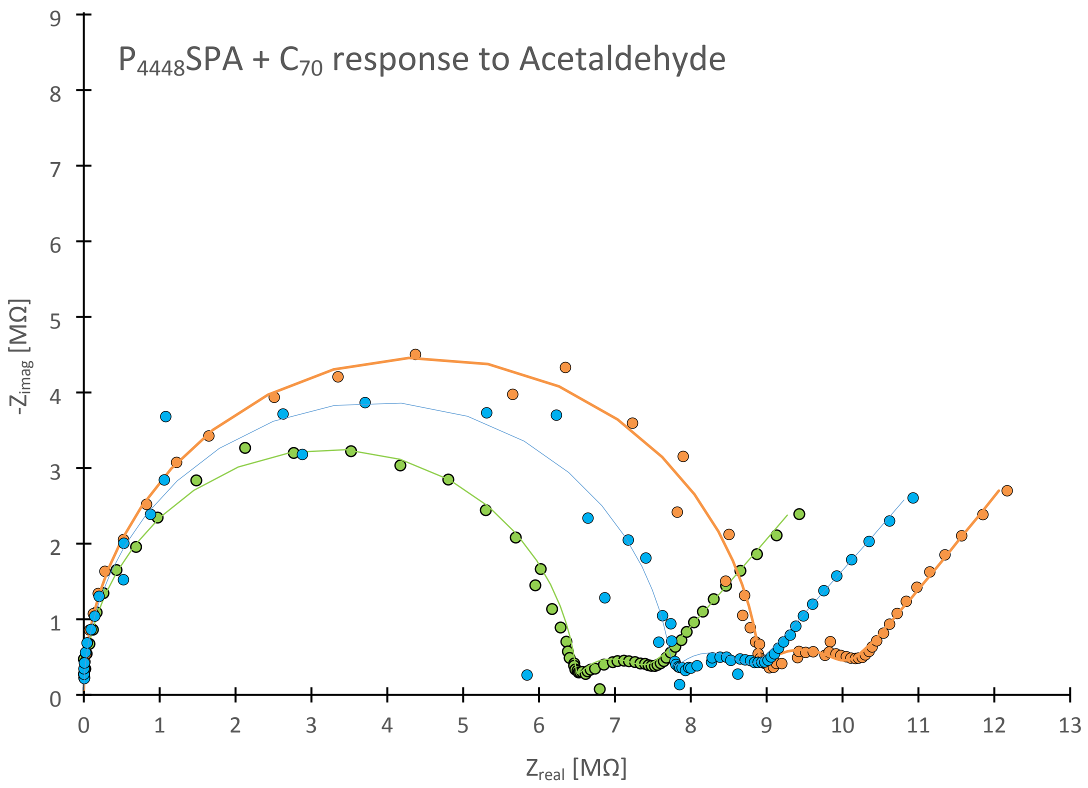

Figure S20. Measured data of $P_{4,4,4,8}$SPA + C_{70} (**2+C_{70}**) sensor response to synthetic air (green), acetaldehyde (orange) and after exposure (blue).

Table S6. Parameters evaluated using CPE model for acetic acid responses of sensors based on $P_{4,4,4,4}$SPA (**1**) and $P_{4,4,4,4}$SPA C_{60}/C_{70} (**1+C_{60}**, **1+C_{70}**) nanocomposites

Sensor	[$P_{4,4,4,4}$][SPA] (1)			1 + C_{60}			1 + C_{70}		
	Ref.	AA	Ref.	Ref.	AA	Ref.	Ref.	AA	Ref.
$R_s \cdot 10^{-3}$ (Ω)	< 1	282	693	62	282	219	3	10	< 1
$R_p \cdot 10^{4}$ (Ω)	121	94	90	54	53	54	94	78	69
$R_{ct} \cdot 10^{4}$ (Ω)	729	463	412	518	437	371	679	453	381
$C_{dl} \cdot 10^{-14}$ (F)	191	192	191	194	194	195	198	198	195
$C_p \cdot 10^{-11}$ (F)	549	688	665	601	758	574	624	947	910
$Y_0 \cdot 10^{-9}$ (S s^{α})	**184**	**240**	**242**	**306**	**402**	**416**	**254**	**358**	**360**
α	**0.68**	**0.79**	**0.78**	**0.57**	**0.62**	**0.62**	**0.61**	**0.72**	**0.71**

*AA = acetic acid

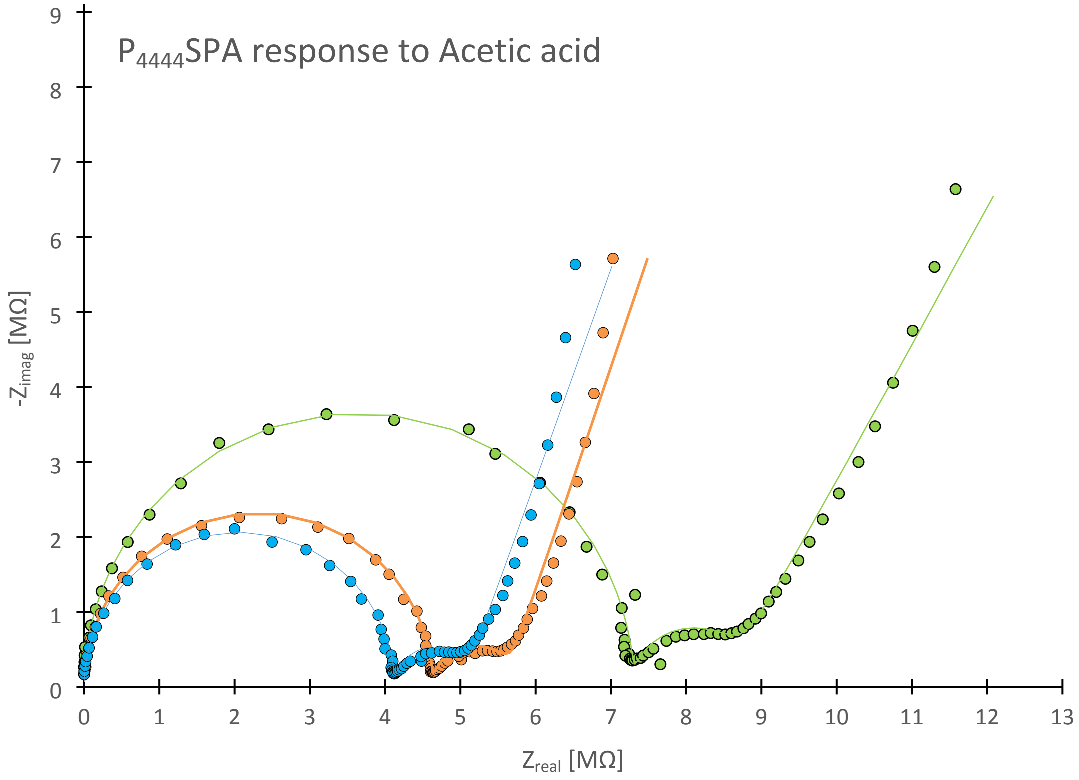

Figure S21. Measured data of $P_{4,4,4,4}$SPA (**1**) sensor response to synthetic air (green), acetic acid (orange) and after exposure (blue).

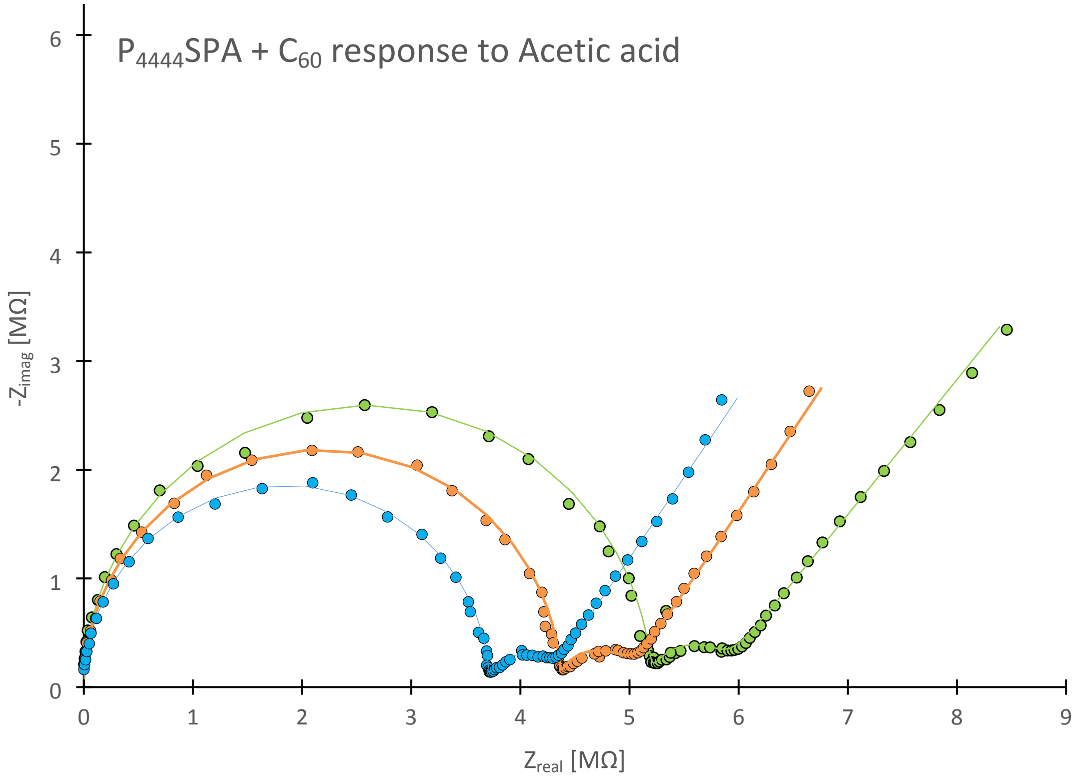

Figure S22. Measured data of $P_{4,4,4,4}$SPA + C_{60} (**1+C_{60}**) sensor response to synthetic air (green), acetic acid (orange) and after exposure (blue).

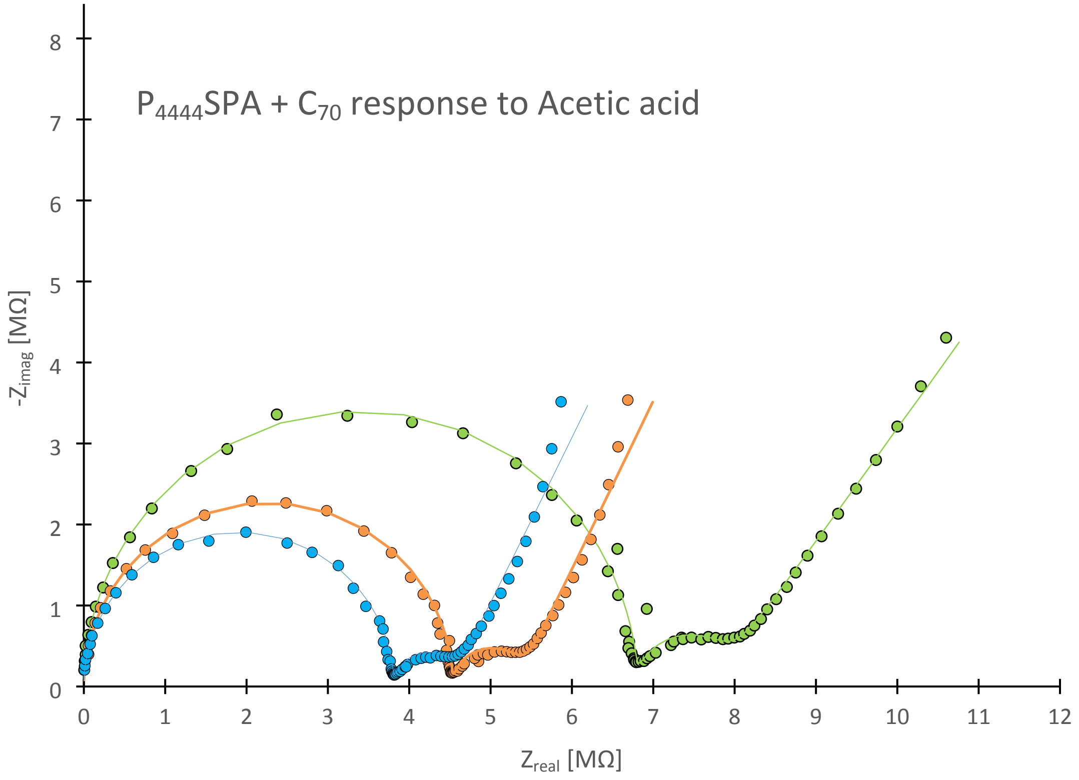

Figure S23. Measured data of $P_{4,4,4,4}$SPA + C_{70} (**1+C_{70}**) sensor response to synthetic air (green), acetic acid (orange) and after exposure (blue).

Table S7. Parameters evaluated using CPE model for acetic acid responses of sensors based on $P_{4,4,4,8}$SPA (**2**) and $P_{4,4,4,8}$SPA C_{60}/C_{70} (**2+C_{60}**, **2+C_{70}**) nanocomposites

Sensor	[$P_{4,4,4,8}$][SPA] (2)			2 + C_{60}			2 + C_{70}		
	Ref.	AA	Ref.	Ref.	AA	Ref.	Ref.	AA	Ref.
$R_s \cdot 10^{-3}$ (Ω)	534	16	74	9	3	202	1226	1437	150
$R_p \cdot 10^{4}$ (Ω)	108	87	82	72	60	54	125	89	78
$R_{ct} \cdot 10^{4}$ (Ω)	286	193	172	742	542	478	298	187	155
$C_{dl} \cdot 10^{-14}$ (F)	191	191	192	197	197	196	211	211	212
$C_p \cdot 10^{-11}$ (F)	636	927	913	451	928	926	538	1604	1597
$Y_0 \cdot 10^{-9}$ (S s^{α})	**396**	**552**	**555**	**277**	**383**	**387**	**238**	**363**	**370**
α	**0.51**	**0.59**	**0.58**	**0.60**	**0.68**	**0.68**	**0.42**	**0.51**	**0.50**

*AA = acetic acid

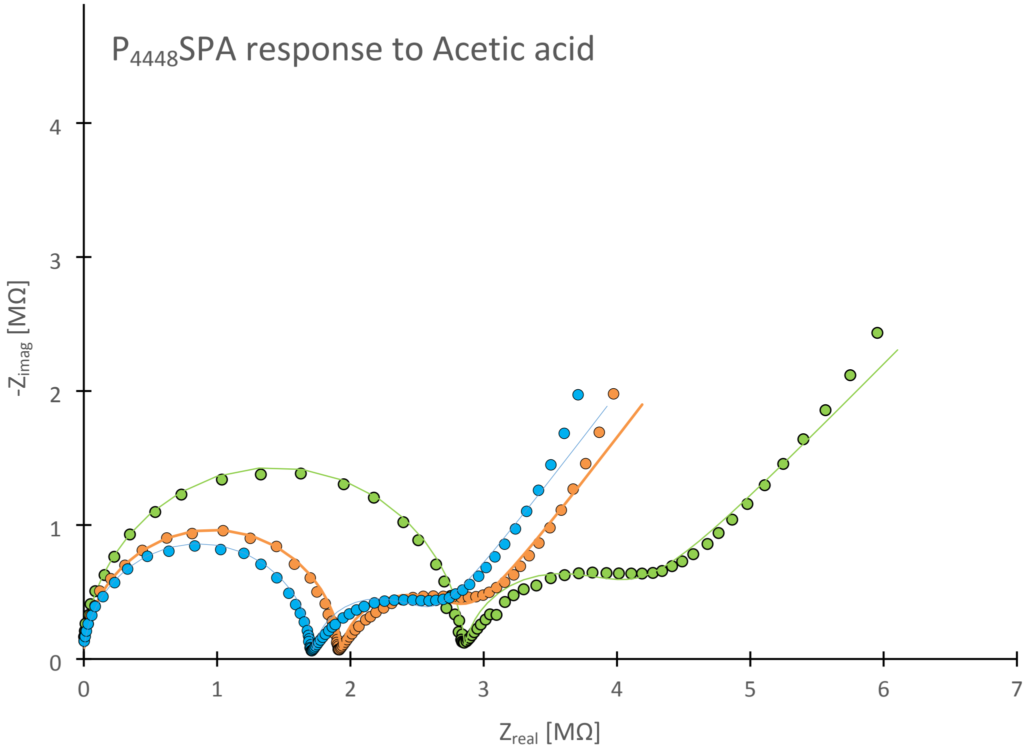

Figure S24. Measured data of $P_{4,4,4,8}$SPA (**2**) sensor response to synthetic air (green), acetic acid (orange) and after exposure (blue).

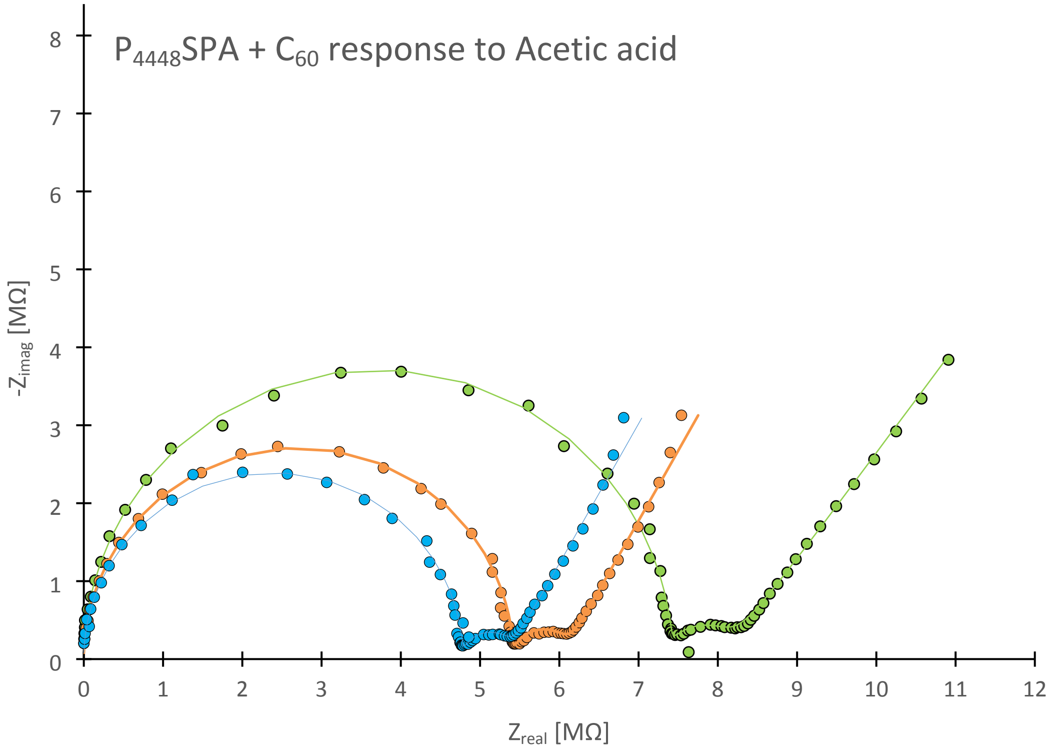

Figure S25. Measured data of $P_{4,4,4,8}$SPA + C_{60} (**2+C₆₀**) sensor response to synthetic air (green), acetic acid (orange) and after exposure (blue).

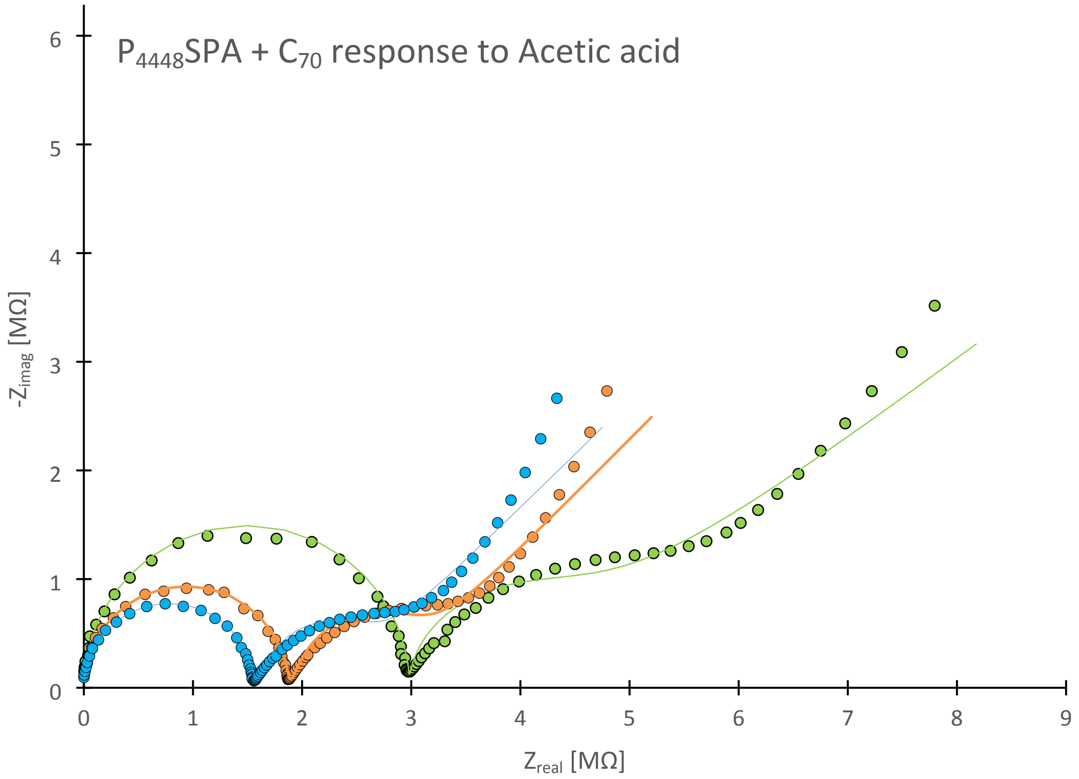

Figure S26. Measured data of $P_{4,4,4,8}$SPA + C_{70} (**2+C_{70}**) sensor response to synthetic air (green), acetic acid (orange) and after exposure (blue).

Table S8. Parameters evaluated using CPE model for acetonitrile responses of sensors based on $P_{4,4,4,4}$SPA (**1**) and $P_{4,4,4,4}$SPA C_{60}/C_{70} (**1+C_{60}**, **1+C_{70}**) nanocomposites

Sensor	$[P_{4,4,4,4}]$[SPA] (1)			1 + C_{60}			1 + C_{70}		
	Ref.	AN*	Ref.	Ref.	AN	Ref.	Ref.	AN	Ref.
$R_s \cdot 10^{-3}$ (Ω)	< 1	< 1	2	< 1	194	1	4	16	550
$R_p \cdot 10^4$ (Ω)	95	96	102	134	145	133	103	120	113
$R_{ct} \cdot 10^4$ (Ω)	696	676	687	1126	989	1017	1129	1129	974
$C_{dl} \cdot 10^{-14}$ (F)	174	172	172	178	177	176	188	186	180
$C_p \cdot 10^{-11}$ (F)	727	709	652	791	518	687	570	330	370
$Y_0 \cdot 10^{-9}$ (S s^{α})	**278**	**282**	**278**	**211**	**220**	**212**	**208**	**212**	**220**
α	**0.69**	**0.68**	**0.70**	**0.63**	**0.63**	**0.62**	**0.61**	**0.62**	**0.61**

*AN = acetonitrile

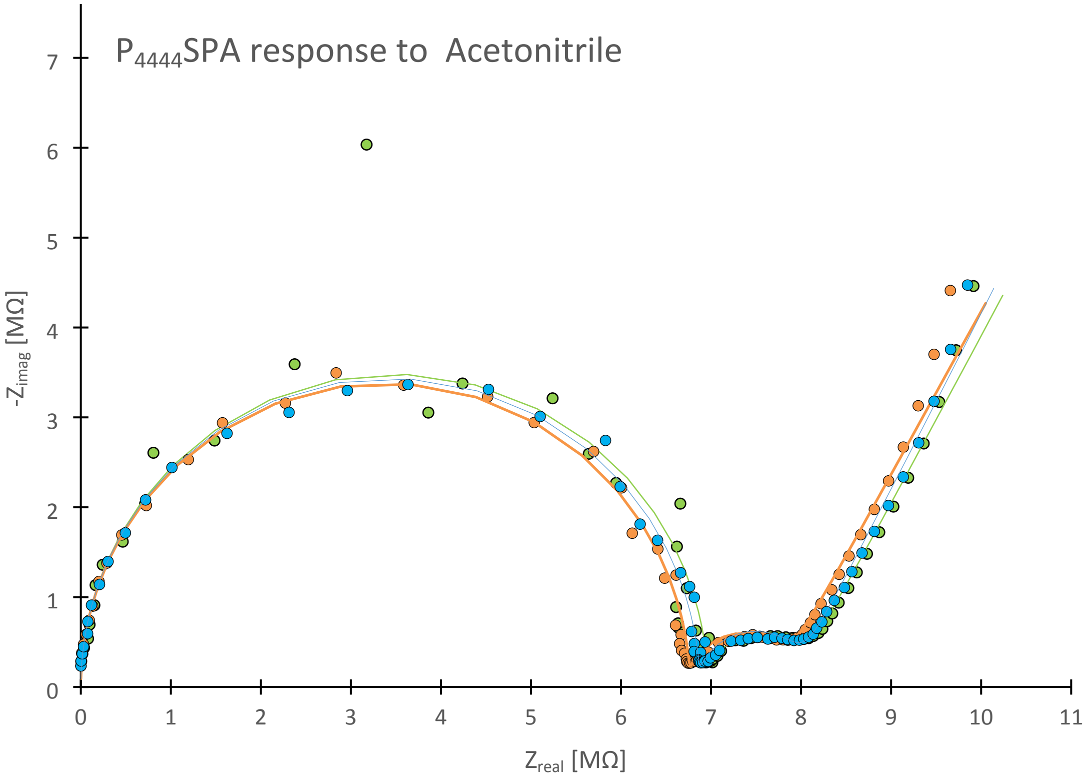

Figure S27. Measured data of $P_{4,4,4,4}$SPA (**1**) sensor response to synthetic air (green), acetonitrile (orange) and after exposure (blue).

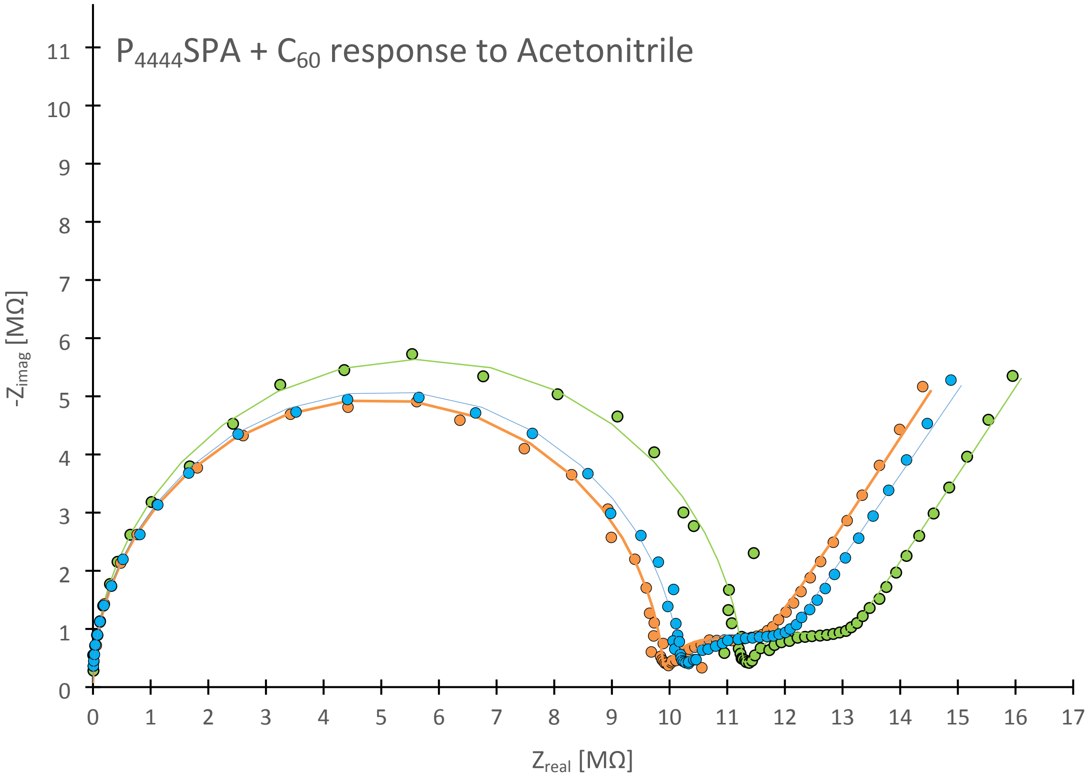

Figure S28. Measured data of $P_{4,4,4,4}$SPA + C_{60} (**1+C_{60}**) sensor response to synthetic air (green), acetonitrile (orange) and after exposure (blue).

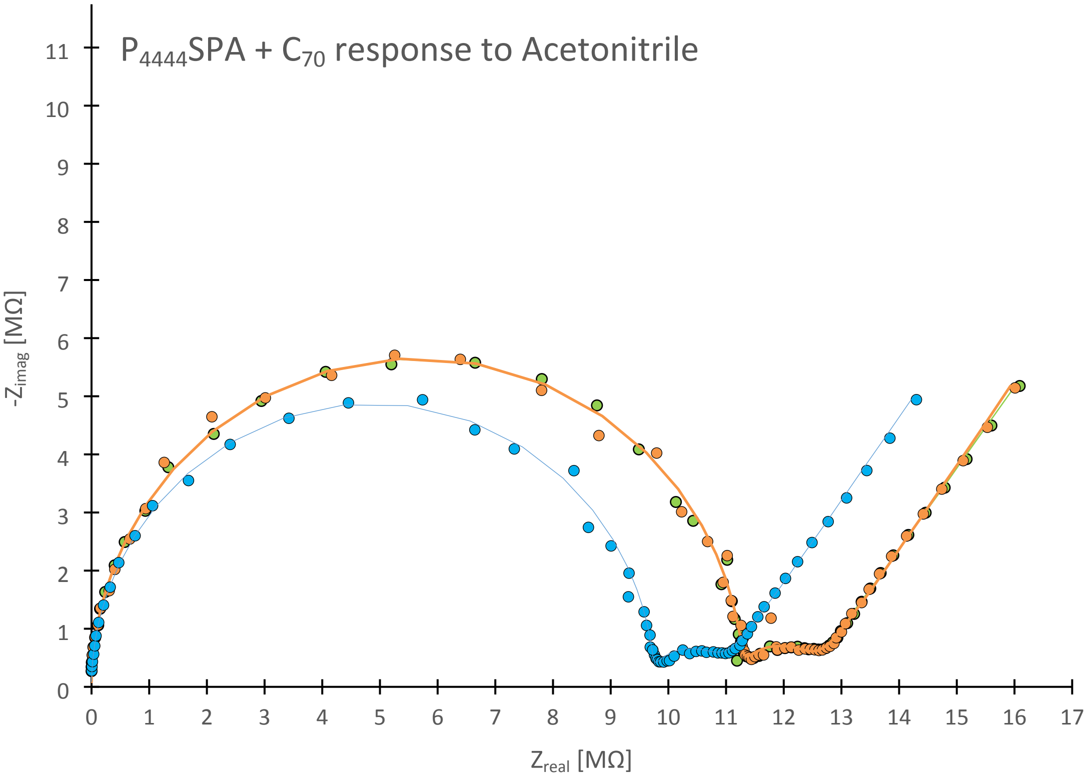

Figure S29. Measured data of $P_{4,4,4,4}$SPA + C_{70} (**1+C_{70}**) sensor response to synthetic air (green), acetonitrile (orange) and after exposure (blue).

Table S9. Parameters evaluated using CPE model for acetonitrile responses of sensors based on $P_{4,4,4,8}$SPA (**2**) and $P_{4,4,4,8}$SPA C_{60}/C_{70} (**2+C_{60}**, **2+C_{70}**) nanocomposites.

**Sensor**	**[$P_{4,4,4,8}$][SPA]**			**[$P_{4,4,4,8}$][SPA] + C_{60}**			**[$P_{4,4,4,8}$][SPA] + C_{70}**		
	**Ref.**	**AN***	**Ref.**	**Ref.**	**AN**	**Ref.**	**Ref.**	**AN**	**Ref.**
**$R_s \cdot 10^{-3}$ (Ω)**	< 1	166	65	1	240	< 1	323	7383	470
**$R_p \cdot 10^4$ (Ω)**	70	74	78	134	148	134	135	147	140
**$R_{ct} \cdot 10^4$ (Ω)**	462	401	428	456	496	522	996	1043	1033
**$C_{dl} \cdot 10^{-14}$ (F)**	177	177	176	183	187	184	182	182	182
**$C_p \cdot 10^{-11}$ (F)**	1114	660	681	557	550	740	400	472	437
**$Y_0 \cdot 10^{-9}$ (S $s^α$)**	**518**	**564**	**557**	**251**	**244**	**238**	**256**	**255**	**253**
**α**	**0.48**	**0.49**	**0.50**	**0.66**	**0.66**	**0.65**	**0.59**	**0.60**	**0.59**

*AN = acetonitrile

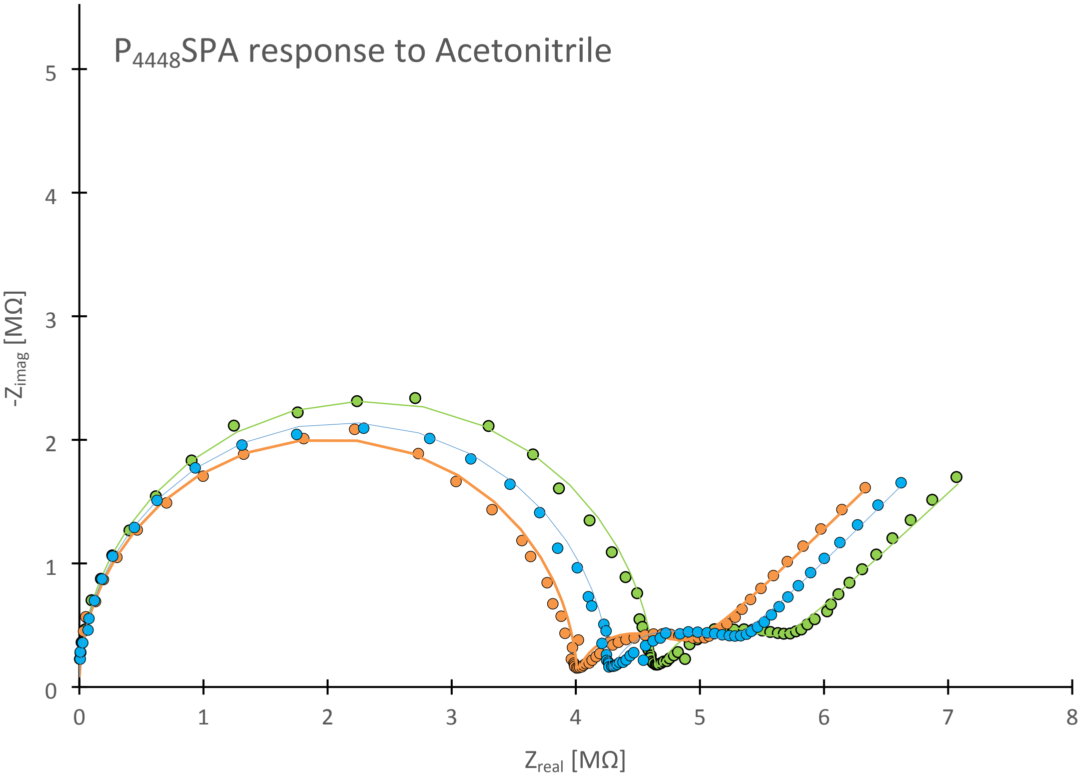

Figure S30. Measured data of $P_{4,4,4,8}$SPA (**2**) sensor response to synthetic air (green), acetonitrile (orange) and after exposure (blue).

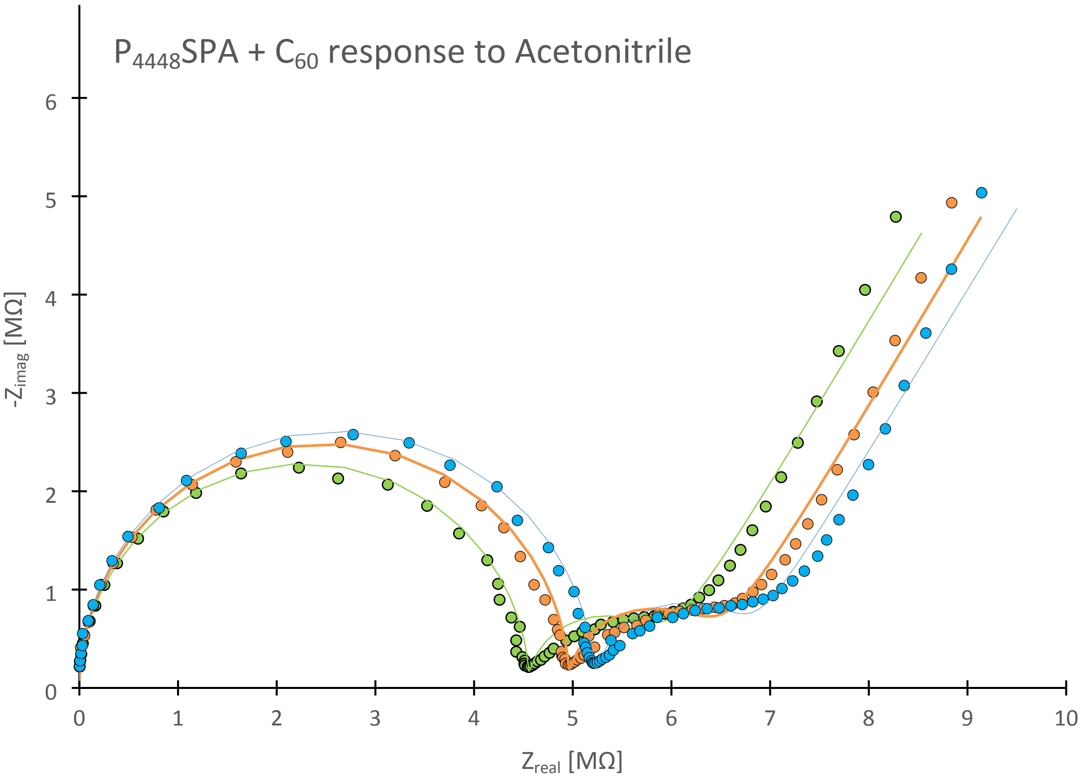

Figure S31. Measured data of $P_{4,4,4,8}$SPA + C_{60} (**2+C_{60}**) sensor response to synthetic air (green), acetonitrile (orange) and after exposure (blue).

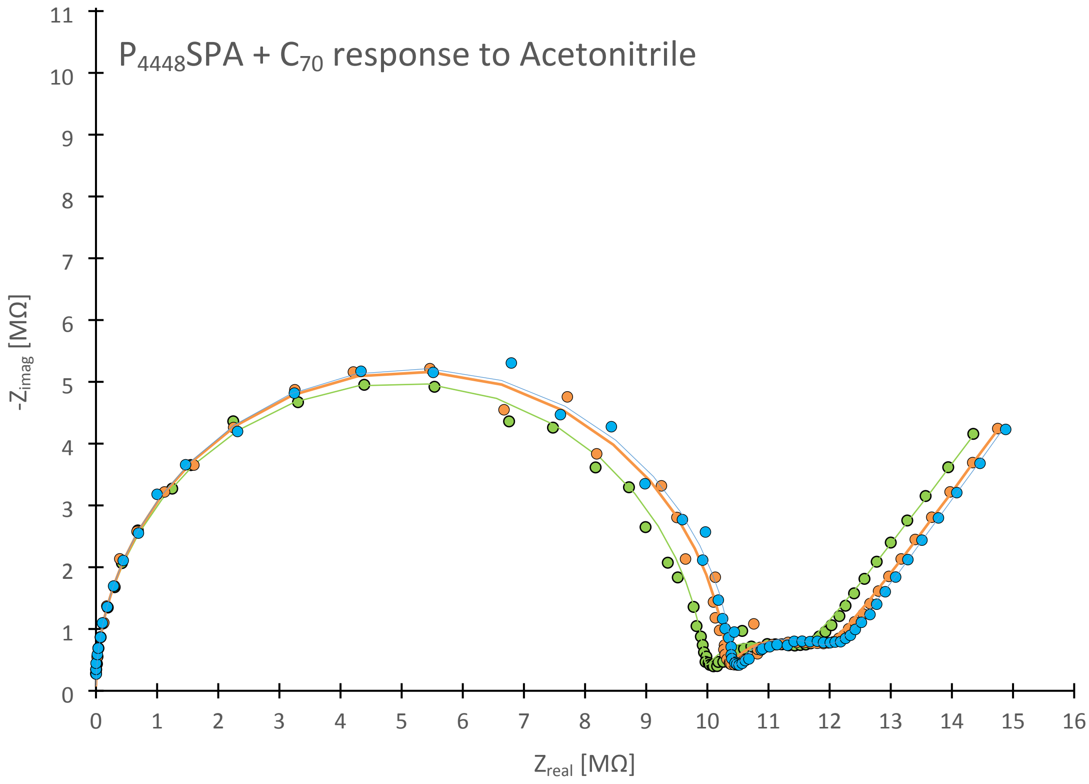

Figure S32. Measured data of $P_{4,4,4,8}$SPA + C_{70} (**2+C_{70}**) sensor response to synthetic air (green), acetonitrile (orange) and after exposure (blue).

Table S10. Parameters evaluated using CPE model for bromoethane responses of sensors based on $P_{4,4,4,4}$SPA (**1**) and $P_{4,4,4,4}$SPA C_{60}/C_{70} (**1+C_{60}**, **1+C_{70}**) nanocomposites.

Sensor	[$P_{4,4,4,4}$][SPA] (1)			1 + C_{60}			1 + C_{70}		
	**Ref.**	**BE***	**Ref.**	**Ref.**	**BE**	**Ref.**	**Ref.**	**BE**	**Ref.**
**$R_s \cdot 10^{-3}$ (Ω)**	268	< 1	8	271	< 1	3	1041	224	65
**$R_p \cdot 10^{4}$ (Ω)**	52	94	130	50	62	72	75	137	149
**$R_{ct} \cdot 10^{4}$ (Ω)**	522	685	602	232	324	282	439	625	526
**$C_{dl} \cdot 10^{-14}$ (F)**	159	167	154	184	181	185	222	198	194
**$C_p \cdot 10^{-11}$ (F)**	344	644	483	581	739	921	509	741	827
**$Y_0 \cdot 10^{-9}$ (S s$^{\alpha}$)**	**319**	**238**	**235**	**345**	**252**	**238**	**276**	**195**	**189**
**α**									

*BE = bromoethane

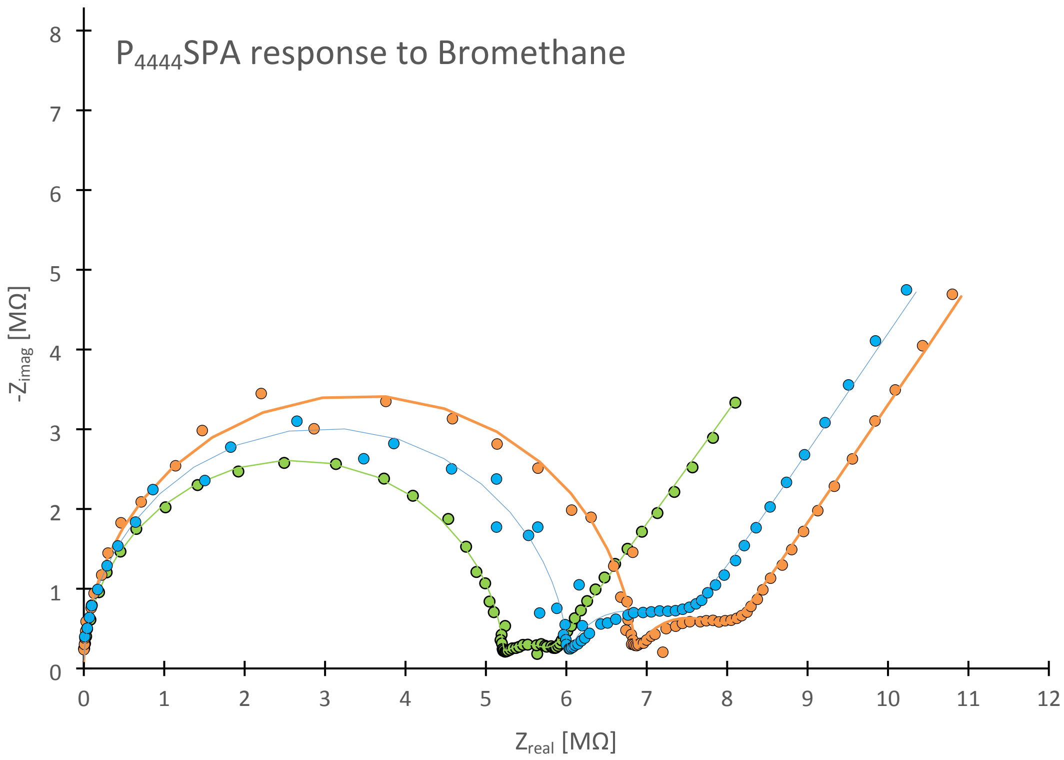

Figure S33. Measured data of $P_{4,4,4,4}$SPA (**1**) sensor response to synthetic air (green), acetonitrile (orange) and after exposure (blue).

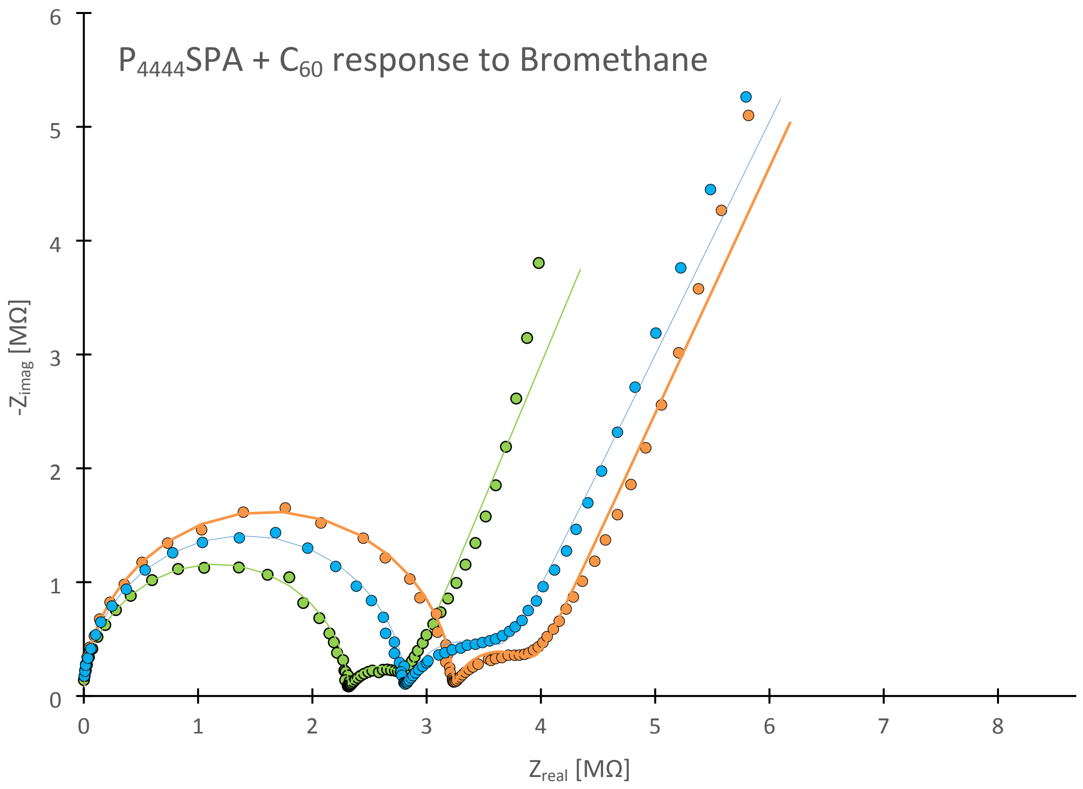

Figure S34. Measured data of $P_{4,4,4,4}$SPA + C_{60} (**1+C_{60}**) sensor response to synthetic air (green), bromoethane (orange) and after exposure (blue).

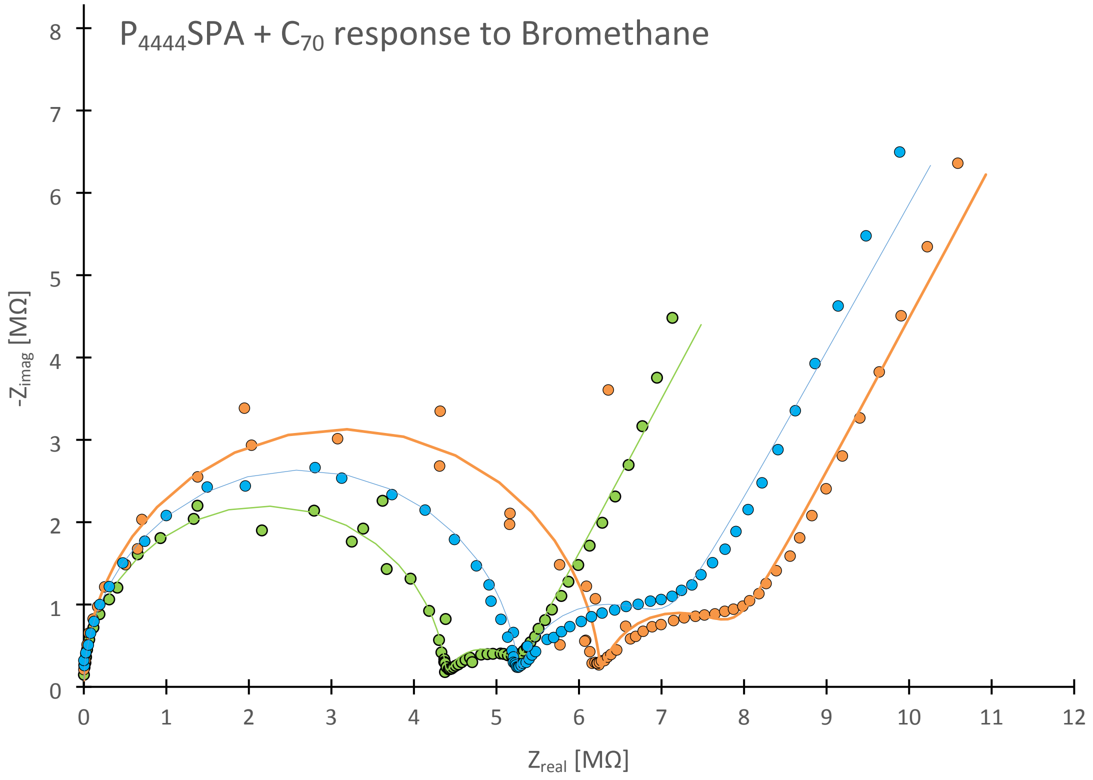

Figure S35. Measured data of $P_{4,4,4,4}$SPA + C_{70} (**1+C$_{70}$**) sensor response to synthetic air (green), bromoethane (orange) and after exposure (blue).

Table S11. Parameters evaluated using CPE model for bromoethane responses of sensors based on $P_{4,4,4,8}$SPA (**2**) and $P_{4,4,4,8}$SPA C_{60}/C_{70} (**2+C$_{60}$**, **2+C$_{70}$**) nanocomposites

**Sensor**	**[$P_{4,4,4,8}$][SPA] (2)**			**2 + C_{60}**			**2 + C_{70}**		
	**Ref.**	**BE***	**Ref.**	**Ref.**	**BE**	**Ref.**	**Ref.**	**BE**	**Ref.**
**$R_s \cdot 10^{-3}$ (Ω)**	< 1	134	1	272	5	294	80	24	3
**$R_p \cdot 10^4$ (Ω)**	55	79	70	84	124	91	52	134	177
**$R_{ct} \cdot 10^4$ (Ω)**	160	226	199	559	737	644	504	666	632
**$C_{dl} \cdot 10^{-14}$ (F)**	180	179	180	187	190	190	190	188	190
**$C_p \cdot 10^{-11}$ (F)**	1231	1674	1708	531	723	1305	598	1209	703
**$Y_0 \cdot 10^{-9}$ (S s$^{\alpha}$)**	**565**	**391**	**399**	**414**	**323**	**322**	**341**	**236**	**231**
**α**	**0.70**	**0.61**	**0.60**	**0.54**	**0.48**	**0.45**	**0.58**	**0.54**	**0.53**

*BE = bromoethane

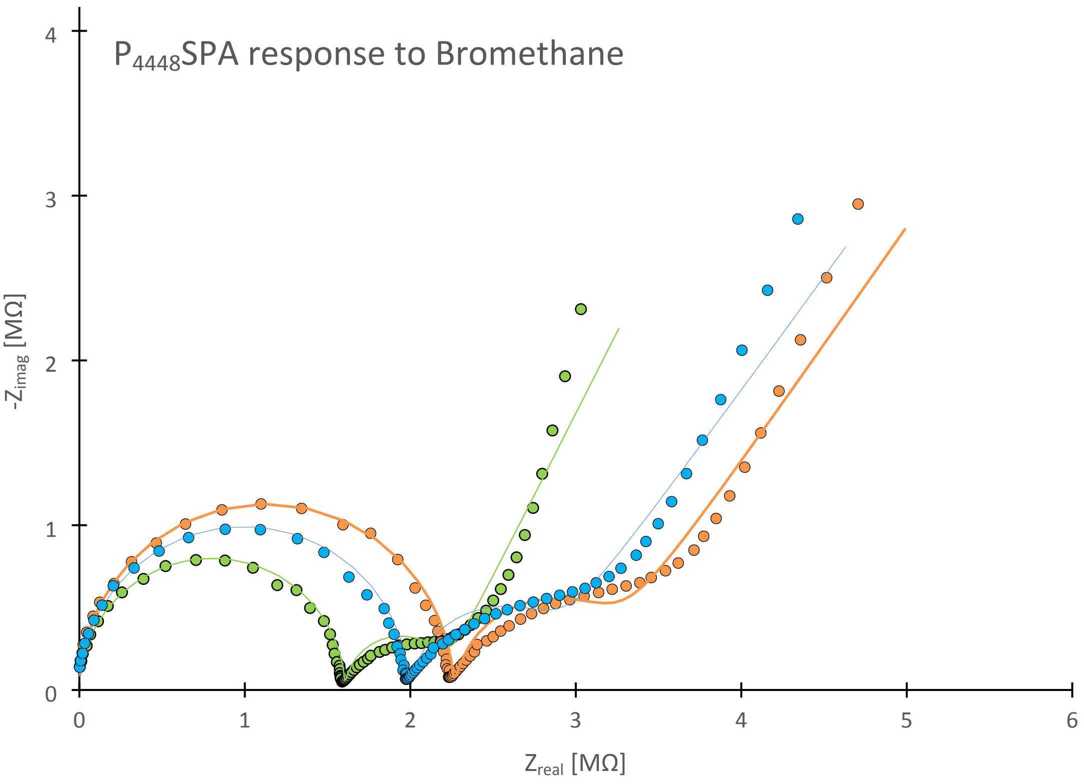

Figure S36. Measured data of $P_{4,4,4,8}$SPA (**2**) sensor response to synthetic air (green), bromoethane (orange) and after exposure (blue).

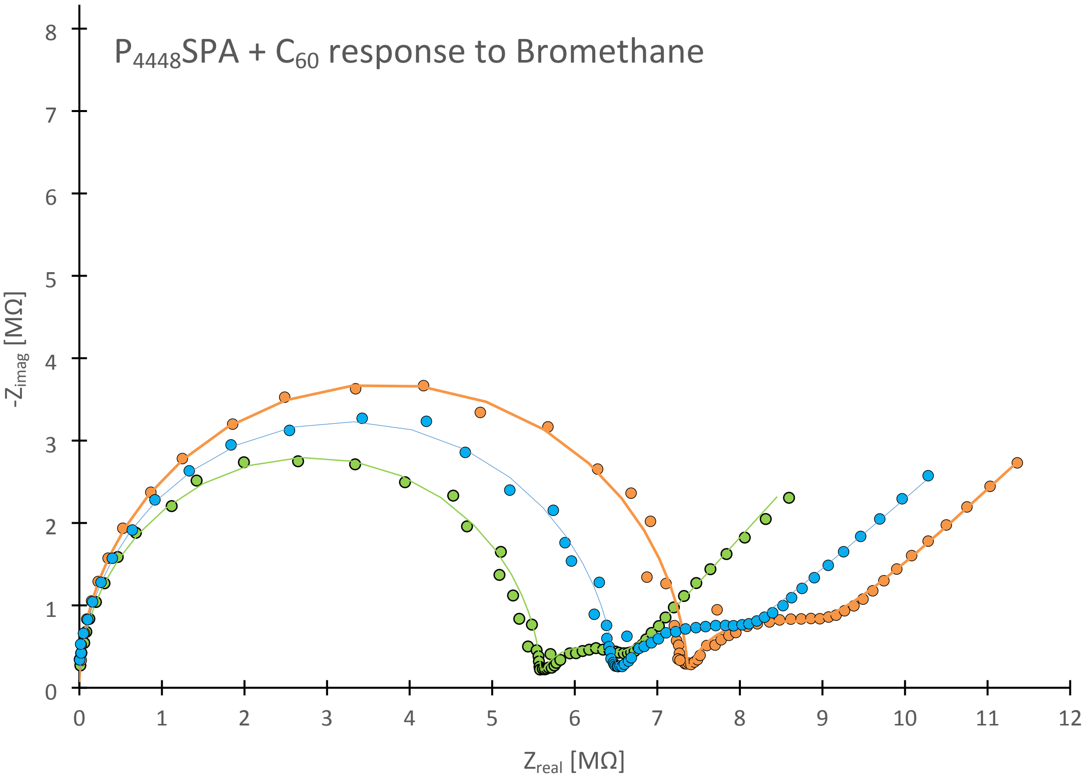

Figure S37. Measured data of $P_{4,4,4,8}$SPA + C_{60} (**2+C_{60}**) sensor response to synthetic air (green), bromoethane (orange) and after exposure (blue).

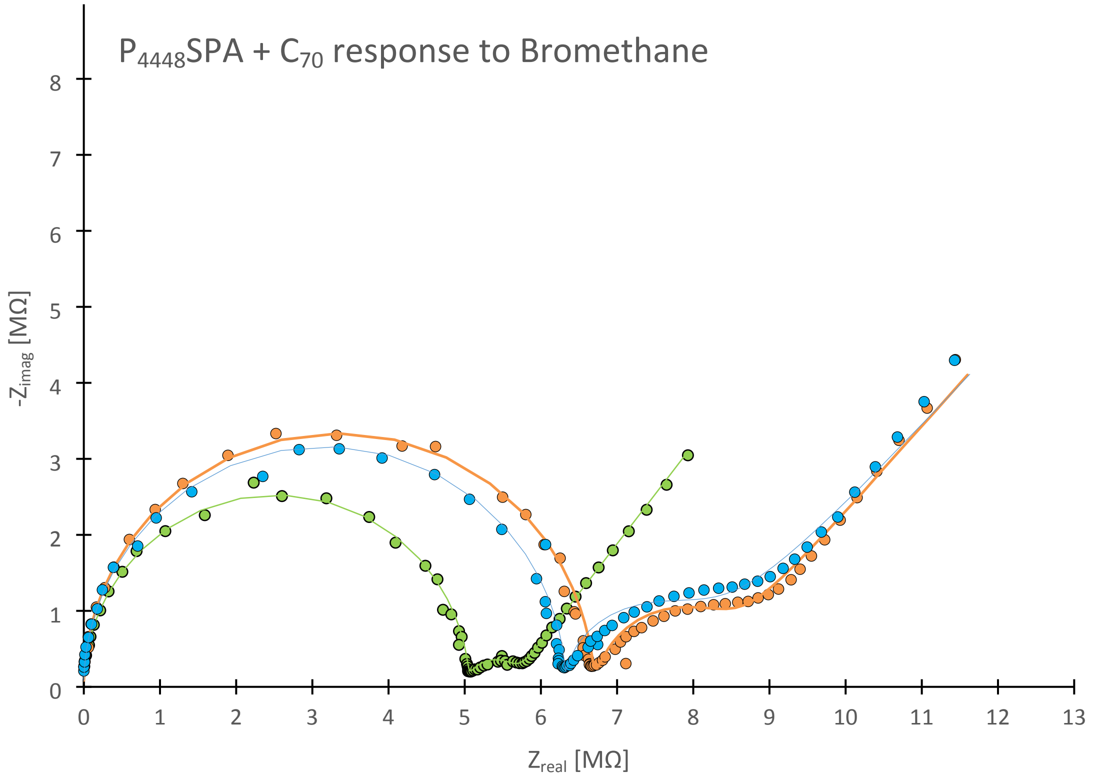

Figure S38. Measured data of $P_{4,4,4,8}$SPA + C_{70} (**2+C_{70}**) sensor response to synthetic air (green), bromoethane (orange) and after exposure (blue).

Table S12. Parameters evaluated using CPE model for ethanol responses of sensors based on $P_{4,4,4,4}$SPA (**1**) and $P_{4,4,4,4}$SPA C_{60}/C_{70} (**1+C_{60}**, **1+C_{70}**) nanocomposites.

Sensor	[$P_{4,4,4,4}$][SPA] (1)			1 + C_{60}			1 + C_{70}		
	Ref.	EtOH	Ref.	Ref.	EtOH	Ref.	Ref.	EtOH	Ref.
$R_s \cdot 10^{-3}$ (Ω)	3	160	621	133	157	45	64	16	2
$R_p \cdot 10^{4}$ (Ω)	112	150	120	34	46	42	54	73	66
$R_{ct} \cdot 10^{4}$ (Ω)	628	838	685	324	409	370	314	459	400
$C_{dl} \cdot 10^{-14}$ (F)	184	184	185	190	189	187	187	188	187
$C_p \cdot 10^{-11}$ (F)	541	387	534	1171	849	815	794	625	659
$Y_0 \cdot 10^{-9}$ (S s^{α})	**224**	**206**	**217**	**383**	**346**	**366**	**388**	**346**	**363**
α	**0.66**	**0.66**	**0.66**	**0.65**	**0.65**	**0.65**	**0.68**	**0.67**	**0.68**

*EtOH = ethanol

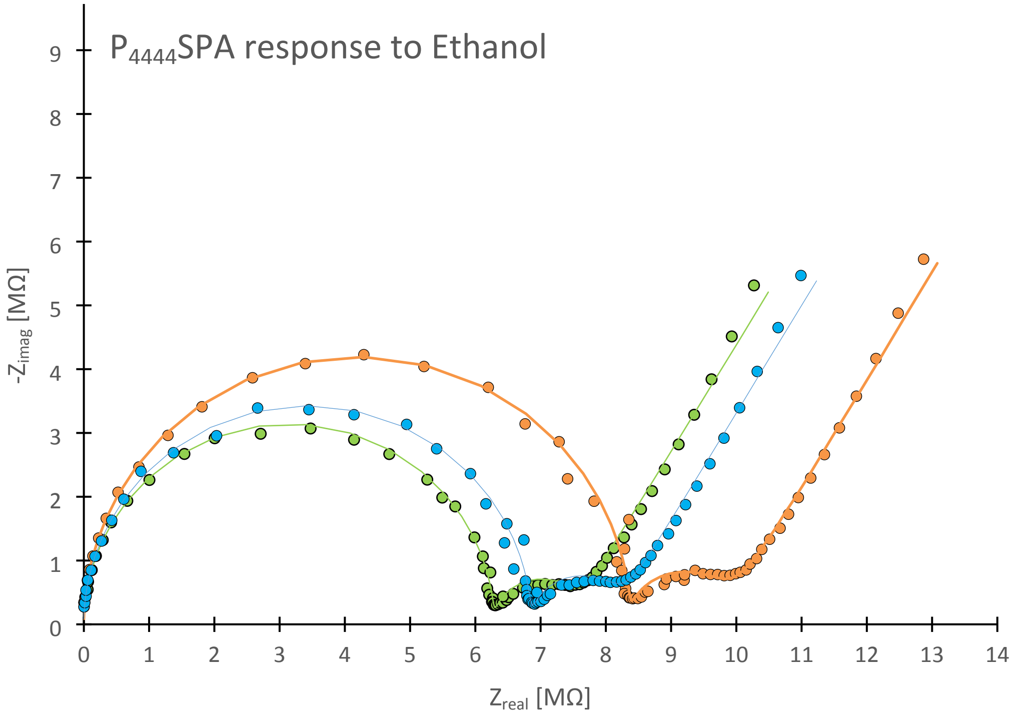

Figure S39. Measured data of $P_{4,4,4,4}$SPA (**1**) sensor response to synthetic air (green), ethanol (orange) and after exposure (blue).

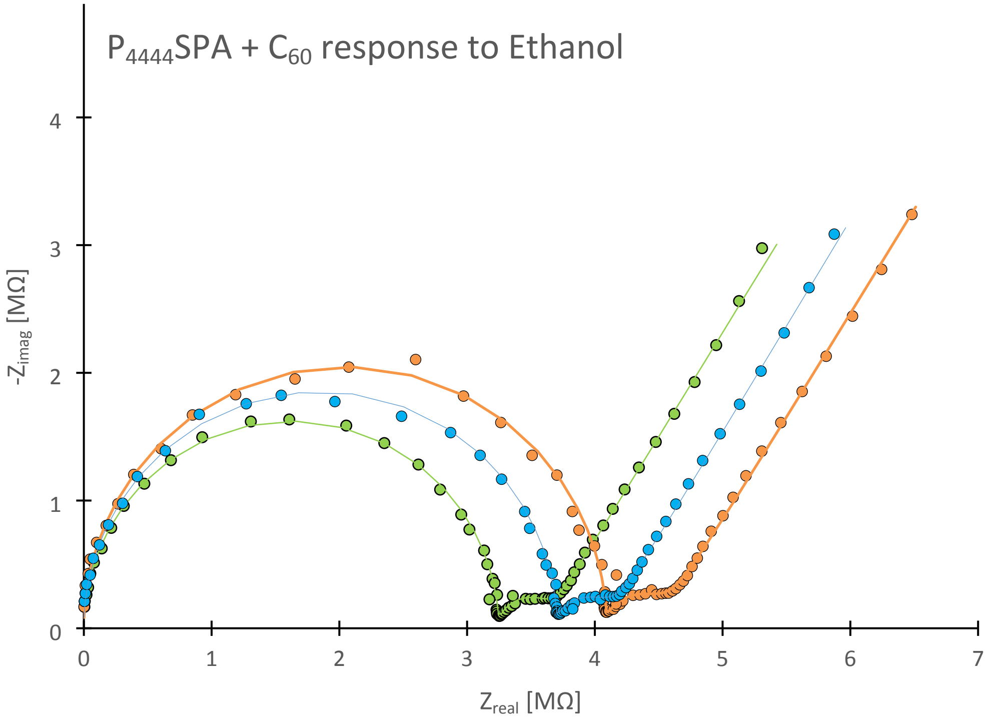

Figure S40. Measured data of $P_{4,4,4,4}$SPA + C_{60} (**1+C_{60}**) sensor response to synthetic air (green), ethanol (orange) and after exposure (blue).

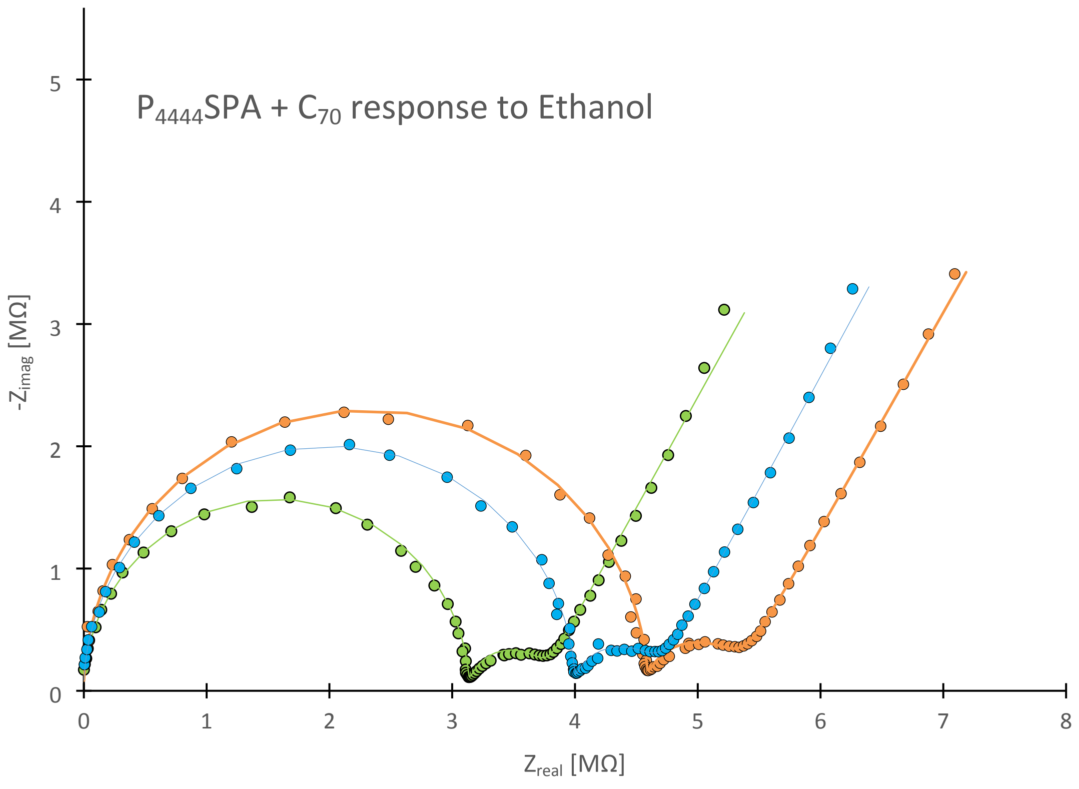

Figure S41. Measured data of $P_{4,4,4,4}$SPA + C_{70} (**1+C_{70}**) sensor response to synthetic air (green), ethanol (orange) and after exposure (blue).

Table S13. Parameters evaluated using CPE model for ethanol responses of sensors based on $P_{4,4,4,8}$SPA (**2**) and $P_{4,4,4,8}$SPA C_{60}/C_{70} (**2+C_{60}**, **2+C_{70}**) nanocomposites.

**Sensor**	**[$P_{4,4,4,8}$][SPA] (2)**			**2 + C_{60}**			**2 + C_{70}**		
	**Ref.**	**EtOH**	**Ref.**	**Ref.**	**EtOH**	**Ref.**	**Ref.**	**EtOH**	**Ref.**
**$R_s \cdot 10^{-3}$ (Ω)**	< 1	3	< 1	< 1	352	< 1	341	25	< 1
**$R_p \cdot 10^{4}$ (Ω)**	63	72	69	113	142	130	79	95	82
**$R_{ct} \cdot 10^{4}$ (Ω)**	291	377	351	400	552	489	350	542	466
**$C_{dl} \cdot 10^{-14}$ (F)**	189	191	189	188	188	190	208	198	199
**$C_p \cdot 10^{-11}$ (F)**	864	908	904	614	593	638	558	696	685
**$Y_0 \cdot 10^{-9}$ (S s^{α})**	**520**	**472**	**482**	**332**	**292**	**306**	**427**	**360**	**378**
**α**	**0.57**	**0.56**	**0.57**	**0.60**	**0.58**	**0.59**	**0.62**	**0.62**	**0.62**

*EtOH = ethanol

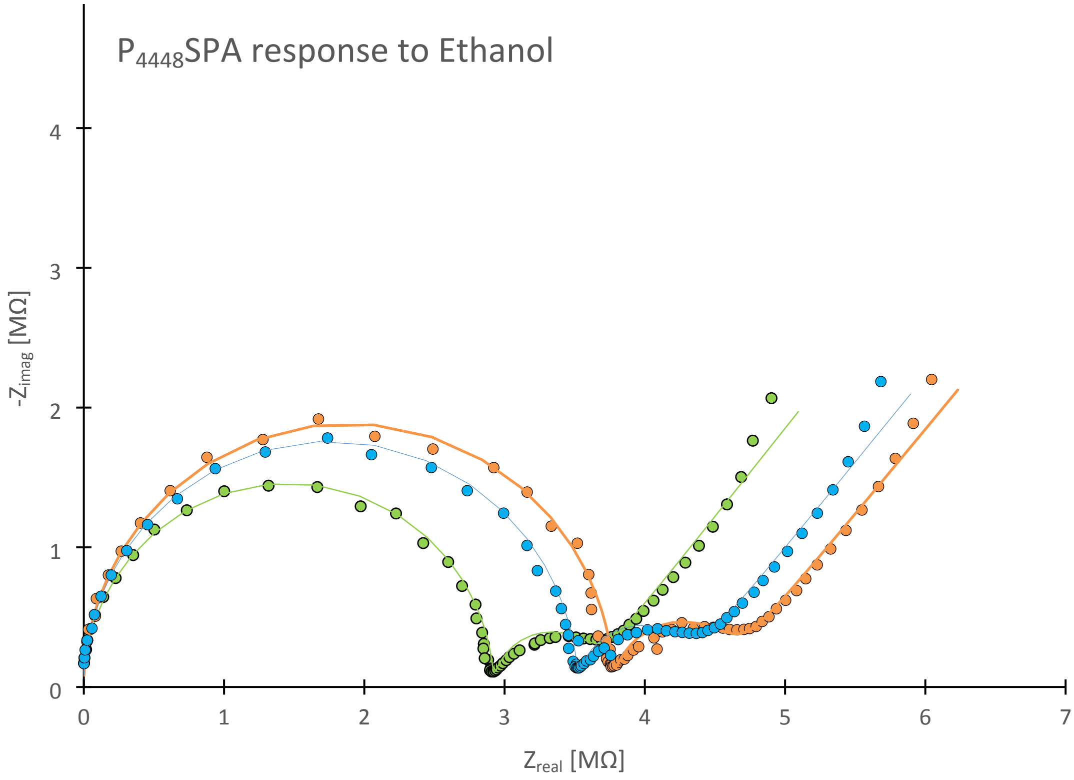

Figure S42. Measured data of $P_{4,4,4,8}$SPA (**2**) sensor response to synthetic air (green), ethanol (orange) and after exposure (blue).

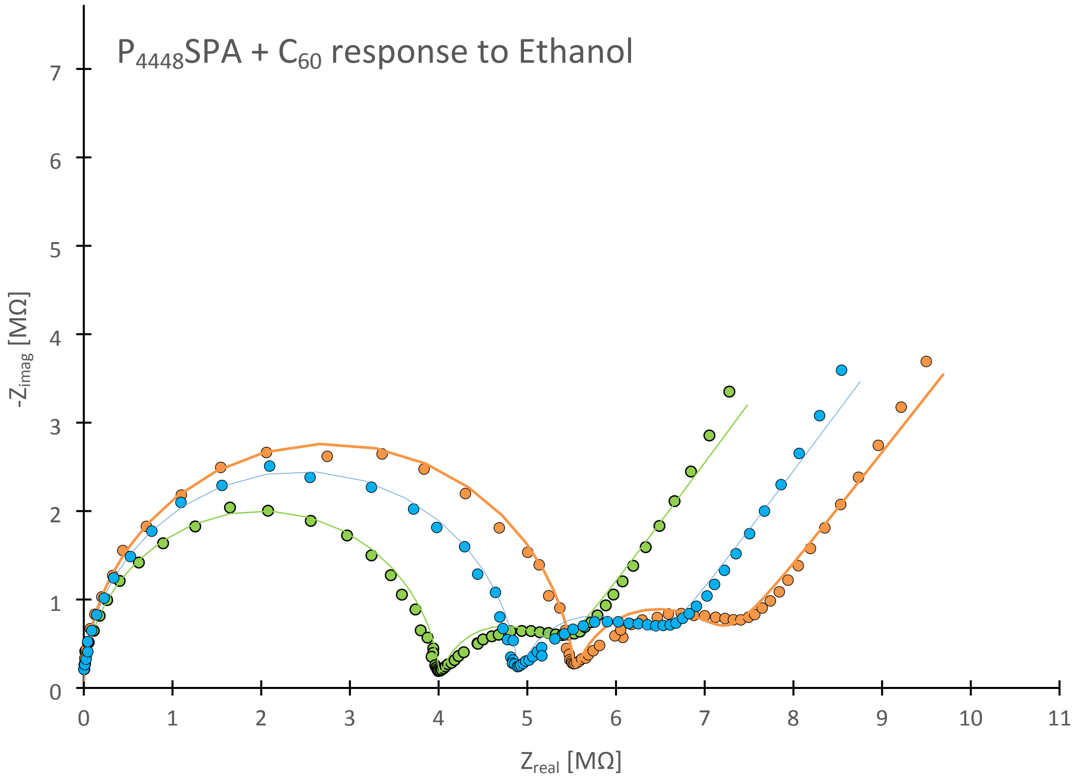

Figure S43. Measured data of $P_{4,4,4,8}$SPA + C_{60} (**2+C_{60}**) sensor response to synthetic air (green), ethanol (orange) and after exposure (blue).

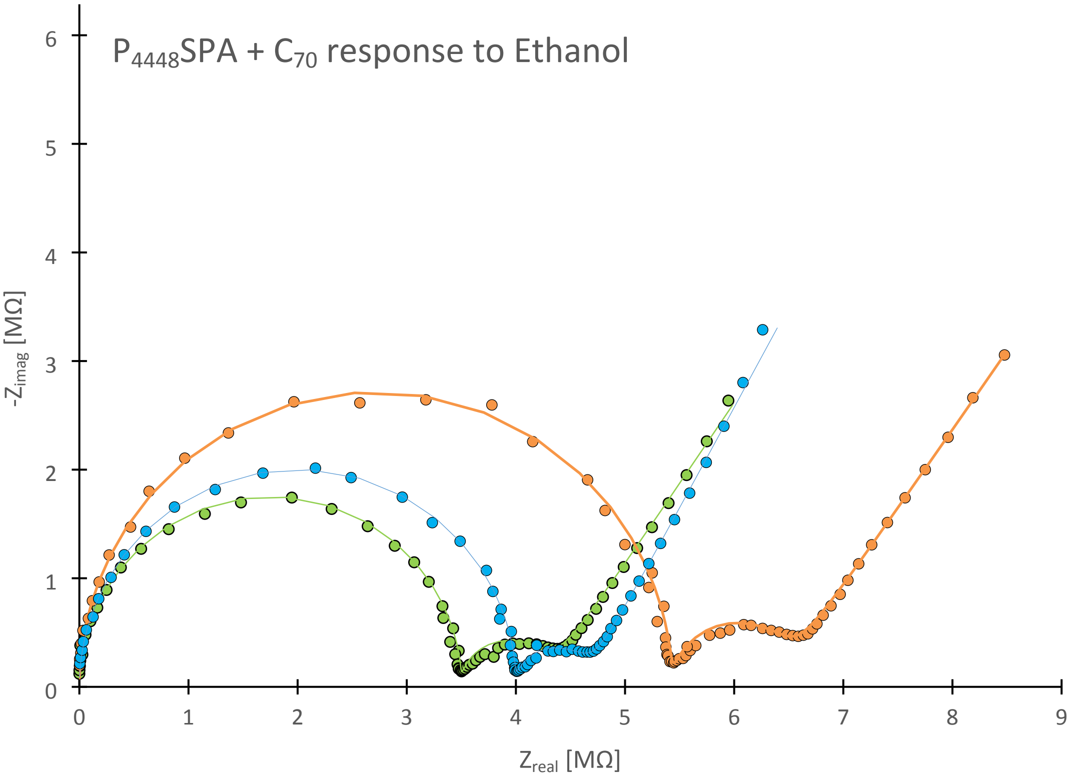

Figure S44. Measured data of $P_{4,4,4,8}$SPA + C_{70} (**2+C_{70}**) sensor response to synthetic air (green), ethanol (orange) and after exposure (blue).

Table S14. Physical properties of the used analytes (dipole moment μ, relative polarity E_T^N, permittivity $\boldsymbol{\varepsilon_r}$).

Analytes	Analyte properties		
	$\boldsymbol{\varepsilon_r}$	$\boldsymbol{\mu}$ **(D)** gaseous	E_T^N
**Acetaldehyde**	21.0[2] (18 °C)	2.69[3]	0.420[4]
**Acetic acid**	6.2[2] (20 °C)	1.74[3]	0.648[5]
**Acetonitrile**	36.6[2] (20 °C)	3.92[3]	0.460[5]
**Bromoethane**	9.0[2] (25 °C)	2.03[3]	0.213[5]
**Ethanol**	25.3[2] (20 °C)	1.69[3]	0.654[5]

The best fit was found for the relative permittivity $\boldsymbol{\varepsilon_r}$ (R^2 = 96.4%, Figure S45). The linear regression of the ΔY_0 on the dipole moment μ (R^2 = 55.6 %, Figure S46) shows a better fit than in the case of the relative polarity $\boldsymbol{E_T^N}$ (R^2 = 36.6%, Figure S47). Acetic acid was omitted in the fit due to its irreversible properties.

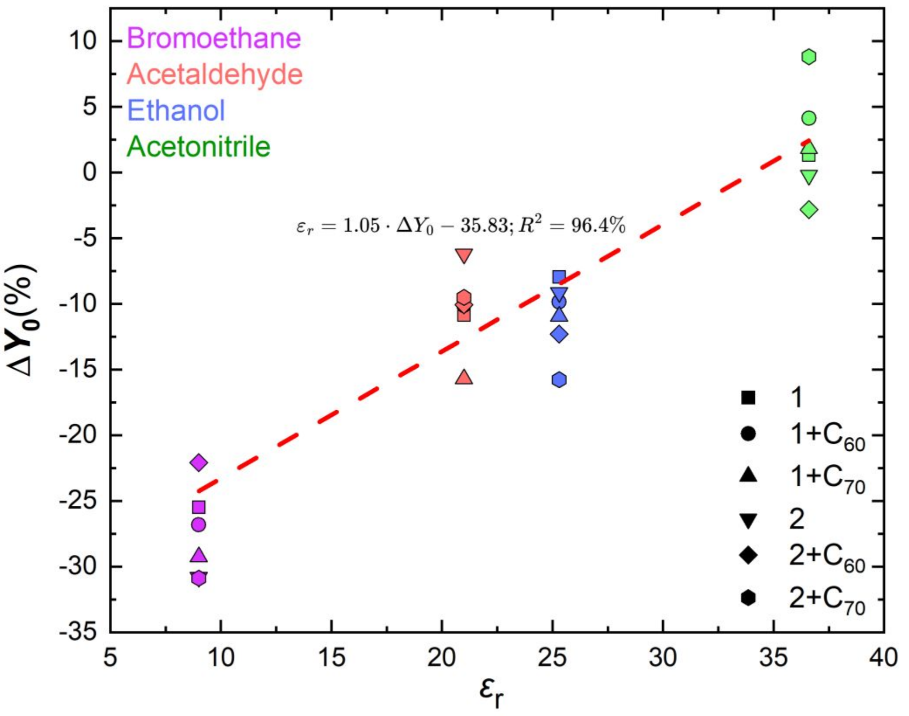

Figure S45. Linear dependence of the Y_0 value change on the relative permittivity of the analyte (acetic acid data excluded from linear regression).

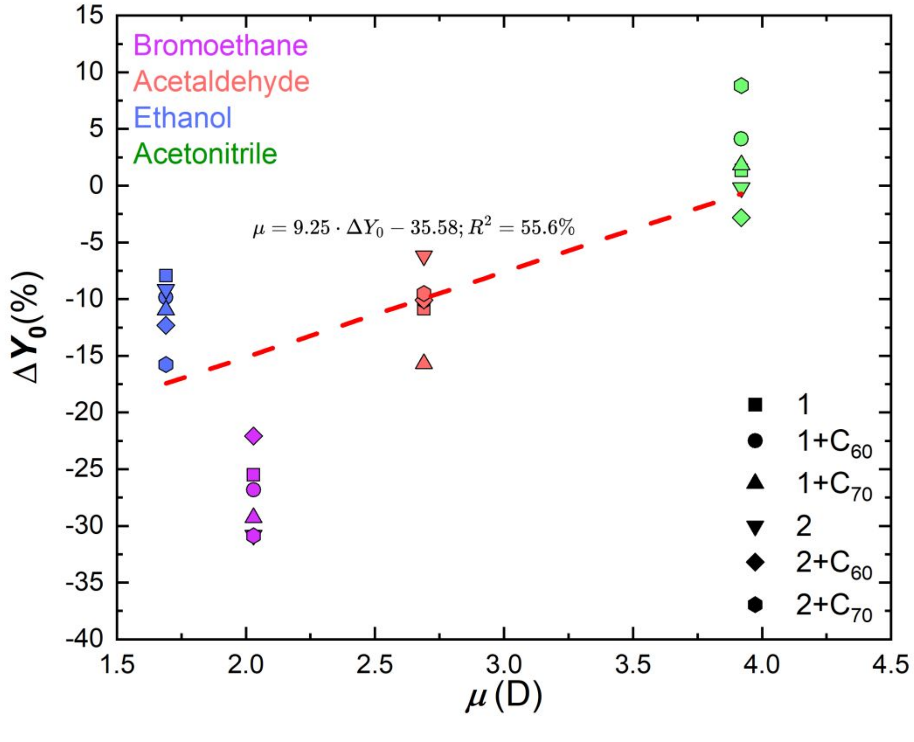

Figure S46. Linear dependence of the Y_0 value change on the dipole moment of the analyte (acetic acid data excluded from linear regression).

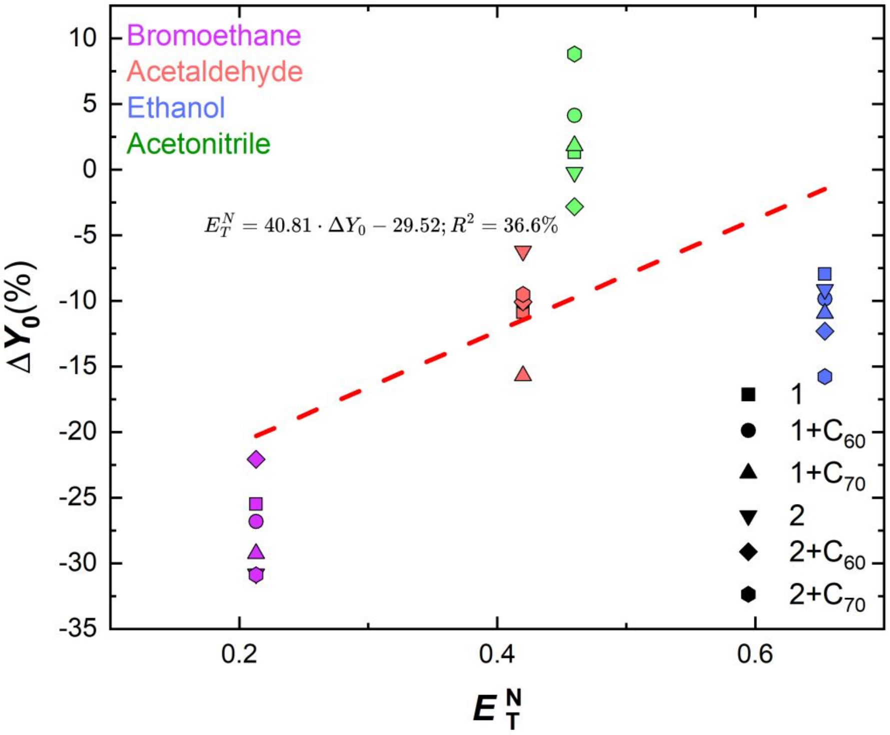

Figure S47. Linear dependence of the Y_0 value change on the relative polarity of the analyte (acetic acid data excluded from linear regression).

In our previous study, using $P_{4,4,4,6}$SPA towards 10 ppm of toxic gases: nitrogen dioxide (NO_2), methanol (MeOH), 4-bromoacetophenone (4-BAP), and diethylmalonate (DEM)[6], we found that the highest direct current response and highest phase angle sensitivity was observed for 4-BAP, followed by MeOH and DEM, while NO_2 exhibited the lowest. This sequence quantitatively correlated with the dipole moments (μ) of the analyte molecules: 4-BAPμ=3.1 D; MeOH μ=1.7 D; DEM μ=2.54 D; NO_2 μ=0.6 D.

For comparison, we used the sum of resistances (R_s, R_{ct}, and R_p) from the equivalent circuit to represent the direct current response (R_{dc}) from the previous study, allowing us to investigate the relationship between the equivalent circuit parameters and the gas-sensing behaviour observed in our earlier work. Contrary to our previous findings, the current results do not show a clear trend when comparing the resistance change (ΔR) to the dipole moments of the analytes (Figure S48). This indicates that other factors, such as the specific interactions between the analytes and the PIL materials or the structural differences between $P_{4,4,4,4}$SPA, $P_{4,4,4,8}$SPA, and $P_{4,4,4,6}$SPA, may play significant roles in determining the sensor responses.

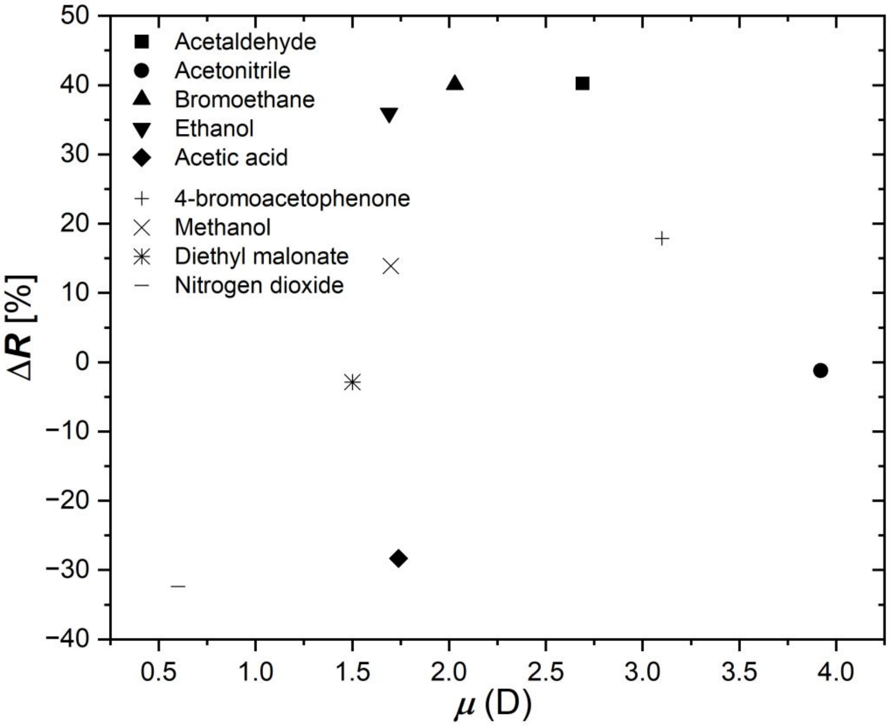

Figure S48. Dependence of the change in $P_{4,4,4,X}$SPA sensor resistance (ΔR) on the dipole moment (μ) of various gas analytes.

The plot compares the sensor responses of $P_{4,4,4,8}$SPA and $P_{4,4,4,4}$SPA to acetaldehyde, acetonitrile, bromoethane, ethanol, and acetic acid with the responses of $P_{4,4,4,6}$SPA to 4-bromoacetophenone, methanol, diethyl malonate, and nitrogen dioxide from our previous study[6].

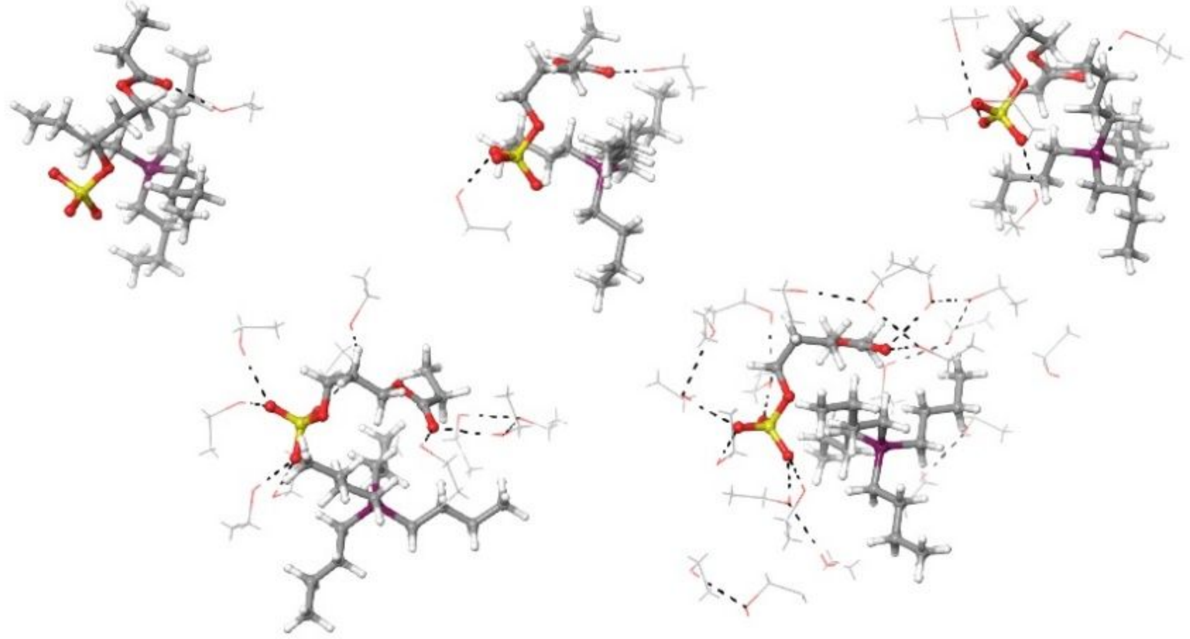

Figure S49. Interaction of $P_{4,4,4,4}$SPA upon sequential addition of explicit ethanol solvent molecules (1, 2, 5, 10, 20).

Hydrogen bonds are indicated by black dashed lines. The phosphonium-polymer (P-S) distances of 4.58, 4.56, 4.72, 4.58 and 4.75 Å, respectively, are almost unchanged from that of the isolated complex (4.75 Å).

References

[1] S. Martins, A. Fedorov, C. A. M. Afonso, C. Baleizão, M. N. Berberan-Santos, *Chem Phys Lett* **2010**, *497*, 43–47.

[2] W. M. Haynes, *CRC Handbook of Chemistry and Physics*, CRC Press, **2014**.

[3] C. L. Yaws, P. K. Narasimhan, in *Thermophysical Properties of Chemicals and Hydrocarbons* (Ed.: C.L. Yaws), William Andrew Publishing, Norwich, NY, **2009**, pp. 672–682.

[4] A. D. Walsh, *Transactions of the Faraday Society* **1947**, *43*, 158–163.

[5] C. Reichardt, *Chem Rev* **1994**, *94*, 2319–2358.

[6] E. Marešová, D. Tomeček, P. Fitl, J. Vlček, M. Novotný, L. Fišer, Š. Havlová, P. Hozák, A. Tudor, T. Glennon, L. Florea, S. Coyle, D. Diamond, Z. Skaličan, M. Hoskovcová, M. Vrňata, *Sensors and actuators. B, Chemical* **2018**, *266*, 830–840.
